# Investigating the effects of lycopene and green tea on the metabolome of men at risk of prostate cancer: The ProDiet randomised controlled trial

**DOI:** 10.1002/ijc.31929

**Published:** 2018-12-07

**Authors:** Rhona A. Beynon, Rebecca C. Richmond, Diana L. Santos Ferreira, Andrew R. Ness, Margaret May, George Davey Smith, Emma E. Vincent, Charleen Adams, Mika Ala‐Korpela, Peter Würtz, Sebastian Soidinsalo, Christopher Metcalfe, Jenny L. Donovan, Athene J. Lane, Richard M. Martin, the ProtecT Study Group, The PRACTICAL consortium

**Affiliations:** ^1^ Present address: Population Health Sciences, Bristol Medical School University of Bristol Bristol United Kingdom; ^2^ Medical Research Council Integrative Epidemiology Unit at the University of Bristol Bristol United Kingdom; ^3^ The National Institute for Health Research (NIHR) Bristol Biomedical Research Centre Upper Maudlin Street, Bristol United Kingdom; ^4^ School of Cellular and Molecular Medicine University of Bristol Bristol United Kingdom; ^5^ Computational Medicine University of Oulu and Biocenter Oulu Oulu Finland; ^6^ NMR Metabolomics Laboratory, School of Pharmacy University of Eastern Finland Kuopio Finland; ^7^ Systems Epidemiology Baker Heart and Diabetes Institute Melbourne Victoria Australia; ^8^ Department of Epidemiology and Preventive Medicine, School of Public Health and Preventive Medicine, Faculty of Medicine, Nursing and Health Sciences The Alfred Hospital, Monash University Melbourne Victoria Australia; ^9^ Research Programs Unit, Diabetes and Obesity University of Helsinki Helsinki Finland; ^10^ Nightingale Health Ltd. Helsinki Finland; ^11^ Bristol Randomised Trials Collaboration, School of Social and Community Medicine University of Bristol Bristol United Kingdom

**Keywords:** prostate cancer, dietary intervention, lycopene, green tea, Mendelian randomisation

## Abstract

Lycopene and green tea consumption have been observationally associated with reduced prostate cancer risk, but the underlying mechanisms have not been fully elucidated. We investigated the effect of factorial randomisation to a 6‐month lycopene and green tea dietary advice or supplementation intervention on 159 serum metabolite measures in 128 men with raised PSA levels (but prostate cancer‐free), analysed by intention‐to‐treat. The causal effects of metabolites modified by the intervention on prostate cancer risk were then assessed by Mendelian randomisation, using summary statistics from 44,825 prostate cancer cases and 27,904 controls. The systemic effects of lycopene and green tea supplementation on serum metabolic profile were comparable to the effects of the respective dietary advice interventions (*R* ^2^ = 0.65 and 0.76 for lycopene and green tea respectively). Metabolites which were altered in response to lycopene supplementation were acetate [***β*** (standard deviation difference *vs*. placebo): 0.69; 95% CI = 0.24, 1.15; *p* = 0.003], valine (*β*: −0.62; −1.03, −0.02; *p* = 0.004), pyruvate (*β*: −0.56; −0.95, −0.16; *p* = 0.006) and docosahexaenoic acid (*β*: −0.50; −085, −0.14; *p* = 0.006). Valine and diacylglycerol were lower in the lycopene dietary advice group (β: −0.65; −1.04, −0.26; *p* = 0.001 and *β*: −0.59; −1.01, −0.18; *p* = 0.006). A genetically instrumented SD increase in pyruvate increased the odds of prostate cancer by 1.29 (1.03, 1.62; *p* = 0.027). An intervention to increase lycopene intake altered the serum metabolome of men at risk of prostate cancer. Lycopene lowered levels of pyruvate, which our Mendelian randomisation analysis suggests may be causally related to reduced prostate cancer risk.

## Abstract 2

**What's new?**

Prostate cancer incidence varies by geographic region, suggesting that environmental factors, such as diet, play a role. Here, the authors investigated how green tea and lycopene intake affects prostate cancer risk. They conducted a 6‐month intervention on men with raised PSA levels but no cancer, testing levels of 159 serum metabolites by NMR. Lycopene supplementation, they found, reduced levels of circulating pyruvate, and Mendelian randomisation analysis suggests pyruvate may boost PC risk. These results suggest a possible mechanism of action by which consuming dietary lycopene may reduce prostate cancer risk.

**Abbreviations**
2LSR: 2 least‐squares regression
BMI: body mass index
CI: confidence interval
DHA: docosahexaenoic acid
ECGC: epigallocatechin‐3‐gallate
FA: fatty acid
GCKR: glucokinase regulatory protein
IV: instrumental variable
MCT2: monocarboxylate transporter 2
MR: Mendelian randomisation
NMR: Nuclear magnetic resonance
PCA: principle component analysis
PCs: principle components
PDPR: pyruvate dehydrogenase phosphatase regulatory
PSA: prostate specific antigen
PUFA: polyunsaturated fatty acids
RCT: Randomised controlled trial
SD: standard deviation
SNP: single nucleotide polymorphism

## Introduction

Prostate cancer is the second most common cancer diagnosed in males worldwide.^1^ The burden of the disease is not evenly distributed however, with “Western Countries” like the United States, Western Europe and Australia experiencing the highest incidences and Asia the lowest.^1^ Given this geographical variation, lifestyle factors are thought to influence prostate cancer risk^2^ and there has been growing interest in studying the impact of dietary changes on prostate cancer incidence.

Several dietary factors have been purported to modulate prostate cancer risk.^3^, ^4^ Green tea and lycopene, a bright‐red carotenoid found primarily in tomatoes, have received particular attention. This is largely because of their potent antioxidant activity *in vitro,* 5, 6 although other chemopreventative mechanisms have been suggested.^7^, ^8^ Epidemiological evidence that lycopene and green tea protect against prostate cancer is inconsistent however. Of the three published meta‐analyses that have considered the association of lycopene intake with prostate cancer, two report an inverse association,^9^, ^10^ while the other found insufficient evidence to either support or refute the use of lycopene for the prevention of prostate cancer.^11^ An initial meta‐analysis suggested that green tea consumption may have a protective effect, especially in Asian populations,^12^ but this finding was not supported in a more recent meta‐analysis.^13^

The results of observational studies of diet and cancer risk must be interpreted with caution as they are susceptible to confounding^14^ and measurement error in the reporting of dietary exposures, mainly due to recall and reporting bias.^15^ Such issues can be overcome using well‐designed and conducted randomised controlled trials (RCTs).

We recently reported the primary results of a randomised, placebo‐controlled factorial trial of lycopene and green tea in men at elevated risk of prostate cancer (ProDiet).^16^ Post‐intervention plasma lycopene and epigallocatechin‐3‐gallate (ECGC) (a bioactive component of green tea) levels were increased in the respective intervention arms, indicating high levels of adherence. In the present study, we investigated the metabolic effects of lycopene and green tea intake in men enrolled in the same trial, using nuclear magnetic resonance (NMR)‐based metabolic profiling. Where evidence of an effect of one of the interventions on metabolic measures was found, we used Mendelian randomisation (MR) to assess the causal role of these metabolic traits on prostate cancer risk. MR is a technique that uses genetic variation to proxy exposures of interest to circumvent issues of reverse causation and confounding that bias observational epidemiology.^17^, ^18^ Formal MR approaches have been used previously in this context, to capture causal relationships between metabolites and disease,^19^, ^20^, ^21^, ^22^ facilitated by strong genotypic effects on metabolites.^23^, ^24^, ^25^, ^26^, ^27^

## Materials and Methods

### Overview

**Figure 1:**
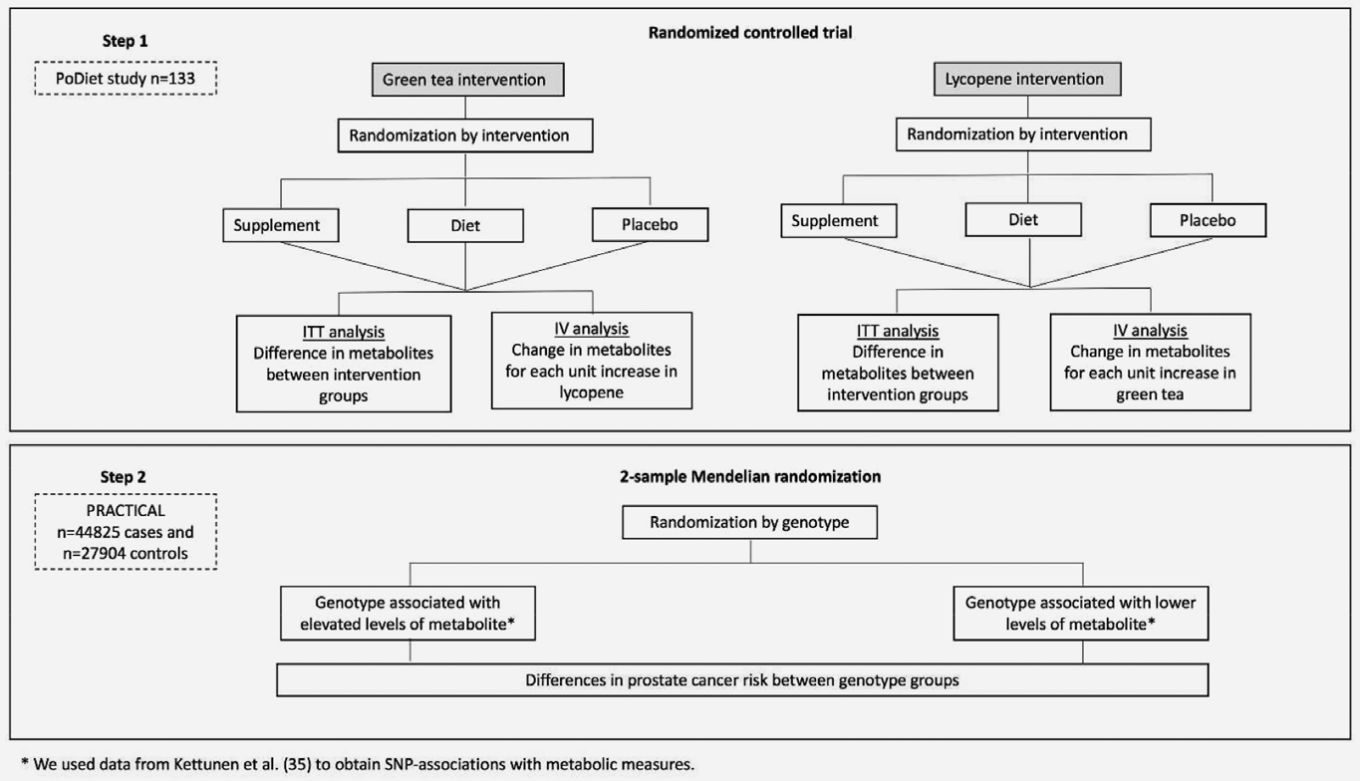
Analysis steps for investigating the effects of lycopene and green tea on serum metabolome of men at risk of prostate cancer, and the causal role of altered metabolic traits on prostate cancer risk. We conducted 2 analyses. In stage one, relationships between metabolic measures and lycopene or green tea randomisation arms were tested using an intention‐to‐treat analyses. In stage two, we used GWAS summary statistics from Kettunen *et al* to identify genetic variants that could be used as instrumental variables for the effects of metabolites on prostate cancer risk. Data on the association of these genetic variants with prostate cancer risk were obtained from the PRACTICAL consortium (44,825 prostate cancer cases and 27,904 controls of European ancestry). Data on the association of genetic variants with metabolite levels and with prostate cancer risk were combined to estimate the influence of metabolites on prostate cancer risk. ITT, intention‐to treat; IV, instrumental variable.

The present report includes two separate but interlinked investigations (Fig. 1):

1. An intention‐to‐treat analysis of the ProDiet factorial RCT. This assessed the effects on serum metabolome of interventions to increase green tea and lycopene intake;
2. A two‐sample Mendelian randomisation analysis^28^ using summary statistics data from the PRACTICAL (Prostate Cancer Association Group to Investigate Cancer Associated Alterations in the Genome) consortium.^29^ This investigated the causal effects of those metabolic measures shown to be altered by the interventions on prostate cancer risk.

### Effect of lycopene and green tea on serum metabolome

#### Study population

The ProDiet RCT (ISRCTN 95931417) included 133 men between the ages of 50 and 69 years with elevated prostate specific antigen (PSA) levels (results between 2.0 and 2.95 ng/mL or at least 3.0 ng/mL with a negative biopsy), who were identified as part of the community‐based PSA testing in the ProtecT (Prostate cancer testing and Treatment) study.^30^

#### Study design

**Figure 2:**
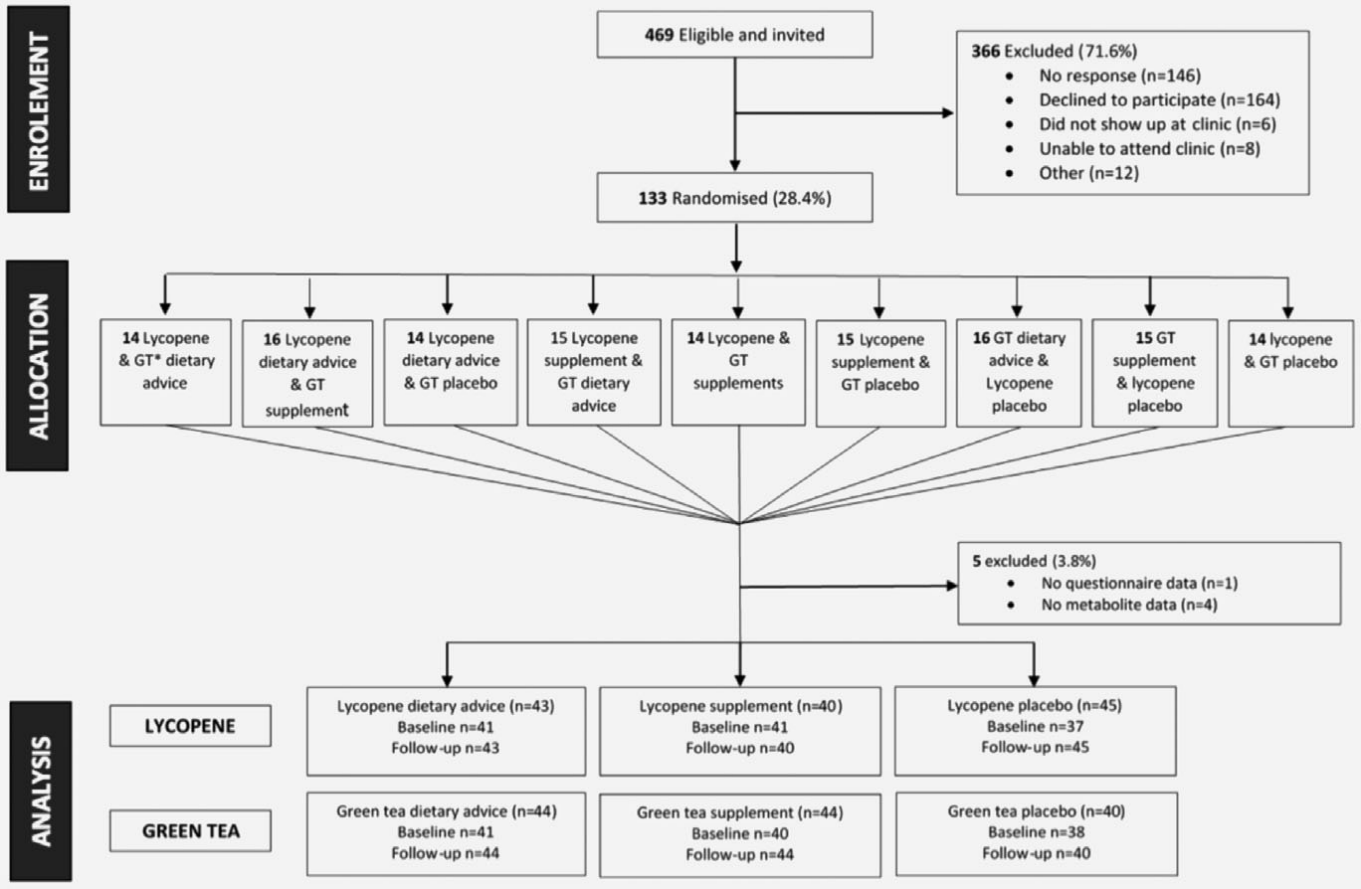
Flow of ProDiet participants through the study.Adapted from the main ProDiet study (Lane, AJ., unpublished), with thanks.

Full details of the trial have been provided in the Supporting Information (available online). Briefly, ProDiet was a feasibility randomised‐controlled trial of dietary interventions for prostate cancer prevention. Men were randomised to a daily lycopene arm (active capsules or lycopene‐rich diet or placebo capsules) and a green tea arm (active capsules or green tea drink or placebo capsules) for 6 months^16^ in a 3 × 3 factorial design (Fig. 2).

#### Data collection

At recruitment, trained nurses collected information on the men's weight, blood pressure, socio‐economic status and medical history.^16^, ^31^ Men were asked to complete a lifestyle questionnaire, which included questions on smoking, alcohol consumption and dietary intake of supplements or vitamins. Dietary intake was further assessed using a 117‐item food frequency questionnaire (FFQ), which was adapted from the UK arm of the EPIC study^32^ (see Supporting Information Methods). During the same clinic appointment, non‐fasted blood samples were drawn for baseline PSA, lycopene, EGCG and metabolic profiling, according to a standard protocol. Six‐months after randomisation, participants attended a follow‐up appointment, where repeat non‐fasted blood samples were taken. Samples were left at room temperature to clot and then centrifuged at 1640*g* for 20 min within 2 h of collection. They were kept at 5 °C during transportation to the laboratory, where they were aliquoted for storage at −80 °C within 36 h of collection. Serum lycopene levels were measured using reversed‐phase high‐performance liquid chromatography (HPLC).^33^ Plasma EGCG levels were analysed and quantified using HPLC‐mass spectroscopy (MS), as described by Stalmach *et al*.^34^

#### Measurement of metabolites

Metabolic profiling was performed using a high‐throughput serum nuclear magnetic resonance (NMR) metabolomics platform (Nightingale Health®, Helsinki, Finland), originally described by Soininen *et al*.^35^ A brief overview of the platform can be found in the Supporting Information methods.

Full details of the protocol and protocol, including information on quality control procedures, have been published elsewhere.^36^ In total, 159 metabolic traits were measured (see Supporting Information Table 8), including several amino acids (alanine, glutamine, glycine, histidine, isoleucine, leucine, valine, phenylalanine and tyrosine), glycolysis measures (glucose, lactate, pyruvate, citrate and glycerol), ketone bodies (acetate, acetoacetate and 3‐hydroxybutyrate), inflammatory markers (glycoprotein acetyls) and fatty acids (polyunsaturated, monounsaturated, linoleic, omega‐3, omega 6 and docosahexaenoic fatty acids), fatty acids traits (chain length, degree of unsaturation), as well as particle concentrations and lipid compositions of 14 lipoprotein subclasses (including low, intermediate, large and very large lipoprotein subclasses). In addition, fatty acids were also expressed as ratios (%) to total fatty acids. This set of metabolite traits are from multiple metabolic pathways, including those involved in carcinogenesis.

#### Statistical analysis

The effects of lycopene and green tea dietary interventions were examined in an intention‐to‐treat analysis, by comparing metabolic measures at 6‐months follow‐up for each intervention. To allow comparison of magnitudes of association across measures with different units, all metabolite concentrations were converted to standard deviation (z) scores. Linear regression was used to compare standardised (*z*‐scored) 6‐month metabolite measures across lycopene and green tea intervention groups, treating placebo as the reference category. As some metabolite concentrations had right skewed distributions, robust standard errors were estimated for all associations. In our primary analysis, no covariates were included in the models, as confounders were shown to be well balanced across the intervention arm (Supporting Information Tables 1 and 2). The overall match between the metabolic changes associated with supplement‐advice (*vs*. placebo) and the metabolic changes associated with dietary‐advice (*vs*. placebo) were assessed using linear regression, separately for both lycopene and green‐tea arms. The correspondence between respective supplement and dietary‐advice associations were assessed using the R^2^ statistic.

Given that many metabolic measures were analysed, the probability of finding evidence of association by chance (i.e., false positive) was high. Principle Component Analyses (PCA) was carried out on standardised metabolic measures data and used to set a significance threshold that takes into account both multiple testing and the correlation between metabolic traits,^37^ as discussed previously.^19^, ^37^, ^38^ This method assumes that the independence of the principle components (PCs) is equivalent to the degree of freedom of the original metabolic dataset, and that retaining a number of PCs that is enough to explain at least 95% of the variance will only result in a small chance of type 1 error. The first 14 principal components explained >95% of the variance in the metabolic measures data. We therefore set our significance threshold as *p* < 0.05/14 (=0.0036). *p*‐Values below this can be interpreted as providing strong evidence of an association of the respective intervention on metabolic trait levels.

Whilst randomisation aims to prevent bias in the allocation of participants to intervention arms in a RCT, this does not guarantee that groups will be comparable with respect to baseline measures, particularly in small feasibility trials. As a sensitivity analysis, we repeated the intention‐to‐treat analysis adjusting for pre‐intervention metabolic measures. All individuals with complete baseline and follow‐up metabolite data were included in the sensitivity analyses.

To establish the causal effect of lycopene and EGCG dose on metabolite measures at follow‐up, we employed instrumental variable (IV) analysis,^39^ using intervention status as an IV. The IV analysis was performed using a 2‐stage least squares (2SLS) regression method, implemented using the “ivreg2” function in Stata. F‐statistics and R^2^ values from the first‐stage regression between intervention arm and serum lycopene/EGCG levels were examined to check the instrumental variable assumption that the instrument is sufficiently associated with the exposure. Causal estimates for the instrumented effect of serum lycopene/EGCG levels on each follow‐up metabolite were obtained from the second‐stage regression. The regression coefficients were calculated in units of 1‐SD metabolite concentration per one‐unit increment in lycopene (μmol/L) or EGCG (nM). Associations were adjusted for baseline metabolic measures.^40^

Analyses were performed in Rstudio and Stata version 14.2.

### Causal effect of altered metabolites on prostate cancer risk

Given evidence that the dietary interventions were associated with changes in some of the metabolic measures at follow‐up, we used MR to investigate whether these metabolites could have a causal role in mediating the effect of the dietary interventions on prostate cancer risk.

MR is a form of IV analysis that uses genetic variants as instruments to examine the causal effects of modifiable exposures on outcomes of interest.^17^, ^41^ This method depends on the existence of genetic variants that are robustly associated with metabolite levels (see Supporting Information materials).

We utilised the two‐sample MR approach, described in more detail in the Supporting Information Methods. Briefly, genetic variants robustly associated with the serum metabolites of interest were first identified using data from a recently published genome‐wide association study (GWAS) of 123 circulating metabolite levels.^27^ These genetic instruments were analysed in relation to prostate cancer risk in a series of 44,825 prostate cancer cases and 27,904 control subjects GWAS data from the PRACTICAL consortium.^29^ PRACTICAL samples were genotyped using an Illumina.

Custom Infinium genotyping array (OncoArray), details of which may be found on their website http://practical.icr.ac.uk/blog/?page_id=1244). Two types of sensitivity analyses were undertaken to assess potential horizontal pleiotropic effects: a weighted median approach and MR‐Egger regression.^42^

## Results

One hundred and thirty‐three men were recruited and randomised into lycopene and green tea intervention groups (Fig. 2). One hundred and thirty‐two men attended the six‐month follow‐up appointment. Not all participants had sufficient blood available for metabolic profiling in the current study; metabolic measures were available for 119 men at baseline and 128 men at follow‐up.

### Baseline characteristics

Supporting Information Tables 1 and 2 show the baseline characteristics of the men stratified by lycopene and green tea treatment groups, respectively. Men were a mean age of 64.5 years (SD 5.0), had a mean BMI of 27.0 kg/m^2^ and had a median baseline PSA level of 2.6 ng/mL. There were no apparent differences across intervention arms with respect to any of the sociodemographic or lifestyle variables considered. Two of the men, randomised to lycopene and green tea supplement arms had diabetes.

Among the 116 men with complete baseline metabolic measures data, there were no clear differences in metabolic traits between the lycopene randomisation arms pre‐intervention (Supporting Information Table 3). At baseline, there was evidence of by‐arm differences in glycine, phenylalanine, alanine, glycoprotein acetyls and a number of fatty acid metabolic measures in the green tea group (Supporting Information Table 3).

### Difference in metabolite levels between intervention groups at six‐month follow‐up

#### Correlation between metabolic profiles from lycopene dietary advice and supplement arms

**Figure 3:**
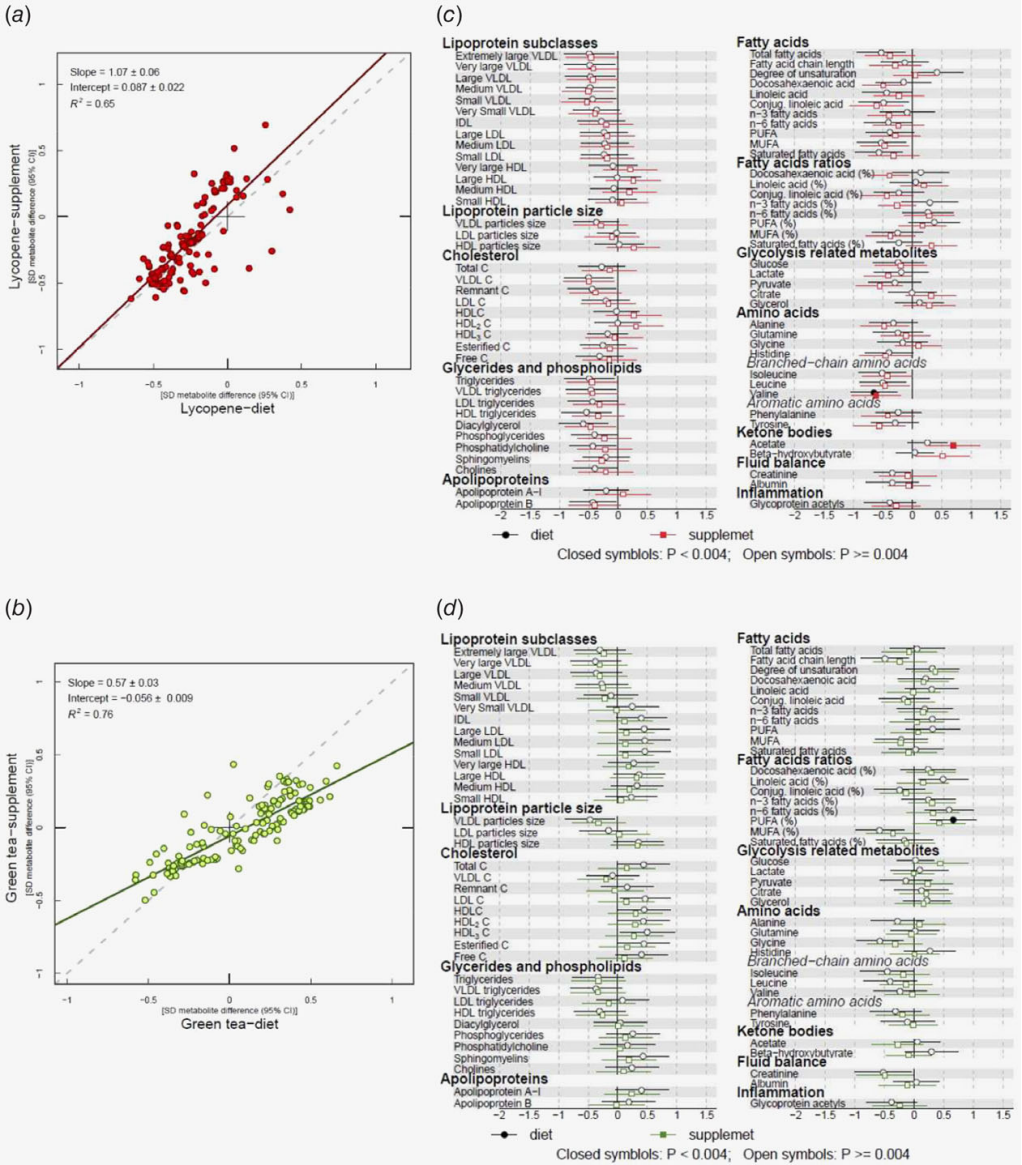
(*a*) Comparison of overall effects on serum metabolic traits between lycopene intervention arms *vs*. placebo models. Estimates of the standard deviation (SD) difference in metabolic trait concentration between lycopene dietary advice and placebo arms at follow‐up (*x*‐axis) plotted against the SD difference in metabolic trait concentration in the lycopene supplement arm *vs*. placebo (*y*‐axis). (*b*) Comparison of overall effects on serum metabolic traits between green tea intervention arms *vs*. placebo models. Corresponding results for green tea. Each dot on plots A and B represents an individual metabolic trait. A linear fit of the overall correspondence summarises the similarity in magnitude between diet and supplement associations (solid lines). A slope of 1 with an intercept of 0 (dashed lines), with all dots sitting on that line (*R* ^2^ = 1), would indicate that diet and supplement estimates had the same magnitude and direction. Corresponding results for green tea. (*c*) SD follow−up metabolic trait concentration difference between lycopene diet or supplement *vs*. placebo. (*d*) SD follow−up metabolic trait concentration difference between green tea diet (drink) or supplement *vs*. placebo. Circles indicate *β*‐regression coefficients for the dietary intervention arms. Squares indicate *β*‐regression coefficients for the supplement arms. Closed symbols denote values that reached the threshold for multiple testing (*p* ≤ 0.004). Association magnitudes are in units of 1‐SD metabolic measure concentration. Horizontal bars represent 95% confidence intervals. Abbreviations: C, cholesterol; HDL, high‐density lipoprotein; IDL, intermediate‐density lipoprotein; LDL, low‐density lipoprotein; MUFA, monounsaturated fatty acids; PUFA, polyunsaturated fatty acids; VLDL, very‐low‐density lipoprotein.

There was a strong correlation between the effects of supplements compared to the effects of the dietary advice interventions on 6‐month metabolic measures (slope = 1.07 ± 0.06; *R* ^2^ = 0.65) (Fig. 3), which remained when regression models were adjusted for baseline serum metabolite levels (Supporting Information Fig. 1). Forest plots comparing the metabolic profile of supplemental and dietary advice interventions provide further confirmation that they have broadly comparable effects on the measured metabolic traits (Fig. 3). There were consistent decreases in the glycolysis‐related metabolites glucose, lactate and pyruvate after lycopene supplement and dietary advice interventions, as well as decreases in branched‐chain amino acid measures isoleucine, leucine and valine.

#### Lycopene effects

There was strong evidence of a reduction in valine in both supplement and dietary advice arms compared to placebo (*β* = −0.62; 95% CI = −1.03, −0.20; *p* = 0.004) and *β* = −0.65; 95% CI = −1.04, −0.26; *p* = 0.001 respectively, whereby *β* represents the standard deviation (SD) change in metabolic measures), as well as increased levels of acetate in the lycopene supplement group (*β* = 0.69; 95% CI = 0.24, 1.15; *p* = 0.003) (Table 1). There was some evidence that serum pyruvate and docosahexaenoic acid (DHA) were lower in the lycopene supplement group compared to placebo (*β* = −0.56; 95% CI = −0.95, −0.16; *p* = 0.006 and *β* = −0.50; 95% CI = −085, −0.14; *p* = 0.006 respectively), although effects did not reach our strict threshold for multiple testing (*p* = 0.004). Serum diacylglycerol levels were lower in the lycopene dietary advice group compared to placebo (*β* = −0.59; 95% CI = −1.01, −0.18; *p* = 0.006). See Supporting Information Table 4 for full results, including associations, expressed as magnitudes in absolute concentration units (e.g. mmol/L metabolite difference between diet or supplement *vs*. placebo).

**Table 1:** Linear regression results for metabolic traits that were found to be altered by supplement or dietary advice interventions (*n* = 128)

Metabolite	Intervention arm	Mean difference^1^	Lower CI	Upper CI	*p* value
*Lycopene*					
Valine	Supplement	−0.62	−1.03	−0.2	0.004^2^
	Dietary advice	−0.65	−1.04	−0.26	0.001^2^
Acetate	Supplement	0.69	0.24	1.15	0.003^2^
	Dietary advice	0.26	−0.08	0.59	0.129
Pyruvate	Supplement	−0.56	−0.95	−0.16	0.006
	Dietary advice	−0.30	−0.75	0.15	0.196
Diacylglycerol	Supplement	−0.47	−0.9	−0.03	0.036
	Dietary advice	−0.59	−1.01	−0.18	0.006
DHA	Supplement	−0.5	−0.85	−0.14	0.006
	Dietary advice	−0.15	−0.62	0.32	0.537
*Green tea*					
PUFA: FA	Supplement	0.66	0.27	1.05	0.001^2^
	Dietary advice	0.43	−0.01	0.86	0.057
Cholesterol esters in small HDL	Supplement	0.22	−0.24	0.67	0.347
	Dietary advice	0.62	0.19	1.04	0.005
Omega‐6: FA	Supplement	0.32	−0.12	0.76	0.148
	Dietary advice	0.22	−0.24	0.67	0.005
Glycine	Supplement	−0.32	−0.79	0.14	0.172
	Dietary advice	−0.58	−0.98	−0.18	0.005

1 Standardised mean difference (and 95% confidence interval [CI]) in metabolic trait concentration. Where there was evidence that one of the interventions altered follow‐up metabolic trait levels, results for the respective metabolic trait have been presented. For comparison, supplement and dietary advice results have been provided.

2 Metabolic measures that reached the principle component analysis based‐Bonferroni corrected threshold for multiple testing (*p* = 0.004).

Abbreviations: N, sample size; CI, confidence interval; DHA, docosahexaenoic acid; FA, fatty acid; HDL, high density lipoprotein; Omega‐6: FA, omega‐6 as a proportion of total FA; PUFA: FA, polyunsaturated fatty acids as a proportion of total FA. Omega‐6: FA and PUFA: FA are expressed as a % of total FA.

The trends for decreased valine, increased acetate and decreased pyruvate, were largely robust to adjustment for baseline metabolites (Supporting Information Table 5).

#### Correlation between metabolic profiles from green tea diet and supplement arms

Overall, the effects of green tea drinking and supplement interventions at follow‐up on serum metabolome were similar (slope = 0.57 ± 0.03, *R* ^2^ = 0.76) (Fig. 3).

#### Green tea effects

There was no strong evidence of an effect of green tea supplementation on serum metabolic profile. In the group advised to drink green tea, there was evidence of a reduction in the ratio of polyunsaturated fatty acids relative to total fatty acids (PUFA: FA) (*vs*. placebo), which survived correction for multiple testing (*β* = 0.66; 95% CI = 0.273, 1.049; *p* = 0.001) (Table 1). There was weaker evidence of a change in the proportions of omega‐6 and monounsaturated fatty acids relative to total fatty acids (*β* = 0.59; 95% CI = 0.19, 1.00; *p* = 0.005 and *β* = −0.58; 95% CI = −0.99, −0.17; *p* = 0.006, respectively). Post green‐tea drinking intervention levels of glycine were also lower compared to placebo (*β* = −0.58; 95% CI = −0.98, −0.18; *p* = 0.005) (full results in Supporting Information Table 6). The trends for increased PUFA: FA and reduced glycine, which were observed in the unadjusted green tea analyses, were not present in the adjusted analyses (Supporting Information Table 5).

### IV estimates

Results of the lycopene IV regression were broadly consistent with those of the intention to treat analysis (Supporting Information Table 7). There was an increase in acetate (*β* = 2.13; *p* = 0.006) and decreases in pyruvate (*β* = −1.90; *p* = 0.009), valine (*β* = −1.79; *p* = 0.023), diacylglycerol (*β* = −1.81; *p* = 0.026) and DHA (*p* = 0.097). Alanine was also lower (*β* = −1.55; *p* = 0.015). The IV analysis provided no strong evidence that green tea altered circulating metabolite levels (Supporting Information Table 7).

### Mendelian randomisation

#### Causal effects of metabolites on prostate cancer

Acetate, pyruvate, valine, diacylglycerol and DHA were taken forward for MR analysis because both the intention‐to‐treat and the IV analysis indicated they were modified in response to lycopene dietary intervention, although not all metabolic traits met our strict threshold for multiple testing. Glycine was also taken forward, since increased green tea intake was associated with altered glycine levels in the intention‐to‐treat analysis.

We identified 17 genetic variants associated with our metabolites of interest at genome‐wide significance (*p* < 5 × 10^−8^ for the allelic effect of each SNP on the exposure) in the MR Base GWAS database (http://www.mrbase.org/) (Table 2). The genetic variants comprised five sets of candidate genetic instruments corresponding to acetate, valine, pyruvate, DHA and glycine. Diacylglycerol was not available in the GWAS summary statistics from Kettunen *et al.,* 27 therefore this metabolite could not be instrumented.

**Table 2:** The association of individual SNPs with metabolites

Phenotype	Chromosome	Position	SNP	Effect allele	Other allele	EAF	Beta	SE	p‐value^1^	R^2^	F‐stat	N
Acetate	6	12,042,473	rs6933521	C	T	0.12	−0.092	0.016	8.10E‐09	0.0017	44.3	24,742
Pyruvate	2	27,730,940	rs1260326	C	T	0.64	−0.081	0.010	5.47E‐16	0.0030	59.4	22,529
Pyruvate	16	69,979,271	rs74249229	T	C	0.05	−0.153	0.023	2.13E‐11	0.0022		23,561
Valine	2	65,208,074	rs10211524	A	G	0.41	0.086	0.009	5.24E‐20	0.0036	79.0	24,898
Valine	4	89,206,230	rs9637599	C	A	0.47	0.114	0.009	1.67E‐35	0.0064		24,899
Valine	11	116,661,826	rs2072560	C	T	0.93	0.105	0.018	3.28E‐09	0.0014		24,895
Valine	17	7,063,667	rs7406661	C	T	0.24	0.079	0.013	5.35E‐10	0.0023		22,659
DHA	11	116,651,115	rs11604424	T	C	0.76	−0.083	0.018	7.84e‐09	0.0025	41.7	13,495
DHA	19	19,667,254	rs143988316	T	C	0.07	−0.150	0.026	1.10e‐09	0.0029		13,494
DHA	6	10,990,493	rs2281591	G	A	0.13	−0.108	0.003	3.66e‐09	0.0026		13,498
DHA	15	58,726,744	rs261334	C	G	0.77	−0.110	−0.020	1.44e‐13	0.0043		13,498
Glycine	2	210,439,980	rs147007805	A	T	0.07	−0.140	0.024	8.05E‐09	0.0027	444.6	18,732
Glycine	2	211,540,507	rs1047891	A	C	0.33	0.487	0.011	1.00E‐200	0.1055		18,730
Glycine	3	125,909,669	rs1992855	C	T	0.41	0.062	0.011	5.60E‐09	0.0019		18,733
Glycine	8	9,181,395	rs2169387	G	A	0.87	−0.130	0.016	1.31E‐16	0.0039		18,729
Glycine	9	5,934,989	rs13298772	C	T	0.05	0.273	0.023	3.74E‐33	0.0075		18,732
Glycine	16	81,065,282	rs10083777	T	C	0.17	−0.106	0.015	2.97E‐13	0.0032		18,732

1 Obtained from linear regression of exposure (metabolic trait) on instrument.

SNP, single nucleotide polymorphism; EAF, effect allele frequency; SE, standard error; N, sample size; DHA, docosahexaenoic acid.

The MR analysis provided some evidence that genetically raised pyruvate increased the odds of prostate cancer by 1.29 (95% CI: 1.03, 1.62; *p* = 0.027 (Bonferroni corrected *p* value = 0.05/4 = 0.0125)), using 2 SNPs (Table 3). There was no evidence that acetate, valine DHA, or glycine were causal in prostate cancer. Given evidence of a causal effect of pyruvate on prostate cancer risk, we further investigated functionality of the SNPs used to instrument pyruvate to identify potential pleiotropy. rs1260326 is located within GCKR, coding for the glucokinase regulatory protein which has a widespread effect on metabolite levels and is therefore likely to be highly pleiotropic (Supporting Information Fig. 2) rs74249229 is not consistently associated with other metabolites apart from pyruvate, alanine and lactate, which are all very closely related metabolites. However, as the variant is located within the PDPR (pyruvate dehydrogenase phosphatase regulatory) gene, this suggests the SNP primarily influences pyruvate, and therefore alanine and lactate through vertical (rather than horizontal) pleiotropy, suggesting that this SNP is likely to be a valid instrument for the MR analysis of pyruvate. Using only this SNP as an instrument, the causal effect of pyruvate on prostate cancer was found to be 1.31 (95% CI: 0.90, 1.93, *p* = 0.154), consistent with a positive causal effect of pyruvate on prostate cancer risk, albeit with a wider confidence interval.

**Table 3:** Causal effect estimates of metabolites on prostate cancer using individual‐level data from the PRACTICAL consortium

Metabolite	Number of SNPs	OR^†^	95% CI	P‐value
Acetate	1	0.89	0.63, 1.25	0.501
Pyruvate	2	1.29	1.03, 1.62	0.027
Valine	4	1.03	0.90, 1.18	0.647
DHA	4	0.97	0.85, 1.01	0.647
Glycine	6	0.99	0.92, 1.06	0.787

Mendelian randomisation estimates of odds ratios^†^ [OR] (and associated 95% confidence intervals [CI]) of prostate cancer risk per 1 standard deviation [SD] increase in genetically instrumented metabolite levels. Results obtained using the inverse‐variance weighted (IVW) method. DHA, docosahexaenoic acid.

## Discussion

In a sample of men with an elevated risk of prostate cancer, a 6‐month intervention to increase lycopene intake was found to modify circulating valine, acetate and pyruvate, compared to placebo, which were robust to adjustment for baseline metabolites. MR analysis provided some evidence that genetically predicted higher levels of pyruvate were associated with higher prostate cancer risk, supporting a causal role for this metabolite in prostate cancer aetiology. In this small/proof‐of‐concept trial, there was insufficient evidence to say whether supplementation with green tea affected the metabolome.

Metabolic reprogramming is a recognised hallmark of cancer.^43^ Elevated levels of important cellular metabolites (such as pyruvate) in the circulation may support and encourage the process of carcinogenesis, by fuelling metabolic pathways that are required to support cellular proliferation. Indeed, it has been demonstrated *in vitro* that cancer cells proliferate more rapidly in the presence of exogenous pyruvate through the fuelling of mitochondrial metabolism.^44^ Extracellular pyruvate may be a particularly important metabolite for prostate cancer cells because, unlike other cancer cell types, they do not rapidly metabolise glucose.^45^ This may mean their reliance on extracellular pyruvate as a source of acetyl‐CoA is crucial to tumour development. Consistent with this, the monocarboxylate transporter 2 (MCT2) which shows a high affinity for the transport of pyruvate^46^ is increased in expression in prostate cancer tissue.^47^, ^48^, ^49^

It was interesting to find a reduction in valine in the intention‐to‐treat analysis, even though we found no evidence of a causal link with PCa in the MR (using the four available SNPs). This is because there is an accumulating body of evidence to show that branched chain amino acids (BCAAs), including valine, leucine and isoleucine, may help support the high metabolic demands of tumour cells.^50^ For example, they can serve as indirect sources of nitrogen for nucleotide (and nonessential amino acid) biosynthesis and/or they can become further catabolised to yield acetyl‐CoA, which feeds into the tricarboxylic acids (TCA) cycle and can contribute to energy production.^50^ Further studies are needed to confirm our findings, and to establish whether a reduction in circulating BCAAs could have an impact on PCa risk, since few studies have examined the relation of BCAAs with PCa specifically.

Some studies suggest that certain tumours have acquired a dependency on acetate as a source of carbon to produce acetyl‐CoA.^51^, ^52^ In the current analysis, we observed an increase in acetate after lycopene dietary intervention however, which needs to be verified. If confirmed, this would suggest that any potential protective effects of lycopene intake on PCa are not acting through this metabolite.

### Study strengths and limitations

Our study has several strengths. Firstly, adherence to the ProDiet study was high.^16^ Thus, any differences in metabolites across intervention groups at follow‐up are most likely the result of the intervention itself, since all other measured variables were comparable at baseline. Secondly, we obtained metabolite measures across multiple metabolic pathways including glycolysis, the citric acid cycle and amino acid metabolism, facilitated through high‐throughput NMR, which is highly reproducible.^53^ Thirdly, by utilising summary statistics from a large prostate cancer consortium and effect estimates from a previous GWAS of metabolites, we were able to conduct a two‐sample MR to appraise the causal role of altered metabolites in prostate cancer risk.

Several limitations of our study warrant mention. A major limitation of our study was that the ProDiet RCT was originally designed to test the feasibility of a dietary intervention; it was not therefore powered to detect an effect of the intervention on metabolite levels. The small sample size precludes us from ruling out additional effects of lycopene and green tea dietary interventions on the serum metabolome. Furthermore, whilst the RCT design is designed to minimise residual confounding by distributing any known or unknown confounding factors across randomisation arms, there was evidence of a difference in some metabolite measures in the green tea intervention groups at baseline (attributable to chance). To address this, we conducted a sensitivity analyses, in which models were adjusted for baseline metabolite levels. The results were largely consistent.

An important aspect in any metabolomics study is the analytical reproducibility of the platform and protocols used. NMR has been shown to provide excellent reproducibility and quantitative accuracy.^53^ Moreover, the technique requires minimal sample preparation, decreasing the chances of analytical variability. The reproducibility of data generated from the NMR platform used in the current analysis has been assessed previously.^27^ The coefficients of variation ([CV] (in percent)) for selected metabolic measures are provided in Supporting Information Table 9. For pyruvate, valine, acetate, DHA and glycine (i.e. metabolites taken forwards to MR), the CVs were 4.7, 3.9, 5.5, 2.7 and 7.7% respectively.

A further potential issue, which is common feature of many ‘omics’ studies, is the assessment of many traits in a relatively small number of samples. Not considering the possible effect of multiple testing can greatly increase the probability of false positives (Type I errors), whilst an overly conservative approach may result in low statistical power to detect true positive signals (47). We used a multiple testing correction based on PCA to reduce false positives, as has been done previously (46).

We were unable to fully rule out the possibility of pleiotropy in the association between pyruvate and prostate cancer due to the limited number of genetic instruments currently available for pyruvate.

## Conclusions

In summary, our results suggest that in men with elevated prostate cancer risk, increasing dietary lycopene may result in changes in circulating levels of valine, acetate, pyruvate, diacylglycerol and docosahexaenoic acid. Our results provide some evidence that pyruvate may be causally related to prostate cancer risk and warrants investigation.

## Supporting information


**Appendix S1:** Supporting Information

**Appendix S2:** Supporting Information
