# Supplementary material for: Investigating the effects of lycopene and green tea on the metabolome of men at risk of prostate cancer: The ProDiet randomised controlled trial
Source: Int J Cancer. 2018 Dec 7;144(8):1918–28. doi: 10.1002/ijc.31929 (PMC6491994; doi:10.1002/ijc.31929)
Supplement: Supplementary file 1 — Appendix S1: Supporting Information [file IJC-144-1918-s001.docx]

**Supplementary Table 1:** Baseline demographic and clinical characteristics, stratified by lycopene intervention arms (n=128).

|  | Lycopene Treatment Groups | | |  |  |  |
| --- | --- | --- | --- | --- | --- | --- |
| Characteristics | Dietary Advice |  | Supplement |  | Placebo |  |
|  | (N = 43) |  | (N = 40) |  | (N = 45) |  |
|  | n | Mean (SD) or % | n | Mean (SD) or % | n | Mean (SD) or % |
| **Age** (years) | 43 | 64.5 (4.4) | 40 | 65.1 (5.7) | 45 | 64.3 (4.0) |
| **Body mass index** (Kg/m^2^) | 32 | 27.0 (3.7) | 31 | 26.0 (3.0) | 35 | 27.9 (3.9) |
| **PSA** (ng/ml) | 43 | 2.9 (1.1) | 40 | 3.5 (2.4) | 45 | 3.1 (1.2) |
| **Systolic blood pressure** (mmHg) | 40 | 144.8 (16.1) | 30 | 150.2 (15.6) | 33 | 141.3 (17.3) |
| **Total energy intake** (kcal/d) | 42 | 2247.0 (587.3) | 39 | 2271.0 (651.1) | 43 | 2431.8 (701.8) |
| **Occupational class** |  |  |  |  |  |  |
| Managerial | 22 | 51.2 | 19 | 47.5 | 25 | 56.8 |
| Intermediate | 14 | 32.6 | 10 | 25 | 12 | 27.3 |
| Manual/Routine | 7 | 16.3 | 11 | 27.5 | 7 | 15.9 |
| **Family history of prostate cancer** |  |  |  |  |  |  |
| Yes | 4 | 10.3 | 2 | 5.4 | 5 | 12.5 |
| No | 35 | 89.7 | 35 | 94.6 | 35 | 87.5 |
| **PSA** (ng/mL) | 43 | 2.9 (1.1) | 40 | 3.5 (2.4) | 45 | 3.1 (1.2) |
| **Diabetes** |  |  |  |  |  |  |
| Yes | 0 | 0 | 2 | 11.1 | 0 | 0 |
| No | 18 | 100 | 16 | 88.9 | 21 | 100 |
| **Smoking status** |  |  |  |  |  |  |
| Never | 20 | 47.6 | 25 | 64.1 | 20 | 46.5 |
| Past | 20 | 47.6 | 11 | 28.2 | 19 | 44.2 |
| Current | 2 | 4.8 | 3 | 7.7 | 4 | 9.3 |
| **Alcohol** (units/week) |  |  |  |  |  |  |
| <1 | 2 | 4.9 | 5 | 12.5 | 4 | 9 |
| 1 to 20 | 25 | 61 | 21 | 52.5 | 23 | 56.6 |
| >20 to 50 | 10 | 24.4 | 14 | 35 | 10 | 27.9 |
| ≥50 | 4 | 9.76 | 0 | 0 | 4 | 6.6 |

This table has been adapted from the main ProDiet manuscript (Lane, unpublished).

**Supplementary Table 2:** Baseline demographic and clinical characteristics, stratified by green tea intervention arms (n=128)

|  | Green tea Treatment Groups | |  |  |  |  |
| --- | --- | --- | --- | --- | --- | --- |
| Characteristics | Dietary Advice |  | Supplement |  | Placebo |  |
|  | (N = 44) |  | (N = 44) |  | (N = 40) |  |
|  | n | Mean (SD) or % | n | Mean (SD) or % | n | Mean (SD) or % |
| **Age** (years) | 44 | 64.2 (5.5) | 44 | 64.7 (4.2) | 40 | 65.1 (5.3) |
| **Body mass index** (Kg/m^2^) | 35 | 26.3 (3.4) | 29 | 27.8 (3.5) | 34 | 26.9 (3.7) |
| **PSA** (ng/ml) | 44 | 3.1 (1.6) | 44 | 3.1 (2.0) | 40 | 3.1 (1.4) |
| **Systolic blood pressure** (mmHg) | 41 | 146.5 (14.6) | 42 | 144.5 (16.3) | 38 | 145.0 (19.2) |
| **Total energy intake** (kcal/d) | 41 | 2492.6 (594.5) | 44 | 2252.1 (660.2) | 39 | 2210.8 (669.4) |
| **Occupational class** |  |  |  |  |  |  |
| Managerial | 26 | 59.1 | 19 | 43.2 | 21 | 53.9 |
| Intermediate | 12 | 27.3 | 15 | 34.1 | 9 | 23.1 |
| Manual/Routine | 6 | 13.6 | 10 | 22.7 | 9 | 23.1 |
| **Family history of prostate cancer** |  |  |  |  |  |  |
| Yes | 5 | 12.8 | 4 | 10 | 2 | 5.4 |
| No | 34 | 87.2 | 36 | 90 | 35 | 94.6 |
| **PSA** (ng/mL) | 44 | 3.1 (1.6) | 44 | 3.2 (2.0) | 40 | 3.1 (1.4) |
| **Diabetes** |  |  |  |  |  |  |
| Yes | 0 | 0 | 2 | 13.3 | 0 | 0 |
| No | 21 | 100 | 13 | 86.7 | 21 | 100 |
| **Smoking status** |  |  |  |  |  |  |
| Never | 21 | 51.2 | 24 | 54.5 | 20 | 52.4 |
| Past | 18 | 43.9 | 17 | 38.6 | 15 | 38.5 |
| Current | 2 | 4.9 | 3 | 6.8 | 4 | 10.3 |
| **Alcohol** (units/week) |  |  |  |  |  |  |
| <1 | 7 | 17.1 | 2 | 4.6 | 2 | 5.3 |
| 1 to 20 | 17 | 41.4 | 27 | 62.8 | 25 | 65.8 |
| >20 to 50 | 15 | 36.6 | 10 | 23.3 | 9 | 23.7 |
| ≥50 | 2 | 4.9 | 4 | 9.3 | 2 | 5.3 |

This table has been adapted from the main ProDiet manuscript (Lane, unpublish

**Supplementary Table 3**: Differences in metabolic trait concentration at baseline, stratified by lycopene and green tea intervention arms

| **Intervention arm** | **Metabolite** | **N** | **Mean*** | **SD** | **P-value** |
| --- | --- | --- | --- | --- | --- |
| Lycopene | | | | | |
| Dietary advice | Alb | 41 | 0.091 | 0.006 |  |
| Supplement |  | 38 | 0.090 | 0.006 |  |
| Placebo |  | 37 | 0.086 | 0.011 | 0.029 |
| Dietary advice | Ile | 41 | 0.062 | 0.016 |  |
| Supplement |  | 38 | 0.057 | 0.012 |  |
| Placebo |  | 37 | 0.065 | 0.016 | 0.079 |
| Dietary advice | Cit | 38 | 0.082 | 0.012 |  |
| Supplement |  | 37 | 0.088 | 0.019 |  |
| Placebo |  | 35 | 0.081 | 0.011 | 0.080 |
| Dietary advice | S_HDL_TG | 41 | 0.052 | 0.009 |  |
| Supplement |  | 38 | 0.049 | 0.010 |  |
| Placebo |  | 37 | 0.054 | 0.010 | 0.085 |
| Dietary advice | VLDL_D | 41 | 36.911 | 0.909 |  |
| Supplement |  | 38 | 36.710 | 0.981 |  |
| Placebo |  | 37 | 37.179 | 0.840 | 0.087 |
| Dietary advice | S_HDL_CE | 41 | 0.345 | 0.056 |  |
| Supplement |  | 38 | 0.350 | 0.044 |  |
| Placebo |  | 37 | 0.327 | 0.045 | 0.103 |
| Dietary advice | MUFA_FA | 41 | 25.930 | 3.313 |  |
| Supplement |  | 38 | 24.822 | 2.591 |  |
| Placebo |  | 37 | 26.163 | 2.857 | 0.111 |
| Dietary advice | S_HDL_C | 41 | 0.456 | 0.060 |  |
| Supplement |  | 38 | 0.461 | 0.047 |  |
| Placebo |  | 37 | 0.437 | 0.052 | 0.123 |
| Dietary advice | FALen | 41 | 17.637 | 0.355 |  |
| Supplement |  | 38 | 17.557 | 0.359 |  |
| Placebo |  | 37 | 17.726 | 0.352 | 0.125 |
| Dietary advice | Leu | 41 | 0.091 | 0.019 |  |
| Supplement |  | 38 | 0.085 | 0.012 |  |
| Placebo |  | 37 | 0.092 | 0.020 | 0.131 |
| Dietary advice | SFA_FA | 41 | 37.245 | 2.031 |  |
| Supplement |  | 38 | 38.143 | 2.185 |  |
| Placebo |  | 37 | 37.725 | 1.738 | 0.140 |
| Dietary advice | HDL2_C | 41 | 0.870 | 0.197 |  |
| Supplement |  | 38 | 0.931 | 0.221 |  |
| Placebo |  | 37 | 0.830 | 0.260 | 0.154 |
| Dietary advice | HDL_C | 41 | 1.336 | 0.211 |  |
| Supplement |  | 38 | 1.402 | 0.242 |  |
| Placebo |  | 37 | 1.292 | 0.286 | 0.156 |
|  |  |  |  |  |  |
|  |  |  |  |  |  |
| Dietary advice | S_VLDL_TG | 41 | 0.212 | 0.058 |  |
| Supplement |  | 38 | 0.198 | 0.056 |  |
| Placebo |  | 37 | 0.224 | 0.057 | 0.160 |
| Dietary advice | M_HDL_CE | 41 | 0.349 | 0.056 |  |
| Supplement |  | 38 | 0.360 | 0.054 |  |
| Placebo |  | 37 | 0.333 | 0.070 | 0.163 |
| Dietary advice | M_VLDL_TG | 41 | 0.267 | 0.105 |  |
| Supplement |  | 38 | 0.244 | 0.098 |  |
| Placebo |  | 37 | 0.288 | 0.101 | 0.167 |
| Dietary advice | M_HDL_C | 41 | 0.429 | 0.071 |  |
| Supplement |  | 38 | 0.442 | 0.069 |  |
| Placebo |  | 37 | 0.408 | 0.089 | 0.173 |
| Dietary advice | VLDL_TG | 41 | 0.771 | 0.292 |  |
| Supplement |  | 38 | 0.708 | 0.276 |  |
| Placebo |  | 37 | 0.832 | 0.284 | 0.174 |
| Dietary advice | XL_VLDL_TG | 41 | 0.035 | 0.025 |  |
| Supplement |  | 38 | 0.030 | 0.022 |  |
| Placebo |  | 37 | 0.041 | 0.023 | 0.176 |
| Dietary advice | L_VLDL_TG | 41 | 0.139 | 0.076 |  |
| Supplement |  | 38 | 0.124 | 0.070 |  |
| Placebo |  | 37 | 0.154 | 0.073 | 0.197 |
| Dietary advice | XL_VLDL_P | 41 | 0.000 | 0.000 |  |
| Supplement |  | 38 | 0.000 | 0.000 |  |
| Placebo |  | 37 | 0.000 | 0.000 | 0.197 |
| Dietary advice | XL_VLDL_L | 41 | 0.055 | 0.040 |  |
| Supplement |  | 38 | 0.049 | 0.037 |  |
| Placebo |  | 37 | 0.065 | 0.038 | 0.204 |
| Dietary advice | Tyr | 41 | 0.058 | 0.012 |  |
| Supplement |  | 38 | 0.055 | 0.011 |  |
| Placebo |  | 37 | 0.060 | 0.011 | 0.205 |
| Dietary advice | L_VLDL_P | 41 | 0.000 | 0.000 |  |
| Supplement |  | 38 | 0.000 | 0.000 |  |
| Placebo |  | 37 | 0.000 | 0.000 | 0.205 |
| Dietary advice | L_VLDL_L | 41 | 0.236 | 0.132 |  |
| Supplement |  | 38 | 0.211 | 0.125 |  |
| Placebo |  | 37 | 0.264 | 0.129 | 0.208 |
| Dietary advice | Val | 41 | 0.190 | 0.039 |  |
| Supplement |  | 38 | 0.179 | 0.028 |  |
| Placebo |  | 37 | 0.193 | 0.038 | 0.208 |
| Dietary advice | Serum_TG | 41 | 1.168 | 0.347 |  |
| Supplement |  | 38 | 1.098 | 0.334 |  |
| Placebo |  | 37 | 1.238 | 0.346 | 0.213 |
| Dietary advice | Phe | 41 | 0.081 | 0.012 |  |
| Supplement |  | 38 | 0.077 | 0.010 |  |
| Placebo |  | 37 | 0.081 | 0.013 | 0.213 |
| Dietary advice | M_HDL_FC | 41 | 0.079 | 0.015 |  |
| Supplement |  | 38 | 0.082 | 0.015 |  |
| Placebo |  | 37 | 0.075 | 0.019 | 0.223 |
| Dietary advice | L_VLDL_FC | 41 | 0.023 | 0.016 |  |
| Supplement |  | 38 | 0.021 | 0.015 |  |
| Placebo |  | 37 | 0.027 | 0.016 | 0.223 |
| Dietary advice | XL_VLDL_CE | 41 | 0.007 | 0.005 |  |
| Supplement |  | 38 | 0.006 | 0.005 |  |
| Placebo |  | 37 | 0.008 | 0.005 | 0.224 |
| Dietary advice | L_VLDL_C | 41 | 0.055 | 0.033 |  |
| Supplement |  | 38 | 0.050 | 0.032 |  |
| Placebo |  | 37 | 0.062 | 0.032 | 0.224 |
| Dietary advice | L_VLDL_PL | 41 | 0.043 | 0.024 |  |
| Supplement |  | 38 | 0.038 | 0.023 |  |
| Placebo |  | 37 | 0.048 | 0.024 | 0.226 |
| Dietary advice | M_VLDL_P | 41 | 0.000 | 0.000 |  |
| Supplement |  | 38 | 0.000 | 0.000 |  |
| Placebo |  | 37 | 0.000 | 0.000 | 0.228 |
| Dietary advice | M_VLDL_FC | 41 | 0.057 | 0.025 |  |
| Supplement |  | 38 | 0.052 | 0.024 |  |
| Placebo |  | 37 | 0.062 | 0.024 | 0.231 |
| Dietary advice | XXL_VLDL_FC | 41 | 0.002 | 0.001 |  |
| Supplement |  | 38 | 0.002 | 0.001 |  |
| Placebo |  | 37 | 0.002 | 0.001 | 0.233 |
| Dietary advice | L_VLDL_CE | 41 | 0.032 | 0.017 |  |
| Supplement |  | 38 | 0.029 | 0.017 |  |
| Placebo |  | 37 | 0.036 | 0.017 | 0.233 |
| Dietary advice | ApoA1 | 41 | 1.460 | 0.129 |  |
| Supplement |  | 38 | 1.495 | 0.155 |  |
| Placebo |  | 37 | 1.433 | 0.188 | 0.233 |
| Dietary advice | L_HDL_PL | 41 | 0.338 | 0.092 |  |
| Supplement |  | 38 | 0.364 | 0.102 |  |
| Placebo |  | 37 | 0.324 | 0.117 | 0.235 |
| Dietary advice | M_VLDL_L | 41 | 0.508 | 0.199 |  |
| Supplement |  | 38 | 0.470 | 0.187 |  |
| Placebo |  | 37 | 0.546 | 0.191 | 0.242 |
| Dietary advice | XL_VLDL_C | 41 | 0.012 | 0.009 |  |
| Supplement |  | 38 | 0.011 | 0.009 |  |
| Placebo |  | 37 | 0.014 | 0.009 | 0.249 |
| Dietary advice | XS_VLDL_TG | 41 | 0.101 | 0.022 |  |
| Supplement |  | 38 | 0.096 | 0.022 |  |
| Placebo |  | 37 | 0.105 | 0.022 | 0.250 |
| Dietary advice | L_HDL_P | 41 | 0.000 | 0.000 |  |
| Supplement |  | 38 | 0.000 | 0.000 |  |
| Placebo |  | 37 | 0.000 | 0.000 | 0.255 |
| Dietary advice | FAw6_FA | 41 | 32.601 | 3.171 |  |
| Supplement |  | 38 | 33.218 | 2.997 |  |
| Placebo |  | 37 | 32.076 | 2.720 | 0.255 |
| Dietary advice | L_HDL_L | 41 | 0.665 | 0.202 |  |
| Supplement |  | 38 | 0.727 | 0.224 |  |
| Placebo |  | 37 | 0.644 | 0.250 | 0.256 |
| Dietary advice | L_HDL_CE | 41 | 0.238 | 0.081 |  |
| Supplement |  | 38 | 0.264 | 0.089 |  |
| Placebo |  | 37 | 0.232 | 0.097 | 0.264 |
| Dietary advice | M_VLDL_PL | 41 | 0.103 | 0.039 |  |
| Supplement |  | 38 | 0.095 | 0.036 |  |
| Placebo |  | 37 | 0.110 | 0.037 | 0.265 |
| Dietary advice | L_HDL_C | 41 | 0.306 | 0.107 |  |
| Supplement |  | 38 | 0.339 | 0.117 |  |
| Placebo |  | 37 | 0.297 | 0.128 | 0.268 |
| Dietary advice | M_HDL_L | 41 | 0.881 | 0.129 |  |
| Supplement |  | 38 | 0.902 | 0.128 |  |
| Placebo |  | 37 | 0.849 | 0.160 | 0.268 |
| Dietary advice | XL_VLDL_PL | 41 | 0.009 | 0.007 |  |
| Supplement |  | 38 | 0.008 | 0.006 |  |
| Placebo |  | 37 | 0.010 | 0.007 | 0.269 |
| Dietary advice | L_HDL_FC | 41 | 0.067 | 0.026 |  |
| Supplement |  | 38 | 0.075 | 0.028 |  |
| Placebo |  | 37 | 0.065 | 0.031 | 0.284 |
| Dietary advice | XL_VLDL_FC | 41 | 0.005 | 0.004 |  |
| Supplement |  | 38 | 0.005 | 0.004 |  |
| Placebo |  | 37 | 0.006 | 0.004 | 0.284 |
| Dietary advice | FAw3_FA | 41 | 4.224 | 1.521 |  |
| Supplement |  | 38 | 3.818 | 0.716 |  |
| Placebo |  | 37 | 4.036 | 0.983 | 0.288 |
| Dietary advice | M_HDL_P | 41 | 0.000 | 0.000 |  |
| Supplement |  | 38 | 0.000 | 0.000 |  |
| Placebo |  | 37 | 0.000 | 0.000 | 0.288 |
| Dietary advice | XXL_VLDL_TG | 41 | 0.017 | 0.011 |  |
| Supplement |  | 38 | 0.015 | 0.011 |  |
| Placebo |  | 37 | 0.019 | 0.010 | 0.290 |
| Dietary advice | XXL_VLDL_P | 41 | 0.000 | 0.000 |  |
| Supplement |  | 38 | 0.000 | 0.000 |  |
| Placebo |  | 37 | 0.000 | 0.000 | 0.308 |
| Dietary advice | XXL_VLDL_L | 41 | 0.024 | 0.016 |  |
| Supplement |  | 38 | 0.022 | 0.016 |  |
| Placebo |  | 37 | 0.027 | 0.015 | 0.309 |
| Dietary advice | XXL_VLDL_PL | 41 | 0.003 | 0.002 |  |
| Supplement |  | 38 | 0.002 | 0.002 |  |
| Placebo |  | 37 | 0.003 | 0.002 | 0.313 |
| Dietary advice | XL_HDL_PL | 41 | 0.160 | 0.073 |  |
| Supplement |  | 38 | 0.181 | 0.077 |  |
| Placebo |  | 37 | 0.157 | 0.077 | 0.313 |
| Dietary advice | HDL3_C | 41 | 0.466 | 0.023 |  |
| Supplement |  | 38 | 0.471 | 0.023 |  |
| Placebo |  | 37 | 0.462 | 0.030 | 0.321 |
| Dietary advice | HDL_D | 41 | 9.870 | 0.164 |  |
| Supplement |  | 38 | 9.913 | 0.177 |  |
| Placebo |  | 37 | 9.856 | 0.177 | 0.325 |
| Dietary advice | Gly | 41 | 0.289 | 0.057 |  |
| Supplement |  | 38 | 0.276 | 0.033 |  |
| Placebo |  | 37 | 0.274 | 0.048 | 0.331 |
| Dietary advice | dha | 41 | 0.136 | 0.076 |  |
| Supplement |  | 38 | 0.118 | 0.033 |  |
| Placebo |  | 37 | 0.126 | 0.046 | 0.333 |
| Dietary advice | HDL_TG | 41 | 0.132 | 0.021 |  |
| Supplement |  | 38 | 0.131 | 0.022 |  |
| Placebo |  | 37 | 0.138 | 0.022 | 0.337 |
| Dietary advice | M_HDL_PL | 41 | 0.406 | 0.057 |  |
| Supplement |  | 38 | 0.415 | 0.059 |  |
| Placebo |  | 37 | 0.394 | 0.070 | 0.342 |
| Dietary advice | FAw3 | 41 | 0.422 | 0.200 |  |
| Supplement |  | 38 | 0.373 | 0.096 |  |
| Placebo |  | 37 | 0.395 | 0.120 | 0.343 |
| Dietary advice | DHA_FA | 41 | 1.354 | 0.594 |  |
| Supplement |  | 38 | 1.211 | 0.286 |  |
| Placebo |  | 37 | 1.276 | 0.353 | 0.351 |
| Dietary advice | S_VLDL_P | 41 | 0.000 | 0.000 |  |
| Supplement |  | 38 | 0.000 | 0.000 |  |
| Placebo |  | 37 | 0.000 | 0.000 | 0.374 |
| Dietary advice | XXL_VLDL_C | 41 | 0.004 | 0.003 |  |
| Supplement |  | 38 | 0.004 | 0.003 |  |
| Placebo |  | 37 | 0.005 | 0.003 | 0.385 |
| Dietary advice | PUFA_FA | 41 | 36.825 | 3.433 |  |
| Supplement |  | 38 | 37.035 | 2.947 |  |
| Placebo |  | 37 | 36.112 | 2.666 | 0.391 |
| Dietary advice | XL_HDL_FC | 41 | 0.033 | 0.016 |  |
| Supplement |  | 38 | 0.038 | 0.017 |  |
| Placebo |  | 37 | 0.034 | 0.016 | 0.394 |
| Dietary advice | Gln | 41 | 0.559 | 0.078 |  |
| Supplement |  | 38 | 0.537 | 0.058 |  |
| Placebo |  | 37 | 0.540 | 0.091 | 0.403 |
| Dietary advice | XL_HDL_L | 41 | 0.318 | 0.127 |  |
| Supplement |  | 38 | 0.354 | 0.138 |  |
| Placebo |  | 37 | 0.320 | 0.133 | 0.405 |
| Dietary advice | XL_HDL_P | 41 | 0.000 | 0.000 |  |
| Supplement |  | 38 | 0.000 | 0.000 |  |
| Placebo |  | 37 | 0.000 | 0.000 | 0.405 |
| Dietary advice | S_VLDL_PL | 41 | 0.128 | 0.027 |  |
| Supplement |  | 38 | 0.123 | 0.027 |  |
| Placebo |  | 37 | 0.131 | 0.026 | 0.411 |
| Dietary advice | S_VLDL_L | 41 | 0.528 | 0.135 |  |
| Supplement |  | 38 | 0.503 | 0.130 |  |
| Placebo |  | 37 | 0.542 | 0.131 | 0.432 |
| Dietary advice | M_VLDL_C | 41 | 0.138 | 0.057 |  |
| Supplement |  | 38 | 0.131 | 0.055 |  |
| Placebo |  | 37 | 0.148 | 0.055 | 0.436 |
| Dietary advice | XL_HDL_TG | 41 | 0.013 | 0.005 |  |
| Supplement |  | 38 | 0.013 | 0.005 |  |
| Placebo |  | 37 | 0.014 | 0.005 | 0.443 |
| Dietary advice | dag | 39 | 0.017 | 0.011 |  |
| Supplement |  | 37 | 0.018 | 0.016 |  |
| Placebo |  | 33 | 0.015 | 0.010 | 0.460 |
| Dietary advice | L_HDL_TG | 41 | 0.022 | 0.007 |  |
| Supplement |  | 38 | 0.024 | 0.008 |  |
| Placebo |  | 37 | 0.023 | 0.007 | 0.472 |
| Dietary advice | S_VLDL_FC | 41 | 0.074 | 0.019 |  |
| Supplement |  | 38 | 0.070 | 0.018 |  |
| Placebo |  | 37 | 0.075 | 0.018 | 0.495 |
| Dietary advice | LA_FA0 | 41 | 25.845 | 3.697 |  |
| Supplement |  | 38 | 26.225 | 3.112 |  |
| Placebo |  | 37 | 25.344 | 2.827 | 0.502 |
| Dietary advice | XL_HDL_C | 41 | 0.145 | 0.055 |  |
| Supplement |  | 38 | 0.160 | 0.060 |  |
| Placebo |  | 37 | 0.149 | 0.057 | 0.507 |
| Dietary advice | S_HDL_L | 41 | 1.110 | 0.103 |  |
| Supplement |  | 38 | 1.113 | 0.089 |  |
| Placebo |  | 37 | 1.089 | 0.106 | 0.508 |
| Dietary advice | mufa | 41 | 2.560 | 0.644 |  |
| Supplement |  | 38 | 2.436 | 0.555 |  |
| Placebo |  | 37 | 2.588 | 0.641 | 0.518 |
| Dietary advice | His | 41 | 0.071 | 0.008 |  |
| Supplement |  | 38 | 0.070 | 0.009 |  |
| Placebo |  | 37 | 0.072 | 0.010 | 0.522 |
| Dietary advice | XXL_VLDL_CE | 41 | 0.002 | 0.002 |  |
| Supplement |  | 38 | 0.002 | 0.002 |  |
| Placebo |  | 37 | 0.003 | 0.002 | 0.524 |
| Dietary advice | IDL_TG0 | 41 | 0.102 | 0.020 |  |
| Supplement |  | 38 | 0.099 | 0.019 |  |
| Placebo |  | 37 | 0.104 | 0.021 | 0.541 |
| Dietary advice | XL_HDL_CE | 41 | 0.112 | 0.039 |  |
| Supplement |  | 38 | 0.122 | 0.043 |  |
| Placebo |  | 37 | 0.115 | 0.041 | 0.560 |
| Dietary advice | M_HDL_TG | 41 | 0.046 | 0.007 |  |
| Supplement |  | 38 | 0.045 | 0.008 |  |
| Placebo |  | 37 | 0.047 | 0.008 | 0.561 |
| Dietary advice | EstC | 41 | 2.771 | 0.613 |  |
| Supplement |  | 38 | 2.836 | 0.660 |  |
| Placebo |  | 37 | 2.676 | 0.671 | 0.562 |
| Dietary advice | S_HDL_P | 41 | 0.000 | 0.000 |  |
| Supplement |  | 38 | 0.000 | 0.000 |  |
| Placebo |  | 37 | 0.000 | 0.000 | 0.570 |
| Dietary advice | Gp | 41 | 1.345 | 0.156 |  |
| Supplement |  | 38 | 1.308 | 0.124 |  |
| Placebo |  | 37 | 1.330 | 0.189 | 0.583 |
| Dietary advice | Pyr | 41 | 0.119 | 0.043 |  |
| Supplement |  | 38 | 0.110 | 0.037 |  |
| Placebo |  | 37 | 0.114 | 0.037 | 0.603 |
| Dietary advice | IDL_FC | 41 | 0.163 | 0.049 |  |
| Supplement |  | 38 | 0.166 | 0.053 |  |
| Placebo |  | 37 | 0.155 | 0.052 | 0.606 |
| Dietary advice | L_LDL_FC | 41 | 0.204 | 0.057 |  |
| Supplement |  | 38 | 0.207 | 0.060 |  |
| Placebo |  | 37 | 0.194 | 0.059 | 0.606 |
| Dietary advice | Serum_C | 41 | 3.854 | 0.845 |  |
| Supplement |  | 38 | 3.927 | 0.918 |  |
| Placebo |  | 37 | 3.724 | 0.951 | 0.616 |
| Dietary advice | Crea | 41 | 0.064 | 0.012 |  |
| Supplement |  | 38 | 0.063 | 0.010 |  |
| Placebo |  | 37 | 0.065 | 0.011 | 0.636 |
| Dietary advice | S_LDL_FC | 41 | 0.075 | 0.018 |  |
| Supplement |  | 38 | 0.076 | 0.017 |  |
| Placebo |  | 37 | 0.072 | 0.019 | 0.648 |
| Dietary advice | M_VLDL_CE | 41 | 0.082 | 0.033 |  |
| Supplement |  | 38 | 0.079 | 0.033 |  |
| Placebo |  | 37 | 0.086 | 0.032 | 0.651 |
| Dietary advice | S_LDL_C | 41 | 0.256 | 0.093 |  |
| Supplement |  | 38 | 0.257 | 0.093 |  |
| Placebo |  | 37 | 0.240 | 0.094 | 0.656 |
| Dietary advice | S_LDL_CE | 41 | 0.180 | 0.075 |  |
| Supplement |  | 38 | 0.181 | 0.076 |  |
| Placebo |  | 37 | 0.167 | 0.075 | 0.657 |
| Dietary advice | bOHBut | 41 | 0.108 | 0.040 |  |
| Supplement |  | 38 | 0.117 | 0.064 |  |
| Placebo |  | 37 | 0.109 | 0.045 | 0.658 |
| Dietary advice | Lac | 41 | 1.969 | 0.629 |  |
| Supplement |  | 38 | 1.863 | 0.463 |  |
| Placebo |  | 37 | 1.944 | 0.496 | 0.659 |
| Dietary advice | M_LDL_FC | 41 | 0.122 | 0.028 |  |
| Supplement |  | 38 | 0.123 | 0.028 |  |
| Placebo |  | 37 | 0.118 | 0.029 | 0.667 |
| Dietary advice | IDL_C | 41 | 0.553 | 0.173 |  |
| Supplement |  | 38 | 0.567 | 0.187 |  |
| Placebo |  | 37 | 0.529 | 0.186 | 0.669 |
| Dietary advice | L_LDL_PL | 41 | 0.275 | 0.066 |  |
| Supplement |  | 38 | 0.278 | 0.070 |  |
| Placebo |  | 37 | 0.264 | 0.069 | 0.676 |
| Dietary advice | LDL_C | 41 | 1.378 | 0.478 |  |
| Supplement |  | 38 | 1.390 | 0.489 |  |
| Placebo |  | 37 | 1.300 | 0.490 | 0.685 |
| Dietary advice | IDL_PL | 41 | 0.252 | 0.065 |  |
| Supplement |  | 38 | 0.255 | 0.070 |  |
| Placebo |  | 37 | 0.242 | 0.067 | 0.685 |
| Dietary advice | M_LDL_C | 41 | 0.415 | 0.151 |  |
| Supplement |  | 38 | 0.417 | 0.152 |  |
| Placebo |  | 37 | 0.390 | 0.152 | 0.687 |
| Dietary advice | Glc | 41 | 4.734 | 1.533 |  |
| Supplement |  | 38 | 4.528 | 1.212 |  |
| Placebo |  | 37 | 4.802 | 1.531 | 0.689 |
| Dietary advice | S_LDL_PL | 41 | 0.130 | 0.026 |  |
| Supplement |  | 38 | 0.131 | 0.026 |  |
| Placebo |  | 37 | 0.126 | 0.028 | 0.689 |
| Dietary advice | M_LDL_CE | 41 | 0.293 | 0.123 |  |
| Supplement |  | 38 | 0.294 | 0.124 |  |
| Placebo |  | 37 | 0.272 | 0.123 | 0.691 |
| Dietary advice | L_LDL_C | 41 | 0.707 | 0.235 |  |
| Supplement |  | 38 | 0.715 | 0.246 |  |
| Placebo |  | 37 | 0.671 | 0.244 | 0.693 |
| Dietary advice | IDL_CE | 41 | 0.391 | 0.125 |  |
| Supplement |  | 38 | 0.400 | 0.134 |  |
| Placebo |  | 37 | 0.375 | 0.134 | 0.695 |
| Dietary advice | pc | 41 | 1.728 | 0.243 |  |
| Supplement |  | 38 | 1.748 | 0.279 |  |
| Placebo |  | 37 | 1.691 | 0.355 | 0.700 |
| Dietary advice | TotCho | 41 | 2.009 | 0.290 |  |
| Supplement |  | 38 | 2.040 | 0.313 |  |
| Placebo |  | 37 | 1.975 | 0.383 | 0.702 |
| Dietary advice | S_LDL_L | 41 | 0.414 | 0.123 |  |
| Supplement |  | 38 | 0.417 | 0.122 |  |
| Placebo |  | 37 | 0.395 | 0.126 | 0.706 |
| Dietary advice | L_LDL_CE | 41 | 0.504 | 0.179 |  |
| Supplement |  | 38 | 0.508 | 0.186 |  |
| Placebo |  | 37 | 0.476 | 0.185 | 0.720 |
| Dietary advice | S_LDL_TG | 41 | 0.029 | 0.007 |  |
| Supplement |  | 38 | 0.028 | 0.007 |  |
| Placebo |  | 37 | 0.029 | 0.007 | 0.723 |
| Dietary advice | S_LDL_P0 | 41 | 0.000 | 0.000 |  |
| Supplement |  | 38 | 0.000 | 0.000 |  |
| Placebo |  | 37 | 0.000 | 0.000 | 0.724 |
| Dietary advice | L_LDL_L | 41 | 1.070 | 0.315 |  |
| Supplement |  | 38 | 1.080 | 0.328 |  |
| Placebo |  | 37 | 1.024 | 0.328 | 0.728 |
| Dietary advice | M_LDL_L | 41 | 0.632 | 0.196 |  |
| Supplement |  | 38 | 0.635 | 0.196 |  |
| Placebo |  | 37 | 0.603 | 0.199 | 0.730 |
| Dietary advice | UnSat | 41 | 1.223 | 0.076 |  |
| Supplement |  | 38 | 1.217 | 0.057 |  |
| Placebo |  | 37 | 1.211 | 0.059 | 0.732 |
| Dietary advice | M_LDL_P | 41 | 0.000 | 0.000 |  |
| Supplement |  | 38 | 0.000 | 0.000 |  |
| Placebo |  | 37 | 0.000 | 0.000 | 0.745 |
| Dietary advice | IDL_L | 41 | 0.907 | 0.252 |  |
| Supplement |  | 38 | 0.921 | 0.270 |  |
| Placebo |  | 37 | 0.876 | 0.267 | 0.747 |
| Dietary advice | L_LDL_P | 41 | 0.000 | 0.000 |  |
| Supplement |  | 38 | 0.000 | 0.000 |  |
| Placebo |  | 37 | 0.000 | 0.000 | 0.750 |
| Dietary advice | VLDL_C | 41 | 0.586 | 0.196 |  |
| Supplement |  | 38 | 0.569 | 0.194 |  |
| Placebo |  | 37 | 0.603 | 0.194 | 0.754 |
| Dietary advice | FreeC | 41 | 1.082 | 0.243 |  |
| Supplement |  | 38 | 1.091 | 0.265 |  |
| Placebo |  | 37 | 1.048 | 0.290 | 0.757 |
| Dietary advice | M_LDL_PL | 41 | 0.173 | 0.039 |  |
| Supplement |  | 38 | 0.174 | 0.039 |  |
| Placebo |  | 37 | 0.168 | 0.039 | 0.760 |
| Dietary advice | Glol | 41 | 0.047 | 0.014 |  |
| Supplement |  | 38 | 0.049 | 0.017 |  |
| Placebo |  | 37 | 0.046 | 0.017 | 0.763 |
| Dietary advice | LDL_D | 41 | 23.492 | 0.100 |  |
| Supplement |  | 38 | 23.491 | 0.107 |  |
| Placebo |  | 37 | 23.506 | 0.092 | 0.768 |
| Dietary advice | FAw6 | 41 | 3.179 | 0.522 |  |
| Supplement |  | 38 | 3.233 | 0.562 |  |
| Placebo |  | 37 | 3.138 | 0.625 | 0.770 |
| Dietary advice | IDL_P | 41 | 0.000 | 0.000 |  |
| Supplement |  | 38 | 0.000 | 0.000 |  |
| Placebo |  | 37 | 0.000 | 0.000 | 0.784 |
| Dietary advice | Ace | 41 | 0.041 | 0.009 |  |
| Supplement |  | 38 | 0.040 | 0.009 |  |
| Placebo |  | 37 | 0.040 | 0.013 | 0.816 |
| Dietary advice | XS_VLDL_PL | 41 | 0.133 | 0.036 |  |
| Supplement |  | 38 | 0.133 | 0.038 |  |
| Placebo |  | 37 | 0.128 | 0.035 | 0.820 |
| Dietary advice | cla | 41 | 0.022 | 0.015 |  |
| Supplement |  | 37 | 0.024 | 0.013 |  |
| Placebo |  | 37 | 0.024 | 0.017 | 0.833 |
| Dietary advice | la | 41 | 2.516 | 0.461 |  |
| Supplement |  | 38 | 2.554 | 0.486 |  |
| Placebo |  | 37 | 2.487 | 0.556 | 0.845 |
| Dietary advice | LDL_TG | 41 | 0.162 | 0.034 |  |
| Supplement |  | 38 | 0.159 | 0.033 |  |
| Placebo |  | 37 | 0.164 | 0.036 | 0.845 |
| Dietary advice | Ala | 41 | 0.488 | 0.081 |  |
| Supplement |  | 38 | 0.479 | 0.075 |  |
| Placebo |  | 37 | 0.480 | 0.084 | 0.853 |
| Dietary advice | TotPG | 41 | 1.595 | 0.254 |  |
| Supplement |  | 38 | 1.612 | 0.269 |  |
| Placebo |  | 37 | 1.576 | 0.323 | 0.854 |
| Dietary advice | L_LDL_TG | 41 | 0.089 | 0.018 |  |
| Supplement |  | 38 | 0.087 | 0.018 |  |
| Placebo |  | 37 | 0.090 | 0.019 | 0.859 |
| Dietary advice | pufa | 41 | 3.601 | 0.643 |  |
| Supplement |  | 38 | 3.606 | 0.623 |  |
| Placebo |  | 37 | 3.533 | 0.684 | 0.861 |
| Dietary advice | XS_VLDL_FC | 41 | 0.068 | 0.017 |  |
| Supplement |  | 38 | 0.068 | 0.017 |  |
| Placebo |  | 37 | 0.066 | 0.017 | 0.870 |
| Dietary advice | sm | 41 | 0.413 | 0.078 |  |
| Supplement |  | 38 | 0.410 | 0.080 |  |
| Placebo |  | 37 | 0.403 | 0.088 | 0.877 |
| Dietary advice | CLA_FA | 41 | 0.223 | 0.132 |  |
| Supplement |  | 37 | 0.232 | 0.114 |  |
| Placebo |  | 37 | 0.238 | 0.141 | 0.880 |
| Dietary advice | S_VLDL_C | 41 | 0.187 | 0.057 |  |
| Supplement |  | 38 | 0.182 | 0.056 |  |
| Placebo |  | 37 | 0.188 | 0.054 | 0.892 |
| Dietary advice | M_LDL_TG | 41 | 0.045 | 0.009 |  |
| Supplement |  | 38 | 0.044 | 0.009 |  |
| Placebo |  | 37 | 0.045 | 0.010 | 0.900 |
| Dietary advice | XS_VLDL_CE | 41 | 0.121 | 0.038 |  |
| Supplement |  | 38 | 0.123 | 0.041 |  |
| Placebo |  | 37 | 0.119 | 0.038 | 0.902 |
| Dietary advice | XS_VLDL_C | 41 | 0.189 | 0.055 |  |
| Supplement |  | 38 | 0.191 | 0.057 |  |
| Placebo |  | 37 | 0.185 | 0.054 | 0.909 |
| Dietary advice | S_HDL_FC | 41 | 0.111 | 0.012 |  |
| Supplement |  | 38 | 0.111 | 0.012 |  |
| Placebo |  | 37 | 0.110 | 0.013 | 0.922 |
| Dietary advice | sfa | 41 | 3.654 | 0.671 |  |
| Supplement |  | 38 | 3.711 | 0.580 |  |
| Placebo |  | 37 | 3.699 | 0.753 | 0.925 |
| Dietary advice | S_HDL_PL | 41 | 0.603 | 0.068 |  |
| Supplement |  | 38 | 0.603 | 0.063 |  |
| Placebo |  | 37 | 0.598 | 0.069 | 0.930 |
| Dietary advice | XS_VLDL_L | 41 | 0.424 | 0.106 |  |
| Supplement |  | 38 | 0.420 | 0.108 |  |
| Placebo |  | 37 | 0.419 | 0.104 | 0.974 |
| Dietary advice | XS_VLDL_P | 41 | 0.000 | 0.000 |  |
| Supplement |  | 38 | 0.000 | 0.000 |  |
| Placebo |  | 37 | 0.000 | 0.000 | 0.974 |
| Dietary advice | ApoB | 41 | 0.773 | 0.174 |  |
| Supplement |  | 38 | 0.769 | 0.177 |  |
| Placebo |  | 37 | 0.765 | 0.176 | 0.981 |
| Dietary advice | S_VLDL_CE | 41 | 0.114 | 0.040 |  |
| Supplement |  | 38 | 0.112 | 0.041 |  |
| Placebo |  | 37 | 0.113 | 0.037 | 0.982 |
| Dietary advice | TotFA | 41 | 9.815 | 1.745 |  |
| Supplement |  | 38 | 9.753 | 1.598 |  |
| Placebo |  | 37 | 9.820 | 1.948 | 0.983 |
| Dietary advice | Remnant_C | 41 | 1.140 | 0.348 |  |
| Supplement |  | 38 | 1.135 | 0.357 |  |
| Placebo |  | 37 | 1.132 | 0.354 | 0.996 |
| Green tea | | | | | |
| Dietary advice | Gly | 40 | 0.26 | 0.04 |  |
| Supplement |  | 40 | 0.28 | 0.05 |  |
| Placebo |  | 36 | 0.30 | 0.05 | 0.004 |
| Dietary advice | PUFA_FA | 40 | 37.71 | 2.70 |  |
| Supplement |  | 40 | 36.70 | 3.27 |  |
| Placebo |  | 36 | 35.47 | 2.78 | 0.005 |
| Dietary advice | Phe | 40 | 0.08 | 0.01 |  |
| Supplement |  | 40 | 0.08 | 0.01 |  |
| Placebo |  | 36 | 0.08 | 0.01 | 0.012 |
|  |  |  |  |  |  |
|  |  |  |  |  |  |
| Dietary advice | Gp | 40 | 1.29 | 0.12 |  |
| Supplement |  | 40 | 1.32 | 0.17 |  |
| Placebo |  | 36 | 1.38 | 0.16 | 0.025 |
| Dietary advice | LA_FA | 40 | 26.90 | 3.17 |  |
| Supplement |  | 40 | 25.40 | 3.41 |  |
| Placebo |  | 36 | 25.06 | 2.86 | 0.027 |
| Dietary advice | FAw6_FA | 40 | 33.56 | 2.68 |  |
| Supplement |  | 40 | 32.50 | 3.21 |  |
| Placebo |  | 36 | 31.75 | 2.84 | 0.028 |
| Dietary advice | Ala | 40 | 0.46 | 0.07 |  |
| Supplement |  | 40 | 0.50 | 0.08 |  |
| Placebo |  | 36 | 0.48 | 0.08 | 0.029 |
| Dietary advice | MUFA_FA | 40 | 24.92 | 2.76 |  |
| Supplement |  | 40 | 25.47 | 2.85 |  |
| Placebo |  | 36 | 26.63 | 3.15 | 0.038 |
| Dietary advice | Unsat | 40 | 1.23 | 0.08 |  |
| Supplement |  | 40 | 1.22 | 0.05 |  |
| Placebo |  | 36 | 1.20 | 0.06 | 0.054 |
| Dietary advice | Glc | 40 | 4.28 | 0.64 |  |
| Supplement |  | 40 | 5.01 | 1.72 |  |
| Placebo |  | 36 | 4.78 | 1.62 | 0.063 |
| Dietary advice | Pyr | 40 | 0.10 | 0.03 |  |
| Supplement |  | 40 | 0.12 | 0.04 |  |
| Placebo |  | 36 | 0.12 | 0.05 | 0.106 |
| Dietary advice | Crea | 40 | 0.06 | 0.01 |  |
| Supplement |  | 40 | 0.06 | 0.01 |  |
| Placebo |  | 36 | 0.07 | 0.01 | 0.120 |
| Dietary advice | Leu | 40 | 0.08 | 0.02 |  |
| Supplement |  | 40 | 0.09 | 0.02 |  |
| Placebo |  | 36 | 0.09 | 0.02 | 0.124 |
| Dietary advice | L_LDL_FC | 40 | 0.22 | 0.05 |  |
| Supplement |  | 40 | 0.19 | 0.07 |  |
| Placebo |  | 36 | 0.19 | 0.06 | 0.125 |
| Dietary advice | L_LDL_PL | 40 | 0.29 | 0.06 |  |
| Supplement |  | 40 | 0.26 | 0.08 |  |
| Placebo |  | 36 | 0.27 | 0.07 | 0.128 |
| Dietary advice | XL_VLDL_FC | 40 | 0.00 | 0.00 |  |
| Supplement |  | 40 | 0.00 | 0.00 |  |
| Placebo |  | 36 | 0.01 | 0.00 | 0.132 |
| Dietary advice | XXL_VLDL_FC | 40 | 0.00 | 0.00 |  |
| Supplement |  | 40 | 0.00 | 0.00 |  |
| Placebo |  | 36 | 0.00 | 0.00 | 0.135 |
| Dietary advice | FAw3_FA | 40 | 4.15 | 1.42 |  |
| Supplement |  | 40 | 4.20 | 0.99 |  |
| Placebo |  | 36 | 3.72 | 0.88 | 0.135 |
| Dietary advice | L_VLDL_FC | 40 | 0.02 | 0.02 |  |
| Supplement |  | 40 | 0.02 | 0.01 |  |
| Placebo |  | 36 | 0.03 | 0.02 | 0.137 |
| Dietary advice | IDL_FC | 40 | 0.17 | 0.04 |  |
| Supplement |  | 40 | 0.15 | 0.06 |  |
| Placebo |  | 36 | 0.16 | 0.05 | 0.140 |
| Dietary advice | IDL_FXL_VLDL_PL | 40 | 0.01 | 0.01 |  |
| Supplement |  | 40 | 0.01 | 0.01 |  |
| Placebo |  | 36 | 0.01 | 0.01 | 0.140 |
| Dietary advice | M_LDL_PL | 40 | 0.18 | 0.03 |  |
| Supplement |  | 40 | 0.16 | 0.04 |  |
| Placebo |  | 36 | 0.17 | 0.04 | 0.140 |
| Dietary advice | S_LDL_CE | 40 | 0.20 | 0.06 |  |
| Supplement |  | 40 | 0.17 | 0.09 |  |
| Placebo |  | 36 | 0.17 | 0.07 | 0.141 |
| Dietary advice | L_LDL_C | 40 | 0.76 | 0.20 |  |
| Supplement |  | 40 | 0.66 | 0.28 |  |
| Placebo |  | 36 | 0.67 | 0.23 | 0.141 |
| Dietary advice | IDL_PL | 40 | 0.27 | 0.05 |  |
| Supplement |  | 40 | 0.24 | 0.08 |  |
| Placebo |  | 36 | 0.24 | 0.07 | 0.144 |
| Dietary advice | XL_VLDL_C | 40 | 0.01 | 0.01 |  |
| Supplement |  | 40 | 0.01 | 0.01 |  |
| Placebo |  | 36 | 0.01 | 0.01 | 0.144 |
| Dietary advice | LDL_C | 40 | 1.48 | 0.41 |  |
| Supplement |  | 40 | 1.28 | 0.55 |  |
| Placebo |  | 36 | 1.30 | 0.46 | 0.146 |
| Dietary advice | XL_VLDL_L | 40 | 0.05 | 0.04 |  |
| Supplement |  | 40 | 0.05 | 0.03 |  |
| Placebo |  | 36 | 0.07 | 0.04 | 0.146 |
| Dietary advice | M_LDL_CE | 40 | 0.32 | 0.10 |  |
| Supplement |  | 40 | 0.27 | 0.14 |  |
| Placebo |  | 36 | 0.27 | 0.12 | 0.146 |
| Dietary advice | L_LDL_CE | 40 | 0.54 | 0.15 |  |
| Supplement |  | 40 | 0.47 | 0.21 |  |
| Placebo |  | 36 | 0.48 | 0.18 | 0.147 |
| Dietary advice | L_LDL_L | 40 | 1.14 | 0.26 |  |
| Supplement |  | 40 | 1.01 | 0.37 |  |
| Placebo |  | 36 | 1.03 | 0.31 | 0.147 |
| Dietary advice | XL_VLDL_P | 40 | 0.00 | 0.00 |  |
| Supplement |  | 40 | 0.00 | 0.00 |  |
| Placebo |  | 36 | 0.00 | 0.00 | 0.147 |
| Dietary advice | M_LDL_C | 40 | 0.45 | 0.13 |  |
| Supplement |  | 40 | 0.39 | 0.17 |  |
| Placebo |  | 36 | 0.39 | 0.14 | 0.151 |
| Dietary advice | XL_VLDL_TG | 40 | 0.03 | 0.02 |  |
| Supplement |  | 40 | 0.03 | 0.02 |  |
| Placebo |  | 36 | 0.04 | 0.03 | 0.152 |
| Dietary advice | L_LDL_P | 40 | 0.00 | 0.00 |  |
| Supplement |  | 40 | 0.00 | 0.00 |  |
| Placebo |  | 36 | 0.00 | 0.00 | 0.152 |
| Dietary advice | S_LDL_C | 40 | 0.27 | 0.08 |  |
| Supplement |  | 40 | 0.24 | 0.11 |  |
| Placebo |  | 36 | 0.24 | 0.09 | 0.153 |
| Dietary advice | M_LDL_L | 40 | 0.67 | 0.16 |  |
| Supplement |  | 40 | 0.59 | 0.22 |  |
| Placebo |  | 36 | 0.61 | 0.19 | 0.155 |
| Dietary advice | XL_VLDL_CE0 | 40 | 0.01 | 0.00 |  |
| Supplement |  | 40 | 0.01 | 0.00 |  |
| Placebo |  | 36 | 0.01 | 0.01 | 0.155 |
| Dietary advice | IDL_C0 | 40 | 0.59 | 0.15 |  |
| Supplement |  | 40 | 0.52 | 0.21 |  |
| Placebo |  | 36 | 0.53 | 0.18 | 0.156 |
| Dietary advice | S_HDL_TG0 | 40 | 0.05 | 0.01 |  |
| Supplement |  | 40 | 0.05 | 0.01 |  |
| Placebo |  | 36 | 0.05 | 0.01 | 0.156 |
| Dietary advice | M_LDL_P0 | 40 | 0.00 | 0.00 |  |
| Supplement |  | 40 | 0.00 | 0.00 |  |
| Placebo |  | 36 | 0.00 | 0.00 | 0.157 |
| Dietary advice | FALen0 | 40 | 17.57 | 0.31 |  |
| Supplement |  | 40 | 17.63 | 0.36 |  |
| Placebo |  | 36 | 17.73 | 0.40 | 0.159 |
| Dietary advice | L_VLDL_C | 40 | 0.05 | 0.03 |  |
| Supplement |  | 40 | 0.05 | 0.03 |  |
| Placebo |  | 36 | 0.06 | 0.04 | 0.160 |
| Dietary advice | L_VLDL_L0 | 40 | 0.23 | 0.13 |  |
| Supplement |  | 40 | 0.21 | 0.11 |  |
| Placebo |  | 36 | 0.27 | 0.15 | 0.160 |
| Dietary advice | L_VLDL_TG0 | 40 | 0.14 | 0.07 |  |
| Supplement |  | 40 | 0.12 | 0.06 |  |
| Placebo |  | 36 | 0.16 | 0.08 | 0.161 |
| Dietary advice | L_VLDL_P0 | 40 | 0.00 | 0.00 |  |
| Supplement |  | 40 | 0.00 | 0.00 |  |
| Placebo |  | 36 | 0.00 | 0.00 | 0.162 |
| Dietary advice | IDL_CE0 | 40 | 0.42 | 0.11 |  |
| Supplement |  | 40 | 0.37 | 0.15 |  |
| Placebo |  | 36 | 0.38 | 0.13 | 0.164 |
| Dietary advice | XIDL_FXL_VLDL_PL | 40 | 0.00 | 0.00 |  |
| Supplement |  | 40 | 0.00 | 0.00 |  |
| Placebo |  | 36 | 0.00 | 0.00 | 0.164 |
| Dietary advice | L_VLDL_PL0 | 40 | 0.04 | 0.02 |  |
| Supplement |  | 40 | 0.04 | 0.02 |  |
| Placebo |  | 36 | 0.05 | 0.03 | 0.164 |
| Dietary advice | IDL_L0 | 40 | 0.96 | 0.21 |  |
| Supplement |  | 40 | 0.86 | 0.30 |  |
| Placebo |  | 36 | 0.88 | 0.26 | 0.166 |
| Dietary advice | VLDL_TG0 | 40 | 0.76 | 0.28 |  |
| Supplement |  | 40 | 0.72 | 0.24 |  |
| Placebo |  | 36 | 0.84 | 0.32 | 0.167 |
| Dietary advice | XS_VLDL_PL0 | 40 | 0.14 | 0.03 |  |
| Supplement |  | 40 | 0.12 | 0.04 |  |
| Placebo |  | 36 | 0.13 | 0.04 | 0.168 |
| Dietary advice | M_VLDL_TG0 | 40 | 0.26 | 0.10 |  |
| Supplement |  | 40 | 0.25 | 0.09 |  |
| Placebo |  | 36 | 0.29 | 0.11 | 0.168 |
| Dietary advice | XXL_VLDL_C | 40 | 0.00 | 0.00 |  |
| Supplement |  | 40 | 0.00 | 0.00 |  |
| Placebo |  | 36 | 0.01 | 0.00 | 0.168 |
| Dietary advice | S_LDL_L0 | 40 | 0.44 | 0.10 |  |
| Supplement |  | 40 | 0.39 | 0.14 |  |
| Placebo |  | 36 | 0.40 | 0.12 | 0.169 |
| Dietary advice | M_VLDL_FC0 | 40 | 0.06 | 0.02 |  |
| Supplement |  | 40 | 0.05 | 0.02 |  |
| Placebo |  | 36 | 0.06 | 0.03 | 0.170 |
| Dietary advice | S_LDL_P0 | 40 | 0.00 | 0.00 |  |
| Supplement |  | 40 | 0.00 | 0.00 |  |
| Placebo |  | 36 | 0.00 | 0.00 | 0.171 |
| Dietary advice | M_LDL_FC0 | 40 | 0.13 | 0.02 |  |
| Supplement |  | 40 | 0.12 | 0.03 |  |
| Placebo |  | 36 | 0.12 | 0.03 | 0.173 |
| Dietary advice | IDL_P0 | 40 | 0.00 | 0.00 |  |
| Supplement |  | 40 | 0.00 | 0.00 |  |
| Placebo |  | 36 | 0.00 | 0.00 | 0.174 |
| Dietary advice | Serum_TG0 | 40 | 1.16 | 0.34 |  |
| Supplement |  | 40 | 1.10 | 0.29 |  |
| Placebo |  | 36 | 1.25 | 0.40 | 0.175 |
| Dietary advice | M_VLDL_P0 | 40 | 0.00 | 0.00 |  |
| Supplement |  | 40 | 0.00 | 0.00 |  |
| Placebo |  | 36 | 0.00 | 0.00 | 0.178 |
| Dietary advice | S_HDL_CE0 | 40 | 0.35 | 0.05 |  |
| Supplement |  | 40 | 0.33 | 0.05 |  |
| Placebo |  | 36 | 0.33 | 0.05 | 0.178 |
| Dietary advice | M_VLDL_L0 | 40 | 0.51 | 0.19 |  |
| Supplement |  | 40 | 0.47 | 0.16 |  |
| Placebo |  | 36 | 0.55 | 0.22 | 0.179 |
| Dietary advice | S_LDL_PL0 | 40 | 0.13 | 0.02 |  |
| Supplement |  | 40 | 0.12 | 0.03 |  |
| Placebo |  | 36 | 0.13 | 0.03 | 0.180 |
| Dietary advice | Serum_C0 | 40 | 4.05 | 0.74 |  |
| Supplement |  | 40 | 3.70 | 1.03 |  |
| Placebo |  | 36 | 3.76 | 0.89 | 0.181 |
| Dietary advice | S_VLDL_TG0 | 40 | 0.21 | 0.06 |  |
| Supplement |  | 40 | 0.20 | 0.05 |  |
| Placebo |  | 36 | 0.22 | 0.07 | 0.182 |
| Dietary advice | M_VLDL_PL0 | 40 | 0.10 | 0.04 |  |
| Supplement |  | 40 | 0.10 | 0.03 |  |
| Placebo |  | 36 | 0.11 | 0.04 | 0.185 |
| Dietary advice | EstC0 | 40 | 2.91 | 0.53 |  |
| Supplement |  | 40 | 2.66 | 0.73 |  |
| Placebo |  | 36 | 2.71 | 0.65 | 0.185 |
| Dietary advice | L_VLDL_CE0 | 40 | 0.03 | 0.02 |  |
| Supplement |  | 40 | 0.03 | 0.01 |  |
| Placebo |  | 36 | 0.04 | 0.02 | 0.185 |
| Dietary advice | FreeC0 | 40 | 1.14 | 0.21 |  |
| Supplement |  | 40 | 1.04 | 0.31 |  |
| Placebo |  | 36 | 1.05 | 0.26 | 0.190 |
| Dietary advice | S_VLDL_PL0 | 40 | 0.13 | 0.02 |  |
| Supplement |  | 40 | 0.12 | 0.02 |  |
| Placebo |  | 36 | 0.13 | 0.03 | 0.191 |
| Dietary advice | XXL_VLDL_L | 40 | 0.02 | 0.02 |  |
| Supplement |  | 40 | 0.02 | 0.01 |  |
| Placebo |  | 36 | 0.03 | 0.02 | 0.191 |
| Dietary advice | Ile0 | 40 | 0.06 | 0.01 |  |
| Supplement |  | 40 | 0.06 | 0.02 |  |
| Placebo |  | 36 | 0.06 | 0.01 | 0.195 |
| Dietary advice | XXL_VLDL_P | 40 | 0.00 | 0.00 |  |
| Supplement |  | 40 | 0.00 | 0.00 |  |
| Placebo |  | 36 | 0.00 | 0.00 | 0.195 |
| Dietary advice | XXL_VLDL_CE | 40 | 0.00 | 0.00 |  |
| Supplement |  | 40 | 0.00 | 0.00 |  |
| Placebo |  | 36 | 0.00 | 0.00 | 0.197 |
| Dietary advice | M_HDL_TG | 40 | 0.05 | 0.01 |  |
| Supplement |  | 40 | 0.04 | 0.01 |  |
| Placebo |  | 36 | 0.05 | 0.01 | 0.197 |
| Dietary advice | S_VLDL_P | 40 | 0.00 | 0.00 |  |
| Supplement |  | 40 | 0.00 | 0.00 |  |
| Placebo |  | 36 | 0.00 | 0.00 | 0.200 |
| Dietary advice | S_VLDL_L | 40 | 0.53 | 0.12 |  |
| Supplement |  | 40 | 0.50 | 0.12 |  |
| Placebo |  | 36 | 0.55 | 0.15 | 0.205 |
| Dietary advice | M_VLDL_C | 40 | 0.14 | 0.05 |  |
| Supplement |  | 40 | 0.13 | 0.05 |  |
| Placebo |  | 36 | 0.15 | 0.06 | 0.205 |
| Dietary advice | XXL_VLDL_TG | 40 | 0.02 | 0.01 |  |
| Supplement |  | 40 | 0.02 | 0.01 |  |
| Placebo |  | 36 | 0.02 | 0.01 | 0.206 |
| Dietary advice | mufa | 40 | 2.46 | 0.62 |  |
| Supplement |  | 40 | 2.46 | 0.55 |  |
| Placebo |  | 36 | 2.68 | 0.66 | 0.211 |
| Dietary advice | S_VLDL_FC | 40 | 0.07 | 0.02 |  |
| Supplement |  | 40 | 0.07 | 0.02 |  |
| Placebo |  | 36 | 0.08 | 0.02 | 0.216 |
| Dietary advice | XS_VLDL_CE | 40 | 0.13 | 0.03 |  |
| Supplement |  | 40 | 0.11 | 0.04 |  |
| Placebo |  | 36 | 0.12 | 0.04 | 0.219 |
| Dietary advice | S_LDL_FC | 40 | 0.08 | 0.02 |  |
| Supplement |  | 40 | 0.07 | 0.02 |  |
| Placebo |  | 36 | 0.07 | 0.02 | 0.221 |
| Dietary advice | XS_VLDL_TG | 40 | 0.10 | 0.02 |  |
| Supplement |  | 40 | 0.10 | 0.02 |  |
| Placebo |  | 36 | 0.11 | 0.03 | 0.221 |
| Dietary advice | XS_VLDL_C | 40 | 0.20 | 0.05 |  |
| Supplement |  | 40 | 0.18 | 0.06 |  |
| Placebo |  | 36 | 0.19 | 0.06 | 0.227 |
| Dietary advice | ApoB0 | 40 | 0.80 | 0.15 |  |
| Supplement |  | 40 | 0.73 | 0.18 |  |
| Placebo |  | 36 | 0.77 | 0.18 | 0.230 |
| Dietary advice | Remnant_C | 40 | 1.20 | 0.30 |  |
| Supplement |  | 40 | 1.06 | 0.37 |  |
| Placebo |  | 36 | 1.15 | 0.37 | 0.230 |
| Dietary advice | XS_VLDL_L | 40 | 0.44 | 0.09 |  |
| Supplement |  | 40 | 0.40 | 0.11 |  |
| Placebo |  | 36 | 0.42 | 0.11 | 0.232 |
| Dietary advice | M_VLDL_CE | 40 | 0.08 | 0.03 |  |
| Supplement |  | 40 | 0.08 | 0.03 |  |
| Placebo |  | 36 | 0.09 | 0.04 | 0.232 |
| Dietary advice | XS_VLDL_P | 40 | 0.00 | 0.00 |  |
| Supplement |  | 40 | 0.00 | 0.00 |  |
| Placebo |  | 36 | 0.00 | 0.00 | 0.236 |
| Dietary advice | HDL_TG | 40 | 0.13 | 0.02 |  |
| Supplement |  | 40 | 0.13 | 0.02 |  |
| Placebo |  | 36 | 0.14 | 0.02 | 0.237 |
| Dietary advice | VLDL_C | 40 | 0.60 | 0.17 |  |
| Supplement |  | 40 | 0.54 | 0.18 |  |
| Placebo |  | 36 | 0.61 | 0.22 | 0.237 |
| Dietary advice | Ace | 40 | 0.04 | 0.01 |  |
| Supplement |  | 40 | 0.04 | 0.01 |  |
| Placebo |  | 36 | 0.04 | 0.01 | 0.238 |
| Dietary advice | S_VLDL_C | 40 | 0.19 | 0.05 |  |
| Supplement |  | 40 | 0.17 | 0.06 |  |
| Placebo |  | 36 | 0.19 | 0.06 | 0.250 |
| Dietary advice | DHA_FA | 40 | 1.31 | 0.55 |  |
| Supplement |  | 40 | 1.34 | 0.35 |  |
| Placebo |  | 36 | 1.18 | 0.37 | 0.253 |
| Dietary advice | S_VLDL_CE | 40 | 0.12 | 0.03 |  |
| Supplement |  | 40 | 0.10 | 0.04 |  |
| Placebo |  | 36 | 0.11 | 0.04 | 0.266 |
| Dietary advice | XS_VLDL_FC | 40 | 0.07 | 0.01 |  |
| Supplement |  | 40 | 0.06 | 0.02 |  |
| Placebo |  | 36 | 0.07 | 0.02 | 0.270 |
| Dietary advice | Lac | 40 | 1.84 | 0.48 |  |
| Supplement |  | 40 | 2.03 | 0.48 |  |
| Placebo |  | 36 | 1.91 | 0.63 | 0.277 |
| Dietary advice | VLDL_D | 40 | 36.82 | 0.83 |  |
| Supplement |  | 40 | 36.87 | 0.98 |  |
| Placebo |  | 36 | 37.13 | 0.95 | 0.303 |
| Dietary advice | S_HDL_C | 40 | 0.46 | 0.06 |  |
| Supplement |  | 40 | 0.45 | 0.05 |  |
| Placebo |  | 36 | 0.45 | 0.05 | 0.330 |
| Dietary advice | XL_HDL_PL | 40 | 0.18 | 0.07 |  |
| Supplement |  | 40 | 0.17 | 0.08 |  |
| Placebo |  | 36 | 0.15 | 0.08 | 0.334 |
| Dietary advice | S_LDL_TG | 40 | 0.03 | 0.01 |  |
| Supplement |  | 40 | 0.03 | 0.01 |  |
| Placebo |  | 36 | 0.03 | 0.01 | 0.336 |
| Dietary advice | IDL_TG | 40 | 0.10 | 0.02 |  |
| Supplement |  | 40 | 0.10 | 0.02 |  |
| Placebo |  | 36 | 0.10 | 0.02 | 0.339 |
| Dietary advice | dag | 36 | 0.01 | 0.01 |  |
| Supplement |  | 39 | 0.02 | 0.01 |  |
| Placebo |  | 34 | 0.02 | 0.01 | 0.364 |
| Dietary advice | L_LDL_TG | 40 | 0.09 | 0.01 |  |
| Supplement |  | 40 | 0.09 | 0.02 |  |
| Placebo |  | 36 | 0.09 | 0.02 | 0.374 |
| Dietary advice | LDL_TG | 40 | 0.17 | 0.03 |  |
| Supplement |  | 40 | 0.16 | 0.03 |  |
| Placebo |  | 36 | 0.17 | 0.04 | 0.375 |
| Dietary advice | la | 40 | 2.60 | 0.40 |  |
| Supplement |  | 40 | 2.45 | 0.55 |  |
| Placebo |  | 36 | 2.50 | 0.54 | 0.381 |
| Dietary advice | M_LDL_TG | 40 | 0.05 | 0.01 |  |
| Supplement |  | 40 | 0.04 | 0.01 |  |
| Placebo |  | 36 | 0.05 | 0.01 | 0.403 |
| Dietary advice | HDL_D | 40 | 9.90 | 0.15 |  |
| Supplement |  | 40 | 9.89 | 0.19 |  |
| Placebo |  | 36 | 9.85 | 0.17 | 0.417 |
| Dietary advice | Val | 40 | 0.18 | 0.03 |  |
| Supplement |  | 40 | 0.19 | 0.04 |  |
| Placebo |  | 36 | 0.19 | 0.04 | 0.440 |
| Dietary advice | XL_HDL_P | 40 | 0.00 | 0.00 |  |
| Supplement |  | 40 | 0.00 | 0.00 |  |
| Placebo |  | 36 | 0.00 | 0.00 | 0.442 |
| Dietary advice | XL_HDL_L | 40 | 0.35 | 0.11 |  |
| Supplement |  | 40 | 0.33 | 0.15 |  |
| Placebo |  | 36 | 0.31 | 0.14 | 0.442 |
| Dietary advice | SFA_FA | 40 | 37.37 | 2.00 |  |
| Supplement |  | 40 | 37.83 | 1.70 |  |
| Placebo |  | 36 | 37.90 | 2.35 | 0.463 |
| Dietary advice | L_HDL_FC | 40 | 0.07 | 0.03 |  |
| Supplement |  | 40 | 0.07 | 0.03 |  |
| Placebo |  | 36 | 0.06 | 0.03 | 0.495 |
| Dietary advice | XL_HDL_FC | 40 | 0.04 | 0.01 |  |
| Supplement |  | 40 | 0.04 | 0.02 |  |
| Placebo |  | 36 | 0.03 | 0.02 | 0.501 |
| Dietary advice | L_HDL_C | 40 | 0.32 | 0.11 |  |
| Supplement |  | 40 | 0.32 | 0.12 |  |
| Placebo |  | 36 | 0.30 | 0.12 | 0.520 |
| Dietary advice | L_HDL_PL | 40 | 0.35 | 0.10 |  |
| Supplement |  | 40 | 0.35 | 0.11 |  |
| Placebo |  | 36 | 0.33 | 0.11 | 0.525 |
| Dietary advice | HDL2_C | 40 | 0.90 | 0.22 |  |
| Supplement |  | 40 | 0.88 | 0.24 |  |
| Placebo |  | 36 | 0.84 | 0.23 | 0.527 |
| Dietary advice | L_HDL_CE | 40 | 0.25 | 0.09 |  |
| Supplement |  | 40 | 0.25 | 0.09 |  |
| Placebo |  | 36 | 0.23 | 0.09 | 0.529 |
| Dietary advice | HDL_C | 40 | 1.37 | 0.23 |  |
| Supplement |  | 40 | 1.35 | 0.26 |  |
| Placebo |  | 36 | 1.31 | 0.26 | 0.529 |
| Dietary advice | FAw3 | 40 | 0.41 | 0.18 |  |
| Supplement |  | 40 | 0.41 | 0.12 |  |
| Placebo |  | 36 | 0.37 | 0.13 | 0.542 |
| Dietary advice | L_HDL_L | 40 | 0.69 | 0.22 |  |
| Supplement |  | 40 | 0.70 | 0.23 |  |
| Placebo |  | 36 | 0.64 | 0.23 | 0.544 |
| Dietary advice | S_HDL_FC | 40 | 0.11 | 0.01 |  |
| Supplement |  | 40 | 0.11 | 0.01 |  |
| Placebo |  | 36 | 0.11 | 0.01 | 0.551 |
| Dietary advice | L_HDL_P | 40 | 0.00 | 0.00 |  |
| Supplement |  | 40 | 0.00 | 0.00 |  |
| Placebo |  | 36 | 0.00 | 0.00 | 0.552 |
| Dietary advice | Glol | 40 | 0.05 | 0.02 |  |
| Supplement |  | 40 | 0.05 | 0.02 |  |
| Placebo |  | 36 | 0.05 | 0.01 | 0.559 |
| Dietary advice | sfa | 40 | 3.66 | 0.69 |  |
| Supplement |  | 40 | 3.63 | 0.56 |  |
| Placebo |  | 36 | 3.78 | 0.75 | 0.562 |
| Dietary advice | FAw6 | 40 | 3.26 | 0.50 |  |
| Supplement |  | 40 | 3.13 | 0.60 |  |
| Placebo |  | 36 | 3.16 | 0.60 | 0.562 |
| Dietary advice | XL_HDL_C | 40 | 0.16 | 0.05 |  |
| Supplement |  | 40 | 0.15 | 0.06 |  |
| Placebo |  | 36 | 0.14 | 0.06 | 0.564 |
| Dietary advice | pufa | 40 | 3.67 | 0.59 |  |
| Supplement |  | 40 | 3.53 | 0.67 |  |
| Placebo |  | 36 | 3.54 | 0.68 | 0.567 |
| Dietary advice | S_HDL_PL | 40 | 0.59 | 0.06 |  |
| Supplement |  | 40 | 0.60 | 0.07 |  |
| Placebo |  | 36 | 0.61 | 0.07 | 0.568 |
| Dietary advice | ApoA1 | 40 | 1.48 | 0.15 |  |
| Supplement |  | 40 | 1.46 | 0.17 |  |
| Placebo |  | 36 | 1.45 | 0.16 | 0.580 |
| Dietary advice | XL_HDL_CE | 40 | 0.12 | 0.03 |  |
| Supplement |  | 40 | 0.12 | 0.05 |  |
| Placebo |  | 36 | 0.11 | 0.04 | 0.595 |
| Dietary advice | dha | 40 | 0.13 | 0.07 |  |
| Supplement |  | 40 | 0.13 | 0.04 |  |
| Placebo |  | 36 | 0.12 | 0.05 | 0.614 |
| Dietary advice | HDL3_C | 40 | 0.47 | 0.02 |  |
| Supplement |  | 40 | 0.47 | 0.03 |  |
| Placebo |  | 36 | 0.46 | 0.03 | 0.619 |
| Dietary advice | His | 40 | 0.07 | 0.01 |  |
| Supplement |  | 40 | 0.07 | 0.01 |  |
| Placebo |  | 36 | 0.07 | 0.01 | 0.645 |
| Dietary advice | TotFA | 40 | 9.79 | 1.76 |  |
| Supplement |  | 40 | 9.62 | 1.60 |  |
| Placebo |  | 36 | 10.00 | 1.93 | 0.648 |
| Dietary advice | Cit | 39 | 0.08 | 0.02 |  |
| Supplement |  | 37 | 0.09 | 0.01 |  |
| Placebo |  | 34 | 0.08 | 0.01 | 0.677 |
| Dietary advice | XL_HDL_TG | 40 | 0.01 | 0.00 |  |
| Supplement |  | 40 | 0.01 | 0.01 |  |
| Placebo |  | 36 | 0.01 | 0.01 | 0.721 |
| Dietary advice | Tyr | 40 | 0.06 | 0.01 |  |
| Supplement |  | 40 | 0.06 | 0.01 |  |
| Placebo |  | 36 | 0.06 | 0.01 | 0.763 |
| Dietary advice | bOHBut | 40 | 0.11 | 0.04 |  |
| Supplement |  | 40 | 0.11 | 0.04 |  |
| Placebo |  | 36 | 0.12 | 0.07 | 0.772 |
| Dietary advice | S_HDL_P | 40 | 0.00 | 0.00 |  |
| Supplement |  | 40 | 0.00 | 0.00 |  |
| Placebo |  | 36 | 0.00 | 0.00 | 0.774 |
| Dietary advice | cla | 39 | 0.02 | 0.01 |  |
| Supplement |  | 40 | 0.02 | 0.01 |  |
| Placebo |  | 36 | 0.03 | 0.02 | 0.783 |
| Dietary advice | M_HDL_FC | 40 | 0.08 | 0.02 |  |
| Supplement |  | 40 | 0.08 | 0.02 |  |
| Placebo |  | 36 | 0.08 | 0.02 | 0.783 |
| Dietary advice | S_HDL_L | 40 | 1.11 | 0.10 |  |
| Supplement |  | 40 | 1.10 | 0.09 |  |
| Placebo |  | 36 | 1.11 | 0.11 | 0.806 |
| Dietary advice | Gln | 40 | 0.54 | 0.08 |  |
| Supplement |  | 40 | 0.55 | 0.08 |  |
| Placebo |  | 36 | 0.55 | 0.07 | 0.817 |
| Dietary advice | M_HDL_C | 40 | 0.43 | 0.08 |  |
| Supplement |  | 40 | 0.43 | 0.07 |  |
| Placebo |  | 36 | 0.42 | 0.08 | 0.818 |
| Dietary advice | M_HDL_CE | 40 | 0.35 | 0.06 |  |
| Supplement |  | 40 | 0.35 | 0.06 |  |
| Placebo |  | 36 | 0.34 | 0.07 | 0.827 |
| Dietary advice | CLA_FA | 39 | 0.23 | 0.13 |  |
| Supplement |  | 40 | 0.22 | 0.12 |  |
| Placebo |  | 36 | 0.24 | 0.13 | 0.834 |
| Dietary advice | M_HDL_L | 40 | 0.89 | 0.15 |  |
| Supplement |  | 40 | 0.87 | 0.13 |  |
| Placebo |  | 36 | 0.87 | 0.15 | 0.847 |
| Dietary advice | M_HDL_PL | 40 | 0.41 | 0.07 |  |
| Supplement |  | 40 | 0.40 | 0.06 |  |
| Placebo |  | 36 | 0.40 | 0.06 | 0.850 |
| Dietary advice | Alb | 40 | 0.09 | 0.01 |  |
| Supplement |  | 40 | 0.09 | 0.01 |  |
| Placebo |  | 36 | 0.09 | 0.01 | 0.850 |
| Dietary advice | M_HDL_P | 40 | 0.00 | 0.00 |  |
| Supplement |  | 40 | 0.00 | 0.00 |  |
| Placebo |  | 36 | 0.00 | 0.00 | 0.850 |
| Dietary advice | TotPG | 40 | 1.59 | 0.30 |  |
| Supplement |  | 40 | 1.58 | 0.27 |  |
| Placebo |  | 36 | 1.61 | 0.28 | 0.924 |
| Dietary advice | pc | 40 | 1.73 | 0.31 |  |
| Supplement |  | 40 | 1.71 | 0.28 |  |
| Placebo |  | 36 | 1.73 | 0.29 | 0.926 |
| Dietary advice | sm | 40 | 0.41 | 0.07 |  |
| Supplement |  | 40 | 0.41 | 0.09 |  |
| Placebo |  | 36 | 0.41 | 0.08 | 0.945 |
| Dietary advice | LDL_D | 40 | 23.49 | 0.09 |  |
| Supplement |  | 40 | 23.49 | 0.11 |  |
| Placebo |  | 36 | 23.50 | 0.10 | 0.949 |
| Dietary advice | TotCho | 40 | 2.01 | 0.35 |  |
| Supplement |  | 40 | 2.00 | 0.32 |  |
| Placebo |  | 36 | 2.02 | 0.32 | 0.962 |
| Dietary advice | L_HDL_TG | 40 | 0.02 | 0.01 |  |
| Supplement |  | 40 | 0.02 | 0.01 |  |
| Placebo |  | 36 | 0.02 | 0.01 | 0.989 |

SD = standard deviation.

p-value estimated using ANOVA.

*Means are expressed in absolute concentration units (e.g. mmol/l).

For abbreviations of metabolic trait names, see Supplementary table 8

**Supplementary Table 4**: lycopene unadjusted regression results

| **Metabolite** | **Intervention arm** | **N** | **Mean difference†**  **(concentration)*** | | **Lower CI**  **(concentration)*** | | **Upper CI**  **(concentration)*** | | **p-value** |
| --- | --- | --- | --- | --- | --- | --- | --- | --- | --- |
| Val | Dietary advice | 128 | -0.65 | (-0.02) | -1.04 | (-0.04) | -0.26 | (-0.01) | 0.001 |
| Ace | Supplement | 128 | 0.69 | (0.01) | 0.24 | (0.00) | 1.15 | (0.01) | 0.003 |
| Val | Supplement | 128 | -0.62 | (-0.02) | -1.03 | (-0.04) | -0.20 | (-0.01) | 0.004 |
| dag | Dietary advice | 127 | -0.59 | (-0.01) | -1.01 | (-0.01) | -0.18 | (-0.00) | 0.006 |
| Pyr | Supplement | 128 | -0.56 | (-0.02) | -0.95 | (-0.04) | -0.16 | (-0.01) | 0.006 |
| dha | Supplement | 128 | -0.50 | (-0.03) | -0.85 | (-0.05) | -0.14 | (-0.01) | 0.006 |
| sfa | Dietary advice | 128 | -0.57 | (-0.49) | -0.97 | (-0.84) | -0.16 | (-0.14) | 0.007 |
| cla | Supplement | 127 | -0.61 | (-0.01) | -1.06 | (-0.01) | -0.16 | (-0.00) | 0.009 |
| TotFA | Dietary advice | 128 | -0.53 | (-1.18) | -0.94 | (-2.09) | -0.12 | (-0.27) | 0.012 |
| Leu | Dietary advice | 128 | -0.50 | (-0.01) | -0.90 | (-0.02) | -0.11 | (-0.00) | 0.013 |
| Ile | Dietary advice | 128 | -0.51 | (-0.01) | -0.91 | (-0.02) | -0.11 | (-0.00) | 0.013 |
| XL_HDL_TG | Dietary advice | 128 | -0.53 | (-0.00) | -0.95 | (-0.01) | -0.11 | (-0.00) | 0.013 |
| Tyr | Supplement | 128 | -0.56 | (-0.01) | -1.00 | (-0.01) | -0.12 | (-0.00) | 0.014 |
| HDL_TG | Dietary advice | 128 | -0.54 | (-0.02) | -0.96 | (-0.03) | -0.11 | (-0.00) | 0.014 |
| mufa | Dietary advice | 128 | -0.52 | (-0.39) | -0.95 | (-0.70) | -0.10 | (-0.08) | 0.015 |
| XS_VLDL_TG | Supplement | 128 | -0.54 | (-0.01) | -0.98 | (-0.03) | -0.10 | (-0.00) | 0.016 |
| M_VLDL_C | Dietary advice | 128 | -0.51 | (-0.04) | -0.93 | (-0.06) | -0.09 | (-0.01) | 0.017 |
| S_VLDL_P | Supplement | 128 | -0.53 | (-0.00) | -0.97 | (-0.00) | -0.10 | (-0.00) | 0.017 |
| S_VLDL_L | Supplement | 128 | -0.53 | (-0.08) | -0.97 | (-0.15) | -0.10 | (-0.02) | 0.017 |
| M_VLDL_CE | Dietary advice | 128 | -0.51 | (-0.02) | -0.92 | (-0.03) | -0.09 | (-0.00) | 0.017 |
| VLDL_C | Dietary advice | 128 | -0.50 | (-0.11) | -0.92 | (-0.19) | -0.09 | (-0.02) | 0.018 |
| S_VLDL_FC | Supplement | 128 | -0.52 | (-0.01) | -0.96 | (-0.02) | -0.09 | (-0.00) | 0.019 |
| M_VLDL_PL | Supplement | 128 | -0.51 | (-0.03) | -0.95 | (-0.05) | -0.08 | (-0.00) | 0.020 |
| XXL_VLDL_C | Dietary advice | 128 | -0.50 | (-0.00) | -0.93 | (-0.00) | -0.08 | (-0.00) | 0.021 |
| M_VLDL_L | Supplement | 128 | -0.51 | (-0.13) | -0.95 | (-0.24) | -0.08 | (-0.02) | 0.021 |
| M_VLDL_C | Supplement | 128 | -0.51 | (-0.03) | -0.94 | (-0.06) | -0.08 | (-0.01) | 0.021 |
| DHA_FA | Supplement | 128 | -0.39 | (-0.18) | -0.72 | (-0.33) | -0.06 | (-0.03) | 0.021 |
| M_VLDL_P | Supplement | 128 | -0.51 | (-0.00) | -0.95 | (-0.00) | -0.08 | (-0.00) | 0.021 |
| S_VLDL_TG | Supplement | 128 | -0.52 | (-0.04) | -0.96 | (-0.07) | -0.08 | (-0.01) | 0.021 |
| M_VLDL_FC | Dietary advice | 128 | -0.49 | (-0.02) | -0.91 | (-0.03) | -0.07 | (-0.00) | 0.022 |
| XXL_VLDL_PL | Dietary advice | 128 | -0.50 | (-0.00) | -0.93 | (-0.00) | -0.07 | (-0.00) | 0.022 |
| XXL_VLDL_CE | Dietary advice | 128 | -0.49 | (-0.00) | -0.92 | (-0.00) | -0.07 | (-0.00) | 0.022 |
| XXL_VLDL_FC | Dietary advice | 128 | -0.50 | (-0.00) | -0.93 | (-0.00) | -0.07 | (-0.00) | 0.022 |
| VLDL_C | Supplement | 128 | -0.50 | (-0.10) | -0.93 | (-0.19) | -0.07 | (-0.01) | 0.023 |
| XL_VLDL_FC | Dietary advice | 128 | -0.50 | (-0.00) | -0.93 | (-0.00) | -0.07 | (-0.00) | 0.023 |
| XXL_VLDL_L | Dietary advice | 128 | -0.49 | (-0.01) | -0.92 | (-0.02) | -0.07 | (-0.00) | 0.023 |
| M_VLDL_TG | Supplement | 128 | -0.51 | (-0.07) | -0.94 | (-0.13) | -0.07 | (-0.01) | 0.023 |
| XXL_VLDL_P | Dietary advice | 128 | -0.49 | (-0.00) | -0.92 | (-0.00) | -0.07 | (-0.00) | 0.023 |
| M_VLDL_FC | Supplement | 128 | -0.50 | (-0.02) | -0.93 | (-0.03) | -0.07 | (-0.00) | 0.023 |
| Ala | Supplement | 128 | -0.48 | (-0.03) | -0.89 | (-0.06) | -0.06 | (-0.00) | 0.024 |
| XXL_VLDL_TG | Dietary advice | 128 | -0.49 | (-0.01) | -0.91 | (-0.01) | -0.07 | (-0.00) | 0.024 |
| XL_VLDL_C | Dietary advice | 128 | -0.49 | (-0.01) | -0.92 | (-0.01) | -0.07 | (-0.00) | 0.024 |
| cla | Dietary advice | 127 | -0.48 | (-0.01) | -0.91 | (-0.01) | -0.06 | (-0.00) | 0.024 |
| Serum_TG | Dietary advice | 128 | -0.49 | (-0.22) | -0.92 | (-0.41) | -0.06 | (-0.03) | 0.024 |
| M_VLDL_CE | Supplement | 128 | -0.49 | (-0.02) | -0.92 | (-0.03) | -0.07 | (-0.00) | 0.024 |
| XL_VLDL_PL | Dietary advice | 128 | -0.49 | (-0.00) | -0.93 | (-0.01) | -0.06 | (-0.00) | 0.025 |
| L_VLDL_C | Dietary advice | 128 | -0.49 | (-0.02) | -0.91 | (-0.04) | -0.06 | (-0.00) | 0.025 |
| M_VLDL_PL | Dietary advice | 128 | -0.48 | (-0.02) | -0.89 | (-0.04) | -0.06 | (-0.00) | 0.025 |
| M_VLDL_L | Dietary advice | 128 | -0.48 | (-0.12) | -0.89 | (-0.23) | -0.06 | (-0.02) | 0.025 |
| L_VLDL_FC | Dietary advice | 128 | -0.49 | (-0.01) | -0.92 | (-0.02) | -0.06 | (-0.00) | 0.025 |
| L_VLDL_CE | Dietary advice | 128 | -0.48 | (-0.01) | -0.91 | (-0.02) | -0.06 | (-0.00) | 0.026 |
| XL_VLDL_CE | Dietary advice | 128 | -0.49 | (-0.00) | -0.91 | (-0.01) | -0.06 | (-0.00) | 0.026 |
| mufa | Supplement | 128 | -0.47 | (-0.35) | -0.89 | (-0.66) | -0.06 | (-0.04) | 0.026 |
| S_VLDL_FC | Dietary advice | 128 | -0.46 | (-0.01) | -0.86 | (-0.02) | -0.06 | (-0.00) | 0.026 |
| S_VLDL_PL | Supplement | 128 | -0.50 | (-0.02) | -0.95 | (-0.03) | -0.06 | (-0.00) | 0.026 |
| M_VLDL_P | Dietary advice | 128 | -0.47 | (-0.00) | -0.89 | (-0.00) | -0.06 | (-0.00) | 0.027 |
| L_VLDL_PL | Dietary advice | 128 | -0.48 | (-0.02) | -0.91 | (-0.03) | -0.05 | (-0.00) | 0.027 |
| IDL_TG | Dietary advice | 128 | -0.44 | (-0.01) | -0.83 | (-0.02) | -0.05 | (-0.00) | 0.027 |
| XL_VLDL_L | Dietary advice | 128 | -0.48 | (-0.03) | -0.91 | (-0.05) | -0.05 | (-0.00) | 0.027 |
| XL_VLDL_P | Dietary advice | 128 | -0.48 | (-0.00) | -0.91 | (-0.00) | -0.05 | (-0.00) | 0.028 |
| S_LDL_TG | Dietary advice | 128 | -0.45 | (-0.00) | -0.85 | (-0.01) | -0.05 | (-0.00) | 0.028 |
| bOHBut | Supplement | 128 | 0.52 | (0.02) | 0.06 | (0.00) | 0.98 | (0.04) | 0.029 |
| L_VLDL_L | Dietary advice | 128 | -0.48 | (-0.08) | -0.90 | (-0.16) | -0.05 | (-0.01) | 0.029 |
| L_VLDL_P | Dietary advice | 128 | -0.47 | (-0.00) | -0.90 | (-0.00) | -0.05 | (-0.00) | 0.029 |
| XL_VLDL_TG | Dietary advice | 128 | -0.47 | (-0.02) | -0.90 | (-0.03) | -0.05 | (-0.00) | 0.030 |
| L_VLDL_CE | Supplement | 128 | -0.48 | (-0.01) | -0.91 | (-0.02) | -0.05 | (-0.00) | 0.031 |
| pc | Dietary advice | 127 | -0.43 | (-0.16) | -0.82 | (-0.30) | -0.04 | (-0.02) | 0.031 |
| S_VLDL_C | Supplement | 128 | -0.48 | (-0.03) | -0.91 | (-0.05) | -0.04 | (-0.00) | 0.031 |
| L_VLDL_TG | Dietary advice | 128 | -0.47 | (-0.05) | -0.89 | (-0.09) | -0.04 | (-0.00) | 0.032 |
| Leu | Supplement | 128 | -0.46 | (-0.01) | -0.89 | (-0.02) | -0.04 | (-0.00) | 0.032 |
| S_VLDL_L | Dietary advice | 128 | -0.44 | (-0.07) | -0.84 | (-0.13) | -0.04 | (-0.01) | 0.032 |
| VLDL_TG | Dietary advice | 128 | -0.46 | (-0.18) | -0.89 | (-0.34) | -0.04 | (-0.01) | 0.033 |
| M_VLDL_TG | Dietary advice | 128 | -0.45 | (-0.06) | -0.87 | (-0.12) | -0.04 | (-0.01) | 0.033 |
| IDL_TG | Supplement | 128 | -0.49 | (-0.01) | -0.94 | (-0.02) | -0.04 | (-0.00) | 0.033 |
| S_VLDL_P | Dietary advice | 128 | -0.44 | (-0.00) | -0.84 | (-0.00) | -0.03 | (-0.00) | 0.034 |
| XL_VLDL_CE | Supplement | 128 | -0.47 | (-0.00) | -0.90 | (-0.01) | -0.04 | (-0.00) | 0.034 |
| Remnant_C | Dietary advice | 128 | -0.44 | (-0.16) | -0.85 | (-0.30) | -0.03 | (-0.01) | 0.034 |
| XS_VLDL_TG | Dietary advice | 128 | -0.43 | (-0.01) | -0.83 | (-0.02) | -0.03 | (-0.00) | 0.034 |
| ApoB | Dietary advice | 128 | -0.43 | (-0.08) | -0.83 | (-0.15) | -0.03 | (-0.01) | 0.035 |
| dag | Supplement | 127 | -0.47 | (-0.01) | -0.90 | (-0.01) | -0.03 | (-0.00) | 0.036 |
| L_LDL_TG | Dietary advice | 128 | -0.42 | (-0.01) | -0.80 | (-0.02) | -0.03 | (-0.00) | 0.036 |
| Crea | Dietary advice | 128 | -0.34 | (-0.00) | -0.65 | (-0.01) | -0.02 | (-0.00) | 0.036 |
| FAw3 | Supplement | 128 | -0.39 | (-0.06) | -0.76 | (-0.12) | -0.03 | (-0.00) | 0.036 |
| XXL_VLDL_L | Supplement | 128 | -0.46 | (-0.01) | -0.89 | (-0.02) | -0.03 | (-0.00) | 0.036 |
| XXL_VLDL_P | Supplement | 128 | -0.46 | (-0.00) | -0.89 | (-0.00) | -0.03 | (-0.00) | 0.037 |
| L_VLDL_C | Supplement | 128 | -0.46 | (-0.02) | -0.89 | (-0.04) | -0.03 | (-0.00) | 0.037 |
| XXL_VLDL_TG | Supplement | 128 | -0.46 | (-0.01) | -0.89 | (-0.01) | -0.03 | (-0.00) | 0.037 |
| XXL_VLDL_C | Supplement | 128 | -0.46 | (-0.00) | -0.90 | (-0.00) | -0.03 | (-0.00) | 0.037 |
| XXL_VLDL_FC | Supplement | 128 | -0.46 | (-0.00) | -0.89 | (-0.00) | -0.03 | (-0.00) | 0.037 |
| XL_VLDL_C | Supplement | 128 | -0.46 | (-0.01) | -0.89 | (-0.01) | -0.03 | (-0.00) | 0.038 |
| XXL_VLDL_PL | Supplement | 128 | -0.45 | (-0.00) | -0.88 | (-0.00) | -0.02 | (-0.00) | 0.038 |
| LDL_TG | Dietary advice | 128 | -0.44 | (-0.02) | -0.85 | (-0.03) | -0.02 | (-0.00) | 0.039 |
| S_VLDL_C | Dietary advice | 128 | -0.43 | (-0.02) | -0.83 | (-0.05) | -0.02 | (-0.00) | 0.039 |
| la | Dietary advice | 128 | -0.43 | (-0.28) | -0.85 | (-0.54) | -0.02 | (-0.01) | 0.040 |
| His | Supplement | 128 | -0.45 | (-0.00) | -0.89 | (-0.01) | -0.02 | (-0.00) | 0.041 |
| Serum_TG | Supplement | 128 | -0.45 | (-0.20) | -0.88 | (-0.39) | -0.02 | (-0.01) | 0.042 |
| XXL_VLDL_CE | Supplement | 128 | -0.45 | (-0.00) | -0.89 | (-0.00) | -0.02 | (-0.00) | 0.042 |
| XL_VLDL_FC | Supplement | 128 | -0.44 | (-0.00) | -0.87 | (-0.00) | -0.01 | (-0.00) | 0.044 |
| TotCho | Dietary advice | 128 | -0.40 | (-0.16) | -0.78 | (-0.31) | -0.01 | (-0.00) | 0.045 |
| TotPG | Dietary advice | 128 | -0.40 | (-0.14) | -0.79 | (-0.27) | -0.01 | (-0.00) | 0.045 |
| S_VLDL_TG | Dietary advice | 128 | -0.42 | (-0.03) | -0.82 | (-0.06) | -0.01 | (-0.00) | 0.045 |
| Lac | Supplement | 128 | -0.41 | (-0.27) | -0.82 | (-0.53) | -0.01 | (-0.01) | 0.045 |
| L_VLDL_FC | Supplement | 128 | -0.44 | (-0.01) | -0.87 | (-0.02) | -0.01 | (-0.00) | 0.046 |
| VLDL_TG | Supplement | 128 | -0.44 | (-0.17) | -0.87 | (-0.33) | -0.01 | (-0.00) | 0.046 |
| L_VLDL_PL | Supplement | 128 | -0.44 | (-0.01) | -0.87 | (-0.03) | 0.00 | (-0.00) | 0.048 |
| L_VLDL_L | Supplement | 128 | -0.43 | (-0.08) | -0.87 | (-0.15) | 0.00 | (-0.00) | 0.049 |
| Ile | Supplement | 128 | -0.43 | (-0.01) | -0.86 | (-0.01) | 0.00 | (-0.00) | 0.049 |
| XL_VLDL_PL | Supplement | 128 | -0.43 | (-0.00) | -0.86 | (-0.01) | 0.00 | (-0.00) | 0.049 |
| L_HDL_TG | Dietary advice | 128 | -0.40 | (-0.00) | -0.80 | (-0.01) | 0.00 | (-0.00) | 0.049 |
| S_VLDL_PL | Dietary advice | 128 | -0.39 | (-0.01) | -0.79 | (-0.03) | 0.00 | (0.00) | 0.051 |
| L_VLDL_P | Supplement | 128 | -0.43 | (-0.00) | -0.87 | (-0.00) | 0.00 | (0.00) | 0.051 |
| FAw6 | Dietary advice | 128 | -0.40 | (-0.29) | -0.81 | (-0.58) | 0.00 | (0.00) | 0.053 |
| XL_VLDL_L | Supplement | 128 | -0.43 | (-0.02) | -0.86 | (-0.05) | 0.01 | (0.00) | 0.053 |
| XL_VLDL_P | Supplement | 128 | -0.42 | (-0.00) | -0.86 | (-0.00) | 0.01 | (0.00) | 0.055 |
| XS_VLDL_P | Dietary advice | 128 | -0.38 | (-0.00) | -0.78 | (-0.00) | 0.01 | (0.00) | 0.056 |
| CLA_FA | Supplement | 127 | -0.43 | (-0.05) | -0.88 | (-0.10) | 0.01 | (0.00) | 0.057 |
| L_VLDL_TG | Supplement | 128 | -0.42 | (-0.04) | -0.86 | (-0.09) | 0.01 | (0.00) | 0.057 |
| VLDL_D | Dietary advice | 128 | -0.37 | (-0.44) | -0.76 | (-0.90) | 0.01 | (0.02) | 0.059 |
| UnSat | Dietary advice | 128 | 0.42 | (0.03) | -0.02 | (-0.00) | 0.86 | (0.06) | 0.060 |
| S_VLDL_CE | Supplement | 128 | -0.42 | (-0.02) | -0.86 | (-0.03) | 0.02 | (0.00) | 0.062 |
| His | Dietary advice | 128 | -0.39 | (-0.00) | -0.81 | (-0.01) | 0.02 | (0.00) | 0.062 |
| XL_VLDL_TG | Supplement | 128 | -0.41 | (-0.01) | -0.85 | (-0.03) | 0.02 | (0.00) | 0.063 |
| XS_VLDL_L | Dietary advice | 128 | -0.37 | (-0.04) | -0.76 | (-0.08) | 0.03 | (0.00) | 0.067 |
| XS_VLDL_P | Supplement | 128 | -0.42 | (-0.00) | -0.87 | (-0.00) | 0.03 | (0.00) | 0.067 |
| pufa | Dietary advice | 128 | -0.38 | (-0.30) | -0.79 | (-0.63) | 0.03 | (0.02) | 0.068 |
| Phe | Supplement | 128 | -0.42 | (-0.01) | -0.86 | (-0.01) | 0.03 | (0.00) | 0.068 |
| M_LDL_TG | Dietary advice | 128 | -0.36 | (-0.00) | -0.74 | (-0.01) | 0.03 | (0.00) | 0.070 |
| S_VLDL_CE | Dietary advice | 128 | -0.37 | (-0.01) | -0.78 | (-0.03) | 0.03 | (0.00) | 0.072 |
| M_HDL_TG | Dietary advice | 128 | -0.39 | (-0.00) | -0.81 | (-0.01) | 0.04 | (0.00) | 0.075 |
| S_LDL_TG | Supplement | 128 | -0.40 | (-0.00) | -0.85 | (-0.01) | 0.05 | (0.00) | 0.078 |
| ApoB | Supplement | 128 | -0.40 | (-0.07) | -0.84 | (-0.15) | 0.05 | (0.01) | 0.078 |
| MUFA_FA | Supplement | 128 | -0.37 | (-1.17) | -0.78 | (-2.49) | 0.04 | (0.14) | 0.079 |
| TotFA | Supplement | 128 | -0.38 | (-0.85) | -0.81 | (-1.80) | 0.05 | (0.10) | 0.080 |
| Remnant_C | Supplement | 128 | -0.39 | (-0.14) | -0.83 | (-0.29) | 0.05 | (0.02) | 0.081 |
| XS_VLDL_L | Supplement | 128 | -0.40 | (-0.04) | -0.85 | (-0.09) | 0.05 | (0.01) | 0.084 |
| Gp | Dietary advice | 128 | -0.38 | (-0.08) | -0.82 | (-0.16) | 0.06 | (0.01) | 0.091 |
| PUFA_FA | Dietary advice | 128 | 0.37 | (1.21) | -0.06 | (-0.20) | 0.81 | (2.63) | 0.092 |
| XL_HDL_TG | Supplement | 128 | -0.37 | (-0.00) | -0.81 | (-0.01) | 0.07 | (0.00) | 0.101 |
| S_HDL_TG | Dietary advice | 128 | -0.34 | (-0.00) | -0.75 | (-0.01) | 0.07 | (0.00) | 0.104 |
| XS_VLDL_C | Dietary advice | 128 | -0.33 | (-0.02) | -0.73 | (-0.04) | 0.07 | (0.00) | 0.106 |
| L_LDL_TG | Supplement | 128 | -0.38 | (-0.01) | -0.83 | (-0.02) | 0.08 | (0.00) | 0.106 |
| XS_VLDL_CE | Dietary advice | 128 | -0.33 | (-0.01) | -0.73 | (-0.03) | 0.08 | (0.00) | 0.111 |
| XS_VLDL_FC | Dietary advice | 128 | -0.32 | (-0.01) | -0.72 | (-0.01) | 0.08 | (0.00) | 0.111 |
| FAw3_FA | Supplement | 128 | -0.26 | (-0.32) | -0.57 | (-0.71) | 0.06 | (0.08) | 0.113 |
| FreeC | Dietary advice | 128 | -0.32 | (-0.09) | -0.72 | (-0.20) | 0.09 | (0.02) | 0.122 |
| S_LDL_PL | Dietary advice | 128 | -0.30 | (-0.01) | -0.67 | (-0.02) | 0.08 | (0.00) | 0.124 |
| HDL_TG | Supplement | 128 | -0.34 | (-0.01) | -0.78 | (-0.02) | 0.10 | (0.00) | 0.127 |
| Ace | Dietary advice | 128 | 0.26 | (0.00) | -0.08 | (-0.00) | 0.59 | (0.01) | 0.129 |
| Ala | Dietary advice | 128 | -0.32 | (-0.02) | -0.73 | (-0.05) | 0.09 | (0.01) | 0.130 |
| IDL_CE | Dietary advice | 128 | -0.31 | (-0.04) | -0.71 | (-0.09) | 0.09 | (0.01) | 0.133 |
| XS_VLDL_FC | Supplement | 128 | -0.34 | (-0.01) | -0.80 | (-0.01) | 0.11 | (0.00) | 0.135 |
| Cit | Supplement | 126 | 0.32 | (0.00) | -0.10 | (-0.00) | 0.74 | (0.01) | 0.137 |
| Alb | Dietary advice | 127 | -0.34 | (-0.00) | -0.79 | (-0.01) | 0.11 | (0.00) | 0.137 |
| IDL_P | Dietary advice | 128 | -0.30 | (-0.00) | -0.70 | (-0.00) | 0.10 | (0.00) | 0.140 |
| SFA_FA | Supplement | 128 | 0.32 | (0.61) | -0.11 | (-0.20) | 0.76 | (1.42) | 0.141 |
| M_LDL_PL | Dietary advice | 128 | -0.28 | (-0.01) | -0.67 | (-0.03) | 0.10 | (0.00) | 0.146 |
| S_HDL_TG | Supplement | 128 | -0.33 | (-0.00) | -0.79 | (-0.01) | 0.12 | (0.00) | 0.150 |
| sfa | Supplement | 128 | -0.31 | (-0.27) | -0.74 | (-0.64) | 0.12 | (0.10) | 0.150 |
| LDL_TG | Supplement | 128 | -0.32 | (-0.01) | -0.76 | (-0.03) | 0.12 | (0.00) | 0.153 |
| Tyr | Dietary advice | 128 | -0.29 | (-0.00) | -0.69 | (-0.01) | 0.11 | (0.00) | 0.154 |
| IDL_L | Dietary advice | 128 | -0.29 | (-0.08) | -0.68 | (-0.18) | 0.11 | (0.03) | 0.159 |
| XS_VLDL_C | Supplement | 128 | -0.32 | (-0.02) | -0.78 | (-0.04) | 0.13 | (0.01) | 0.161 |
| Serum_C | Dietary advice | 128 | -0.28 | (-0.27) | -0.67 | (-0.65) | 0.11 | (0.11) | 0.162 |
| IDL_C | Dietary advice | 128 | -0.28 | (-0.05) | -0.67 | (-0.12) | 0.12 | (0.02) | 0.175 |
| XS_VLDL_CE | Supplement | 128 | -0.31 | (-0.01) | -0.76 | (-0.03) | 0.15 | (0.01) | 0.182 |
| Gp | Supplement | 128 | -0.28 | (-0.05) | -0.69 | (-0.14) | 0.13 | (0.03) | 0.183 |
| M_HDL_TG | Supplement | 128 | -0.29 | (-0.00) | -0.74 | (-0.01) | 0.15 | (0.00) | 0.189 |
| pufa | Supplement | 128 | -0.29 | (-0.23) | -0.71 | (-0.57) | 0.14 | (0.11) | 0.189 |
| EstC | Dietary advice | 128 | -0.26 | (-0.18) | -0.65 | (-0.45) | 0.13 | (0.09) | 0.189 |
| FAw6_FA | Supplement | 128 | 0.28 | (0.88) | -0.14 | (-0.44) | 0.71 | (2.21) | 0.190 |
| M_LDL_TG | Supplement | 128 | -0.31 | (-0.00) | -0.77 | (-0.01) | 0.16 | (0.00) | 0.191 |
| Glol | Supplement | 128 | 0.29 | (0.01) | -0.15 | (-0.00) | 0.73 | (0.01) | 0.192 |
| S_LDL_FC | Dietary advice | 128 | -0.26 | (-0.00) | -0.65 | (-0.01) | 0.13 | (0.00) | 0.193 |
| VLDL_D | Supplement | 128 | -0.30 | (-0.35) | -0.75 | (-0.88) | 0.15 | (0.18) | 0.195 |
| Pyr | Dietary advice | 128 | -0.30 | (-0.01) | -0.75 | (-0.03) | 0.15 | (0.01) | 0.196 |
| HDL2_C | Supplement | 128 | 0.30 | (0.08) | -0.16 | (-0.04) | 0.77 | (0.21) | 0.197 |
| XS_VLDL_PL | Dietary advice | 128 | -0.26 | (-0.01) | -0.65 | (-0.02) | 0.14 | (0.01) | 0.198 |
| FALen | Supplement | 128 | -0.29 | (-0.13) | -0.73 | (-0.33) | 0.16 | (0.07) | 0.205 |
| L_LDL_P | Dietary advice | 128 | -0.25 | (-0.00) | -0.65 | (-0.00) | 0.15 | (0.00) | 0.214 |
| L_HDL_FC | Supplement | 128 | 0.29 | (0.01) | -0.17 | (-0.01) | 0.75 | (0.02) | 0.214 |
| FAw3_FA | Dietary advice | 128 | 0.30 | (0.37) | -0.18 | (-0.22) | 0.78 | (0.97) | 0.218 |
| FAw6_FA | Dietary advice | 128 | 0.27 | (0.84) | -0.16 | (-0.51) | 0.70 | (2.19) | 0.220 |
| Phe | Dietary advice | 128 | -0.24 | (-0.00) | -0.63 | (-0.01) | 0.15 | (0.00) | 0.220 |
| S_LDL_P | Dietary advice | 128 | -0.25 | (-0.00) | -0.64 | (-0.00) | 0.15 | (0.00) | 0.224 |
| sm | Supplement | 128 | -0.29 | (-0.02) | -0.75 | (-0.06) | 0.18 | (0.01) | 0.224 |
| L_LDL_L | Dietary advice | 128 | -0.24 | (-0.08) | -0.64 | (-0.21) | 0.16 | (0.05) | 0.235 |
| XL_HDL_PL | Supplement | 128 | 0.27 | (0.02) | -0.18 | (-0.02) | 0.73 | (0.07) | 0.237 |
| S_LDL_L | Dietary advice | 128 | -0.24 | (-0.03) | -0.64 | (-0.08) | 0.16 | (0.02) | 0.239 |
| M_LDL_FC | Dietary advice | 128 | -0.23 | (-0.01) | -0.62 | (-0.02) | 0.16 | (0.00) | 0.240 |
| L_HDL_C | Supplement | 128 | 0.27 | (0.04) | -0.19 | (-0.03) | 0.73 | (0.10) | 0.241 |
| L_LDL_CE | Dietary advice | 128 | -0.24 | (-0.04) | -0.64 | (-0.12) | 0.17 | (0.03) | 0.246 |
| M_LDL_P | Dietary advice | 128 | -0.23 | (-0.00) | -0.63 | (-0.00) | 0.17 | (0.00) | 0.247 |
| SFA_FA | Dietary advice | 128 | -0.23 | (-0.42) | -0.61 | (-1.14) | 0.16 | (0.30) | 0.249 |
| HDL_D | Supplement | 128 | 0.26 | (0.05) | -0.19 | (-0.04) | 0.71 | (0.13) | 0.250 |
| L_HDL_CE | Supplement | 128 | 0.27 | (0.03) | -0.19 | (-0.02) | 0.73 | (0.07) | 0.250 |
| Gln | Dietary advice | 128 | -0.24 | (-0.02) | -0.66 | (-0.04) | 0.18 | (0.01) | 0.252 |
| MUFA_FA | Dietary advice | 128 | -0.25 | (-0.79) | -0.68 | (-2.16) | 0.18 | (0.57) | 0.253 |
| L_LDL_PL | Dietary advice | 128 | -0.22 | (-0.02) | -0.61 | (-0.04) | 0.16 | (0.01) | 0.255 |
| M_LDL_L | Dietary advice | 128 | -0.23 | (-0.05) | -0.63 | (-0.13) | 0.17 | (0.03) | 0.259 |
| HDL_C | Supplement | 128 | 0.27 | (0.08) | -0.20 | (-0.06) | 0.73 | (0.22) | 0.259 |
| XS_VLDL_PL | Supplement | 128 | -0.26 | (-0.01) | -0.73 | (-0.03) | 0.20 | (0.01) | 0.261 |
| L_HDL_PL | Supplement | 128 | 0.26 | (0.03) | -0.20 | (-0.02) | 0.73 | (0.09) | 0.266 |
| L_HDL_L | Supplement | 128 | 0.26 | (0.07) | -0.20 | (-0.05) | 0.72 | (0.19) | 0.267 |
| L_LDL_C | Dietary advice | 128 | -0.22 | (-0.05) | -0.62 | (-0.15) | 0.18 | (0.04) | 0.276 |
| L_HDL_P | Supplement | 128 | 0.26 | (0.00) | -0.21 | (-0.00) | 0.72 | (0.00) | 0.276 |
| XL_HDL_FC | Supplement | 128 | 0.25 | (0.00) | -0.21 | (-0.00) | 0.71 | (0.01) | 0.280 |
| la | Supplement | 128 | -0.23 | (-0.15) | -0.66 | (-0.42) | 0.19 | (0.12) | 0.282 |
| Glc | Dietary advice | 128 | -0.24 | (-0.36) | -0.67 | (-1.02) | 0.20 | (0.30) | 0.282 |
| FAw6 | Supplement | 128 | -0.23 | (-0.17) | -0.66 | (-0.48) | 0.20 | (0.14) | 0.282 |
| IDL_PL | Dietary advice | 128 | -0.21 | (-0.01) | -0.61 | (-0.04) | 0.18 | (0.01) | 0.284 |
| CLA_FA | Dietary advice | 127 | -0.23 | (-0.03) | -0.66 | (-0.07) | 0.20 | (0.02) | 0.288 |
| ApoA1 | Dietary advice | 128 | -0.21 | (-0.04) | -0.59 | (-0.11) | 0.18 | (0.03) | 0.292 |
| HDL3_C | Dietary advice | 128 | -0.18 | (-0.01) | -0.52 | (-0.02) | 0.16 | (0.01) | 0.293 |
| sm | Dietary advice | 128 | -0.21 | (-0.02) | -0.60 | (-0.05) | 0.18 | (0.02) | 0.296 |
| LDL_C | Dietary advice | 128 | -0.21 | (-0.10) | -0.61 | (-0.30) | 0.19 | (0.10) | 0.306 |
| TotPG | Supplement | 128 | -0.24 | (-0.08) | -0.69 | (-0.24) | 0.22 | (0.08) | 0.307 |
| M_LDL_PL | Supplement | 128 | -0.23 | (-0.01) | -0.70 | (-0.03) | 0.23 | (0.01) | 0.327 |
| LA_FA | Supplement | 128 | 0.20 | (0.66) | -0.20 | (-0.67) | 0.60 | (1.99) | 0.328 |
| IDL_FC | Dietary advice | 128 | -0.20 | (-0.01) | -0.59 | (-0.03) | 0.20 | (0.01) | 0.331 |
| M_LDL_C | Dietary advice | 128 | -0.20 | (-0.03) | -0.61 | (-0.09) | 0.21 | (0.03) | 0.332 |
| pc | Supplement | 127 | -0.22 | (-0.08) | -0.68 | (-0.25) | 0.24 | (0.09) | 0.336 |
| IDL_P | Supplement | 128 | -0.22 | (-0.00) | -0.68 | (-0.00) | 0.24 | (0.00) | 0.343 |
| M_HDL_FC | Supplement | 128 | 0.22 | (0.00) | -0.24 | (-0.00) | 0.67 | (0.01) | 0.347 |
| M_HDL_C | Supplement | 128 | 0.22 | (0.02) | -0.24 | (-0.02) | 0.67 | (0.06) | 0.349 |
| M_HDL_CE | Supplement | 128 | 0.21 | (0.02) | -0.24 | (-0.02) | 0.67 | (0.05) | 0.351 |
| S_LDL_C | Dietary advice | 128 | -0.19 | (-0.02) | -0.60 | (-0.06) | 0.21 | (0.02) | 0.353 |
| S_LDL_PL | Supplement | 128 | -0.22 | (-0.01) | -0.68 | (-0.02) | 0.25 | (0.01) | 0.355 |
| M_LDL_CE | Dietary advice | 128 | -0.19 | (-0.02) | -0.60 | (-0.07) | 0.22 | (0.03) | 0.358 |
| TotCho | Supplement | 128 | -0.21 | (-0.08) | -0.67 | (-0.27) | 0.24 | (0.10) | 0.358 |
| Glc | Supplement | 128 | -0.20 | (-0.30) | -0.64 | (-0.98) | 0.25 | (0.38) | 0.378 |
| IDL_L | Supplement | 128 | -0.21 | (-0.05) | -0.67 | (-0.18) | 0.26 | (0.07) | 0.379 |
| M_HDL_PL | Supplement | 128 | 0.20 | (0.01) | -0.25 | (-0.02) | 0.65 | (0.05) | 0.381 |
| XL_HDL_L | Supplement | 128 | 0.20 | (0.03) | -0.25 | (-0.04) | 0.66 | (0.11) | 0.383 |
| L_LDL_FC | Dietary advice | 128 | -0.17 | (-0.01) | -0.57 | (-0.03) | 0.22 | (0.01) | 0.385 |
| IDL_CE | Supplement | 128 | -0.20 | (-0.03) | -0.66 | (-0.09) | 0.26 | (0.03) | 0.385 |
| XL_HDL_P | Supplement | 128 | 0.20 | (0.00) | -0.26 | (-0.00) | 0.65 | (0.00) | 0.394 |
| S_LDL_FC | Supplement | 128 | -0.20 | (-0.00) | -0.66 | (-0.01) | 0.26 | (0.01) | 0.397 |
| PUFA_FA | Supplement | 128 | 0.17 | (0.57) | -0.23 | (-0.75) | 0.58 | (1.88) | 0.397 |
| L_LDL_P | Supplement | 128 | -0.20 | (-0.00) | -0.66 | (-0.00) | 0.26 | (0.00) | 0.397 |
| M_LDL_P | Supplement | 128 | -0.20 | (-0.00) | -0.66 | (-0.00) | 0.26 | (0.00) | 0.399 |
| S_LDL_P | Supplement | 128 | -0.20 | (-0.00) | -0.65 | (-0.00) | 0.26 | (0.00) | 0.399 |
| S_LDL_CE | Dietary advice | 128 | -0.17 | (-0.01) | -0.58 | (-0.04) | 0.24 | (0.02) | 0.404 |
| M_HDL_L | Supplement | 128 | 0.19 | (0.03) | -0.26 | (-0.04) | 0.64 | (0.11) | 0.408 |
| M_LDL_L | Supplement | 128 | -0.19 | (-0.04) | -0.65 | (-0.13) | 0.27 | (0.05) | 0.410 |
| M_LDL_FC | Supplement | 128 | -0.19 | (-0.01) | -0.66 | (-0.02) | 0.27 | (0.01) | 0.413 |
| S_LDL_L | Supplement | 128 | -0.19 | (-0.02) | -0.65 | (-0.08) | 0.27 | (0.03) | 0.418 |
| XL_HDL_CE | Dietary advice | 128 | -0.17 | (-0.01) | -0.58 | (-0.03) | 0.24 | (0.01) | 0.418 |
| Lac | Dietary advice | 128 | -0.19 | (-0.12) | -0.65 | (-0.42) | 0.27 | (0.18) | 0.418 |
| L_LDL_L | Supplement | 128 | -0.19 | (-0.06) | -0.65 | (-0.21) | 0.27 | (0.09) | 0.423 |
| S_HDL_FC | Dietary advice | 128 | -0.17 | (-0.00) | -0.60 | (-0.01) | 0.25 | (0.00) | 0.428 |
| M_HDL_P | Supplement | 128 | 0.18 | (0.00) | -0.27 | (-0.00) | 0.64 | (0.00) | 0.429 |
| L_LDL_CE | Supplement | 128 | -0.18 | (-0.03) | -0.64 | (-0.12) | 0.28 | (0.05) | 0.434 |
| IDL_C | Supplement | 128 | -0.18 | (-0.03) | -0.64 | (-0.12) | 0.28 | (0.05) | 0.440 |
| L_LDL_PL | Supplement | 128 | -0.18 | (-0.01) | -0.65 | (-0.05) | 0.29 | (0.02) | 0.447 |
| Gly | Dietary advice | 128 | -0.16 | (-0.01) | -0.58 | (-0.02) | 0.26 | (0.01) | 0.457 |
| XL_HDL_C | Dietary advice | 128 | -0.15 | (-0.01) | -0.56 | (-0.04) | 0.26 | (0.02) | 0.466 |
| M_LDL_C | Supplement | 128 | -0.17 | (-0.03) | -0.63 | (-0.10) | 0.29 | (0.04) | 0.471 |
| L_LDL_C | Supplement | 128 | -0.17 | (-0.04) | -0.63 | (-0.15) | 0.29 | (0.07) | 0.471 |
| IDL_PL | Supplement | 128 | -0.17 | (-0.01) | -0.64 | (-0.04) | 0.30 | (0.02) | 0.474 |
| LDL_C | Supplement | 128 | -0.17 | (-0.08) | -0.63 | (-0.31) | 0.29 | (0.15) | 0.477 |
| S_HDL_PL | Dietary advice | 128 | -0.15 | (-0.01) | -0.58 | (-0.04) | 0.28 | (0.02) | 0.479 |
| S_HDL_CE | Supplement | 128 | 0.16 | (0.01) | -0.29 | (-0.02) | 0.61 | (0.04) | 0.486 |
| M_LDL_CE | Supplement | 128 | -0.16 | (-0.02) | -0.62 | (-0.08) | 0.30 | (0.04) | 0.488 |
| S_LDL_C | Supplement | 128 | -0.15 | (-0.01) | -0.61 | (-0.06) | 0.31 | (0.03) | 0.511 |
| FreeC | Supplement | 128 | -0.15 | (-0.04) | -0.61 | (-0.17) | 0.30 | (0.08) | 0.514 |
| S_HDL_C | Supplement | 128 | 0.15 | (0.01) | -0.30 | (-0.02) | 0.59 | (0.04) | 0.519 |
| FALen | Dietary advice | 128 | -0.13 | (-0.06) | -0.53 | (-0.24) | 0.27 | (0.12) | 0.525 |
| XL_HDL_C | Supplement | 128 | 0.15 | (0.01) | -0.31 | (-0.02) | 0.60 | (0.04) | 0.525 |
| Serum_C | Supplement | 128 | -0.15 | (-0.14) | -0.61 | (-0.59) | 0.32 | (0.30) | 0.534 |
| Glol | Dietary advice | 128 | 0.13 | (0.00) | -0.28 | (-0.01) | 0.54 | (0.01) | 0.536 |
| dha | Dietary advice | 128 | -0.15 | (-0.01) | -0.62 | (-0.04) | 0.32 | (0.02) | 0.537 |
| S_LDL_CE | Supplement | 128 | -0.14 | (-0.01) | -0.60 | (-0.05) | 0.32 | (0.02) | 0.546 |
| EstC | Supplement | 128 | -0.14 | (-0.10) | -0.61 | (-0.42) | 0.32 | (0.22) | 0.546 |
| DHA_FA | Dietary advice | 128 | 0.15 | (0.07) | -0.33 | (-0.15) | 0.62 | (0.29) | 0.547 |
| Gly | Supplement | 128 | 0.10 | (0.00) | -0.28 | (-0.01) | 0.49 | (0.02) | 0.594 |
| IDL_FC | Supplement | 128 | -0.13 | (-0.01) | -0.59 | (-0.03) | 0.34 | (0.02) | 0.596 |
| S_HDL_CE | Dietary advice | 128 | 0.11 | (0.01) | -0.29 | (-0.02) | 0.50 | (0.03) | 0.596 |
| L_LDL_FC | Supplement | 128 | -0.13 | (-0.01) | -0.59 | (-0.04) | 0.34 | (0.02) | 0.596 |
| Gln | Supplement | 128 | -0.11 | (-0.01) | -0.53 | (-0.03) | 0.31 | (0.02) | 0.603 |
| S_HDL_P | Dietary advice | 128 | -0.11 | (-0.00) | -0.52 | (-0.00) | 0.30 | (0.00) | 0.606 |
| XL_HDL_FC | Dietary advice | 128 | -0.11 | (-0.00) | -0.52 | (-0.01) | 0.31 | (0.01) | 0.613 |
| S_HDL_L | Dietary advice | 128 | -0.09 | (-0.01) | -0.50 | (-0.06) | 0.32 | (0.04) | 0.649 |
| XL_HDL_CE | Supplement | 128 | 0.11 | (0.01) | -0.35 | (-0.02) | 0.56 | (0.03) | 0.650 |
| LDL_D | Supplement | 128 | -0.11 | (-0.01) | -0.58 | (-0.08) | 0.36 | (0.05) | 0.654 |
| M_HDL_PL | Dietary advice | 128 | -0.09 | (-0.01) | -0.50 | (-0.04) | 0.32 | (0.02) | 0.666 |
| XL_HDL_P | Dietary advice | 128 | -0.09 | (-0.00) | -0.50 | (-0.00) | 0.32 | (0.00) | 0.675 |
| XL_HDL_L | Dietary advice | 128 | -0.09 | (-0.01) | -0.50 | (-0.08) | 0.32 | (0.05) | 0.676 |
| M_HDL_P | Dietary advice | 128 | -0.09 | (-0.00) | -0.49 | (-0.00) | 0.32 | (0.00) | 0.677 |
| FAw3 | Dietary advice | 128 | -0.09 | (-0.01) | -0.55 | (-0.09) | 0.37 | (0.06) | 0.708 |
| M_HDL_L | Dietary advice | 128 | -0.08 | (-0.01) | -0.48 | (-0.08) | 0.33 | (0.06) | 0.713 |
| ApoA1 | Supplement | 128 | 0.08 | (0.02) | -0.38 | (-0.07) | 0.55 | (0.11) | 0.719 |
| Alb | Supplement | 127 | -0.06 | (-0.00) | -0.42 | (-0.00) | 0.30 | (0.00) | 0.744 |
| S_HDL_C | Dietary advice | 128 | 0.06 | (0.00) | -0.33 | (-0.02) | 0.45 | (0.03) | 0.760 |
| Crea | Supplement | 128 | -0.07 | (-0.00) | -0.56 | (-0.01) | 0.41 | (0.01) | 0.772 |
| bOHBut | Dietary advice | 128 | 0.05 | (0.00) | -0.27 | (-0.01) | 0.36 | (0.01) | 0.773 |
| L_HDL_TG | Supplement | 128 | -0.07 | (-0.00) | -0.52 | (-0.00) | 0.39 | (0.00) | 0.779 |
| S_HDL_L | Supplement | 128 | 0.06 | (0.01) | -0.38 | (-0.05) | 0.51 | (0.06) | 0.780 |
| UnSat | Supplement | 128 | 0.05 | (0.00) | -0.32 | (-0.02) | 0.43 | (0.03) | 0.786 |
| LA_FA | Dietary advice | 128 | 0.06 | (0.20) | -0.38 | (-1.25) | 0.50 | (1.65) | 0.787 |
| HDL3_C | Supplement | 128 | -0.06 | (-0.00) | -0.55 | (-0.02) | 0.42 | (0.02) | 0.798 |
| S_HDL_P | Supplement | 128 | 0.05 | (0.00) | -0.39 | (-0.00) | 0.49 | (0.00) | 0.823 |
| M_HDL_FC | Dietary advice | 128 | -0.04 | (-0.00) | -0.44 | (-0.01) | 0.36 | (0.01) | 0.851 |
| LDL_D | Dietary advice | 128 | -0.03 | (-0.00) | -0.36 | (-0.05) | 0.30 | (0.04) | 0.865 |
| HDL_C | Dietary advice | 128 | -0.03 | (-0.01) | -0.42 | (-0.13) | 0.36 | (0.11) | 0.884 |
| L_HDL_P | Dietary advice | 128 | -0.03 | (-0.00) | -0.43 | (-0.00) | 0.38 | (0.00) | 0.896 |
| M_HDL_C | Dietary advice | 128 | -0.03 | (-0.00) | -0.42 | (-0.04) | 0.37 | (0.04) | 0.901 |
| M_HDL_CE | Dietary advice | 128 | -0.02 | (-0.00) | -0.42 | (-0.03) | 0.38 | (0.03) | 0.915 |
| L_HDL_L | Dietary advice | 128 | -0.02 | (-0.01) | -0.42 | (-0.11) | 0.38 | (0.10) | 0.921 |
| HDL_D | Dietary advice | 128 | 0.02 | (0.00) | -0.40 | (-0.08) | 0.44 | (0.08) | 0.927 |
| L_HDL_FC | Dietary advice | 128 | 0.02 | (0.00) | -0.39 | (-0.01) | 0.43 | (0.01) | 0.930 |
| S_HDL_PL | Supplement | 128 | 0.02 | (0.00) | -0.43 | (-0.03) | 0.46 | (0.04) | 0.934 |
| L_HDL_CE | Dietary advice | 128 | -0.02 | (-0.00) | -0.42 | (-0.04) | 0.39 | (0.04) | 0.942 |
| HDL2_C | Dietary advice | 128 | -0.01 | (-0.00) | -0.41 | (-0.11) | 0.39 | (0.10) | 0.967 |
| L_HDL_C | Dietary advice | 128 | -0.01 | (-0.00) | -0.41 | (-0.06) | 0.40 | (0.05) | 0.972 |
| L_HDL_PL | Dietary advice | 128 | -0.01 | (-0.00) | -0.41 | (-0.05) | 0.40 | (0.05) | 0.980 |
| S_HDL_FC | Supplement | 128 | 0.01 | (0.00) | -0.44 | (-0.01) | 0.45 | (0.01) | 0.981 |
| Cit | Dietary advice | 126 | 0.00 | (-0.00) | -0.41 | (-0.01) | 0.40 | (0.01) | 0.982 |
| XL_HDL_PL | Dietary advice | 128 | 0.00 | (0.00) | -0.41 | (-0.04) | 0.41 | (0.04) | 0.992 |

SD = standard deviation; CI=confidence interval.

*Per 1-SD in metabolite concentration.

*Non-standardised mean differences expressed in absolute concentration units (e.g. mmol/l metabolite difference between diet or supplement versus placebo).

For abbreviations of metabolic trait names, see Supplementary table 8.

**Supplementary table 5:** Comparison of unadjusted and adjusted regression results (n=116) for selected metabolic traits.

* Per 1-SD difference in metabolite concentrations. For abbreviations of metabolic trait names, see Supplementary table 8.

Unadjusted results differ slightly from those presented in the paper, as the sensitivity analyses only included individuals with baseline and follow-up metabolite data (n=116, rather than n=128).

**Supplementary Table 6:** Green tea unadjusted regression results

| **Metabolite** | **Intervention arm** | **N** | **Mean difference†**  **(concentration)*** | | **Lower CI**  **(concentration)*** | | **Upper CI**  **(concentration)*** | | **p-value** |
| --- | --- | --- | --- | --- | --- | --- | --- | --- | --- |
| PUFA_FA | Dietary advice | 128 | 0.66 | (2.15) | 0.27 | (0.89) | 1.05 | (3.41) | 0.001 |
| S_HDL_CE | Dietary advice | 128 | 0.62 | (0.04) | 0.19 | (0.01) | 1.04 | (0.06) | 0.005 |
| FAw6_FA | Dietary advice | 128 | 0.59 | (1.85) | 0.19 | (0.58) | 1.00 | (3.11) | 0.005 |
| Gly | Dietary advice | 128 | -0.58 | (-0.02) | -0.98 | (-0.04) | -0.18 | (-0.01) | 0.005 |
| MUFA_FA | Dietary advice | 128 | -0.58 | (-1.84) | -0.99 | (-3.14) | -0.17 | (-0.55) | 0.006 |
| S_HDL_C | Dietary advice | 128 | 0.56 | (0.04) | 0.13 | (0.01) | 0.98 | (0.07) | 0.011 |
| FALen | Dietary advice | 128 | -0.49 | (-0.22) | -0.89 | (-0.40) | -0.09 | (-0.04) | 0.017 |
| LA_FA | Dietary advice | 128 | 0.49 | (1.62) | 0.08 | (0.27) | 0.90 | (2.97) | 0.019 |
| L_LDL_FC | Dietary advice | 128 | 0.49 | (0.03) | 0.07 | (0.00) | 0.92 | (0.06) | 0.023 |
| S_LDL_CE | Dietary advice | 128 | 0.48 | (0.04) | 0.06 | (0.00) | 0.91 | (0.07) | 0.025 |
| VLDL_D | Dietary advice | 128 | -0.47 | (-0.56) | -0.88 | (-1.04) | -0.06 | (-0.07) | 0.026 |
| S_LDL_C | Dietary advice | 128 | 0.48 | (0.05) | 0.05 | (0.01) | 0.91 | (0.09) | 0.028 |
| IDL_FC | Dietary advice | 128 | 0.47 | (0.03) | 0.05 | (0.00) | 0.89 | (0.05) | 0.030 |
| M_LDL_CE | Dietary advice | 128 | 0.47 | (0.06) | 0.04 | (0.01) | 0.89 | (0.11) | 0.032 |
| M_LDL_C | Dietary advice | 128 | 0.47 | (0.07) | 0.04 | (0.01) | 0.89 | (0.14) | 0.032 |
| Crea | Dietary advice | 128 | -0.52 | (-0.01) | -0.99 | (-0.01) | -0.05 | (-0.00) | 0.032 |
| LDL_C | Dietary advice | 128 | 0.46 | (0.23) | 0.04 | (0.02) | 0.89 | (0.44) | 0.033 |
| S_HDL_TG | Dietary advice | 128 | -0.46 | (-0.01) | -0.89 | (-0.01) | -0.04 | (-0.00) | 0.033 |
| L_LDL_PL | Dietary advice | 128 | 0.47 | (0.03) | 0.04 | (0.00) | 0.90 | (0.06) | 0.034 |
| M_LDL_FC | Dietary advice | 128 | 0.47 | (0.01) | 0.03 | (0.00) | 0.91 | (0.03) | 0.036 |
| IDL_PL | Dietary advice | 128 | 0.46 | (0.03) | 0.03 | (0.00) | 0.89 | (0.06) | 0.036 |
| HDL3_C | Dietary advice | 128 | 0.50 | (0.02) | 0.03 | (0.00) | 0.96 | (0.03) | 0.037 |
| L_LDL_C | Dietary advice | 128 | 0.45 | (0.11) | 0.03 | (0.01) | 0.88 | (0.22) | 0.037 |
| M_LDL_L | Dietary advice | 128 | 0.46 | (0.09) | 0.03 | (0.01) | 0.89 | (0.18) | 0.038 |
| S_LDL_L | Dietary advice | 128 | 0.46 | (0.06) | 0.03 | (0.00) | 0.89 | (0.11) | 0.038 |
| M_LDL_P | Dietary advice | 128 | 0.45 | (0.00) | 0.02 | (0.00) | 0.88 | (0.00) | 0.039 |
| S_LDL_P | Dietary advice | 128 | 0.45 | (0.00) | 0.02 | (0.00) | 0.89 | (0.00) | 0.041 |
| L_LDL_L | Dietary advice | 128 | 0.45 | (0.15) | 0.02 | (0.01) | 0.88 | (0.29) | 0.041 |
| L_LDL_CE | Dietary advice | 128 | 0.44 | (0.08) | 0.01 | (0.00) | 0.86 | (0.16) | 0.044 |
| Crea | Supplement | 128 | -0.50 | (-0.01) | -0.98 | (-0.01) | -0.01 | (-0.00) | 0.044 |
| HDL_C | Dietary advice | 128 | 0.45 | (0.13) | 0.01 | (0.00) | 0.89 | (0.26) | 0.045 |
| EstC | Dietary advice | 128 | 0.44 | (0.31) | 0.01 | (0.01) | 0.88 | (0.61) | 0.045 |
| S_LDL_FC | Dietary advice | 128 | 0.45 | (0.01) | 0.01 | (0.00) | 0.89 | (0.02) | 0.045 |
| L_LDL_P | Dietary advice | 128 | 0.44 | (0.00) | 0.01 | (0.00) | 0.87 | (0.00) | 0.045 |
| Serum_C | Dietary advice | 128 | 0.44 | (0.42) | 0.00 | (0.00) | 0.87 | (0.84) | 0.049 |
| Ile | Dietary advice | 128 | -0.45 | (-0.01) | -0.91 | (-0.02) | 0.00 | (0.00) | 0.052 |
| S_HDL_TG | Supplement | 128 | -0.44 | (-0.01) | -0.89 | (-0.01) | 0.01 | (0.00) | 0.053 |
| HDL2_C | Dietary advice | 128 | 0.43 | (0.12) | -0.01 | (-0.00) | 0.87 | (0.23) | 0.054 |
| sm | Dietary advice | 128 | 0.43 | (0.04) | -0.01 | (-0.00) | 0.86 | (0.07) | 0.056 |
| PUFA_FA | Supplement | 128 | 0.43 | (1.38) | -0.01 | (-0.04) | 0.86 | (2.81) | 0.057 |
| M_LDL_PL | Dietary advice | 128 | 0.43 | (0.02) | -0.02 | (-0.00) | 0.87 | (0.04) | 0.060 |
| IDL_C | Dietary advice | 128 | 0.41 | (0.07) | -0.02 | (-0.00) | 0.83 | (0.15) | 0.061 |
| S_LDL_PL | Dietary advice | 128 | 0.43 | (0.01) | -0.02 | (-0.00) | 0.88 | (0.03) | 0.063 |
| IDL_L | Dietary advice | 128 | 0.40 | (0.11) | -0.03 | (-0.01) | 0.82 | (0.22) | 0.067 |
| L_HDL_PL | Dietary advice | 128 | 0.41 | (0.05) | -0.03 | (-0.00) | 0.85 | (0.10) | 0.067 |
| FreeC | Dietary advice | 128 | 0.40 | (0.11) | -0.03 | (-0.01) | 0.84 | (0.23) | 0.069 |
| Glc | Supplement | 128 | 0.44 | (0.67) | -0.04 | (-0.05) | 0.91 | (1.39) | 0.070 |
| XS_VLDL_PL | Dietary advice | 128 | 0.40 | (0.01) | -0.04 | (-0.00) | 0.83 | (0.03) | 0.071 |
| UnSat | Supplement | 128 | 0.36 | (0.03) | -0.04 | (-0.00) | 0.75 | (0.06) | 0.075 |
| XL_VLDL_TG | Dietary advice | 128 | -0.38 | (-0.01) | -0.81 | (-0.03) | 0.04 | (0.00) | 0.077 |
| ApoA1 | Dietary advice | 128 | 0.40 | (0.08) | -0.04 | (-0.01) | 0.85 | (0.16) | 0.077 |
| Leu | Dietary advice | 128 | -0.40 | (-0.01) | -0.85 | (-0.02) | 0.04 | (0.00) | 0.077 |
| IDL_P | Dietary advice | 128 | 0.38 | (0.00) | -0.04 | (-0.00) | 0.81 | (0.00) | 0.078 |
| Gp | Dietary advice | 128 | -0.38 | (-0.07) | -0.80 | (-0.16) | 0.05 | (0.01) | 0.080 |
| IDL_CE | Dietary advice | 128 | 0.38 | (0.05) | -0.05 | (-0.01) | 0.81 | (0.11) | 0.082 |
| XL_VLDL_P | Dietary advice | 128 | -0.38 | (-0.00) | -0.81 | (-0.00) | 0.05 | (0.00) | 0.083 |
| XL_VLDL_L | Dietary advice | 128 | -0.38 | (-0.02) | -0.80 | (-0.04) | 0.05 | (0.00) | 0.085 |
| L_VLDL_TG | Dietary advice | 128 | -0.37 | (-0.04) | -0.80 | (-0.08) | 0.06 | (0.01) | 0.092 |
| XL_VLDL_CE | Dietary advice | 128 | -0.37 | (-0.00) | -0.80 | (-0.01) | 0.06 | (0.00) | 0.093 |
| VLDL_TG | Dietary advice | 128 | -0.37 | (-0.14) | -0.79 | (-0.30) | 0.06 | (0.02) | 0.094 |
| M_HDL_FC | Dietary advice | 128 | 0.37 | (0.01) | -0.07 | (-0.00) | 0.81 | (0.02) | 0.094 |
| L_VLDL_FC | Dietary advice | 128 | -0.36 | (-0.01) | -0.79 | (-0.02) | 0.06 | (0.00) | 0.095 |
| M_HDL_C | Dietary advice | 128 | 0.37 | (0.03) | -0.07 | (-0.01) | 0.81 | (0.08) | 0.096 |
| L_VLDL_P | Dietary advice | 128 | -0.36 | (-0.00) | -0.79 | (-0.00) | 0.07 | (0.00) | 0.098 |
| M_HDL_CE | Dietary advice | 128 | 0.37 | (0.03) | -0.07 | (-0.01) | 0.81 | (0.06) | 0.098 |
| L_VLDL_L | Dietary advice | 128 | -0.36 | (-0.06) | -0.79 | (-0.14) | 0.07 | (0.01) | 0.099 |
| HDL_D | Dietary advice | 128 | 0.35 | (0.07) | -0.07 | (-0.01) | 0.77 | (0.15) | 0.099 |
| XL_VLDL_C | Dietary advice | 128 | -0.36 | (-0.00) | -0.79 | (-0.01) | 0.07 | (0.00) | 0.100 |
| L_HDL_L | Dietary advice | 128 | 0.36 | (0.10) | -0.08 | (-0.02) | 0.80 | (0.21) | 0.104 |
| L_HDL_P | Dietary advice | 128 | 0.36 | (0.00) | -0.08 | (-0.00) | 0.80 | (0.00) | 0.104 |
| L_VLDL_C | Dietary advice | 128 | -0.35 | (-0.01) | -0.78 | (-0.03) | 0.08 | (0.00) | 0.109 |
| XL_VLDL_PL | Dietary advice | 128 | -0.35 | (-0.00) | -0.78 | (-0.01) | 0.08 | (0.00) | 0.109 |
| XL_VLDL_FC | Dietary advice | 128 | -0.35 | (-0.00) | -0.78 | (-0.00) | 0.08 | (0.00) | 0.111 |
| MUFA_FA | Supplement | 128 | -0.36 | (-1.14) | -0.80 | (-2.55) | 0.08 | (0.27) | 0.111 |
| L_HDL_FC | Dietary advice | 128 | 0.35 | (0.01) | -0.09 | (-0.00) | 0.79 | (0.03) | 0.114 |
| L_VLDL_PL | Dietary advice | 128 | -0.35 | (-0.01) | -0.78 | (-0.02) | 0.08 | (0.00) | 0.114 |
| XXL_VLDL_FC | Dietary advice | 128 | -0.34 | (-0.00) | -0.78 | (-0.00) | 0.09 | (0.00) | 0.117 |
| XL_HDL_PL | Dietary advice | 128 | 0.34 | (0.03) | -0.09 | (-0.01) | 0.76 | (0.07) | 0.119 |
| L_VLDL_CE | Dietary advice | 128 | -0.34 | (-0.01) | -0.77 | (-0.02) | 0.10 | (0.00) | 0.128 |
| XXL_VLDL_C | Dietary advice | 128 | -0.34 | (-0.00) | -0.77 | (-0.00) | 0.10 | (0.00) | 0.129 |
| Serum_TG | Dietary advice | 128 | -0.33 | (-0.15) | -0.76 | (-0.34) | 0.10 | (0.04) | 0.130 |
| FAw3_FA | Supplement | 128 | 0.30 | (0.38) | -0.09 | (-0.11) | 0.70 | (0.87) | 0.130 |
| L_HDL_C | Dietary advice | 128 | 0.34 | (0.05) | -0.10 | (-0.01) | 0.78 | (0.11) | 0.131 |
| XS_VLDL_FC | Dietary advice | 128 | 0.33 | (0.01) | -0.10 | (-0.00) | 0.76 | (0.01) | 0.134 |
| L_HDL_CE | Dietary advice | 128 | 0.33 | (0.03) | -0.11 | (-0.01) | 0.77 | (0.08) | 0.137 |
| HDL_D | Supplement | 128 | 0.34 | (0.06) | -0.11 | (-0.02) | 0.78 | (0.15) | 0.137 |
| L_HDL_CE | Supplement | 128 | 0.32 | (0.03) | -0.12 | (-0.01) | 0.76 | (0.08) | 0.147 |
| XXL_VLDL_CE | Dietary advice | 128 | -0.32 | (-0.00) | -0.76 | (-0.00) | 0.12 | (0.00) | 0.148 |
| FAw6_FA | Supplement | 128 | 0.32 | (1.01) | -0.12 | (-0.36) | 0.76 | (2.37) | 0.148 |
| M_HDL_L | Dietary advice | 128 | 0.32 | (0.05) | -0.12 | (-0.02) | 0.76 | (0.13) | 0.149 |
| L_HDL_C | Supplement | 128 | 0.32 | (0.04) | -0.12 | (-0.02) | 0.76 | (0.10) | 0.150 |
| L_HDL_PL | Supplement | 128 | 0.32 | (0.04) | -0.12 | (-0.01) | 0.77 | (0.09) | 0.152 |
| VLDL_TG | Supplement | 128 | -0.33 | (-0.13) | -0.79 | (-0.30) | 0.13 | (0.05) | 0.153 |
| L_HDL_L | Supplement | 128 | 0.32 | (0.08) | -0.12 | (-0.03) | 0.76 | (0.20) | 0.155 |
| L_HDL_P | Supplement | 128 | 0.32 | (0.00) | -0.12 | (-0.00) | 0.76 | (0.00) | 0.157 |
| Serum_TG | Supplement | 128 | -0.33 | (-0.15) | -0.78 | (-0.35) | 0.13 | (0.06) | 0.158 |
| VLDL_D | Supplement | 128 | -0.32 | (-0.38) | -0.77 | (-0.91) | 0.13 | (0.15) | 0.159 |
| Phe | Dietary advice | 128 | -0.31 | (-0.00) | -0.75 | (-0.01) | 0.12 | (0.00) | 0.159 |
| L_HDL_FC | Supplement | 128 | 0.31 | (0.01) | -0.12 | (-0.00) | 0.75 | (0.02) | 0.159 |
| HDL_TG | Dietary advice | 128 | -0.31 | (-0.01) | -0.74 | (-0.02) | 0.13 | (0.00) | 0.162 |
| M_HDL_P | Dietary advice | 128 | 0.31 | (0.00) | -0.13 | (-0.00) | 0.75 | (0.00) | 0.163 |
| Gly | Supplement | 128 | -0.32 | (-0.01) | -0.79 | (-0.03) | 0.14 | (0.01) | 0.172 |
| DHA_FA | Supplement | 128 | 0.28 | (0.13) | -0.13 | (-0.06) | 0.69 | (0.32) | 0.173 |
| XXL_VLDL_L | Dietary advice | 128 | -0.30 | (-0.01) | -0.74 | (-0.02) | 0.13 | (0.00) | 0.174 |
| UnSat | Dietary advice | 128 | 0.31 | (0.02) | -0.14 | (-0.01) | 0.76 | (0.06) | 0.175 |
| XL_VLDL_TG | Supplement | 128 | -0.31 | (-0.01) | -0.77 | (-0.03) | 0.14 | (0.00) | 0.176 |
| pufa | Dietary advice | 128 | 0.31 | (0.25) | -0.14 | (-0.11) | 0.77 | (0.62) | 0.177 |
| XXL_VLDL_PL | Dietary advice | 128 | -0.30 | (-0.00) | -0.73 | (-0.00) | 0.14 | (0.00) | 0.179 |
| XXL_VLDL_P | Dietary advice | 128 | -0.30 | (-0.00) | -0.73 | (-0.00) | 0.14 | (0.00) | 0.179 |
| FAw6 | Dietary advice | 128 | 0.31 | (0.22) | -0.15 | (-0.11) | 0.76 | (0.55) | 0.182 |
| M_VLDL_TG | Dietary advice | 128 | -0.30 | (-0.04) | -0.73 | (-0.10) | 0.14 | (0.02) | 0.182 |
| L_VLDL_TG | Supplement | 128 | -0.31 | (-0.03) | -0.77 | (-0.08) | 0.15 | (0.02) | 0.183 |
| M_HDL_PL | Dietary advice | 128 | 0.30 | (0.02) | -0.14 | (-0.01) | 0.74 | (0.06) | 0.183 |
| XS_VLDL_C | Dietary advice | 128 | 0.29 | (0.02) | -0.14 | (-0.01) | 0.72 | (0.04) | 0.185 |
| L_VLDL_P | Supplement | 128 | -0.31 | (-0.00) | -0.77 | (-0.00) | 0.15 | (0.00) | 0.187 |
| XL_VLDL_P | Supplement | 128 | -0.31 | (-0.00) | -0.77 | (-0.00) | 0.15 | (0.00) | 0.188 |
| XXL_VLDL_TG | Dietary advice | 128 | -0.29 | (-0.00) | -0.73 | (-0.01) | 0.14 | (0.00) | 0.189 |
| L_VLDL_L | Supplement | 128 | -0.31 | (-0.05) | -0.76 | (-0.14) | 0.15 | (0.03) | 0.189 |
| HDL_C | Supplement | 128 | 0.30 | (0.09) | -0.15 | (-0.05) | 0.75 | (0.22) | 0.191 |
| L_VLDL_PL | Supplement | 128 | -0.30 | (-0.01) | -0.76 | (-0.02) | 0.15 | (0.00) | 0.192 |
| XL_VLDL_L | Supplement | 128 | -0.30 | (-0.02) | -0.76 | (-0.04) | 0.15 | (0.01) | 0.192 |
| M_VLDL_FC | Dietary advice | 128 | -0.29 | (-0.01) | -0.73 | (-0.02) | 0.15 | (0.00) | 0.193 |
| la | Dietary advice | 128 | 0.30 | (0.19) | -0.16 | (-0.10) | 0.75 | (0.48) | 0.196 |
| HDL2_C | Supplement | 128 | 0.30 | (0.08) | -0.16 | (-0.04) | 0.75 | (0.20) | 0.197 |
| L_VLDL_FC | Supplement | 128 | -0.30 | (-0.01) | -0.76 | (-0.02) | 0.16 | (0.00) | 0.197 |
| bOHBut | Dietary advice | 128 | 0.29 | (0.01) | -0.16 | (-0.01) | 0.74 | (0.03) | 0.198 |
| XL_VLDL_PL | Supplement | 128 | -0.29 | (-0.00) | -0.75 | (-0.01) | 0.16 | (0.00) | 0.207 |
| L_VLDL_C | Supplement | 128 | -0.30 | (-0.01) | -0.76 | (-0.03) | 0.17 | (0.01) | 0.207 |
| Ace | Supplement | 128 | -0.28 | (-0.00) | -0.71 | (-0.01) | 0.16 | (0.00) | 0.210 |
| XL_HDL_P | Dietary advice | 128 | 0.27 | (0.00) | -0.16 | (-0.00) | 0.70 | (0.00) | 0.211 |
| XL_HDL_L | Dietary advice | 128 | 0.27 | (0.04) | -0.16 | (-0.03) | 0.70 | (0.11) | 0.215 |
| His | Dietary advice | 128 | 0.27 | (0.00) | -0.16 | (-0.00) | 0.70 | (0.01) | 0.217 |
| M_VLDL_P | Dietary advice | 128 | -0.27 | (-0.00) | -0.71 | (-0.00) | 0.17 | (0.00) | 0.220 |
| L_VLDL_CE | Supplement | 128 | -0.29 | (-0.01) | -0.75 | (-0.02) | 0.18 | (0.00) | 0.222 |
| XS_VLDL_CE | Dietary advice | 128 | 0.26 | (0.01) | -0.16 | (-0.01) | 0.69 | (0.03) | 0.224 |
| Ala | Dietary advice | 128 | -0.28 | (-0.02) | -0.73 | (-0.05) | 0.17 | (0.01) | 0.225 |
| M_VLDL_L | Dietary advice | 128 | -0.27 | (-0.07) | -0.71 | (-0.18) | 0.17 | (0.04) | 0.226 |
| XL_VLDL_C | Supplement | 128 | -0.27 | (-0.00) | -0.74 | (-0.01) | 0.19 | (0.00) | 0.243 |
| XL_VLDL_FC | Supplement | 128 | -0.27 | (-0.00) | -0.74 | (-0.00) | 0.19 | (0.00) | 0.244 |
| XL_VLDL_CE | Supplement | 128 | -0.27 | (-0.00) | -0.74 | (-0.00) | 0.19 | (0.00) | 0.244 |
| HDL_TG | Supplement | 128 | -0.26 | (-0.01) | -0.70 | (-0.02) | 0.18 | (0.01) | 0.247 |
| CLA_FA | Dietary advice | 127 | -0.25 | (-0.03) | -0.67 | (-0.07) | 0.18 | (0.02) | 0.257 |
| HDL3_C | Supplement | 128 | 0.28 | (0.01) | -0.21 | (-0.01) | 0.76 | (0.03) | 0.261 |
| TotPG | Dietary advice | 128 | 0.26 | (0.09) | -0.19 | (-0.07) | 0.71 | (0.24) | 0.263 |
| XL_HDL_PL | Supplement | 128 | 0.25 | (0.02) | -0.19 | (-0.02) | 0.69 | (0.06) | 0.263 |
| S_VLDL_TG | Dietary advice | 128 | -0.25 | (-0.02) | -0.70 | (-0.05) | 0.19 | (0.01) | 0.265 |
| XXL_VLDL_FC | Supplement | 128 | -0.26 | (-0.00) | -0.73 | (-0.00) | 0.20 | (0.00) | 0.267 |
| M_VLDL_PL | Dietary advice | 128 | -0.25 | (-0.01) | -0.69 | (-0.03) | 0.19 | (0.01) | 0.267 |
| M_HDL_CE | Supplement | 128 | 0.26 | (0.02) | -0.20 | (-0.01) | 0.72 | (0.05) | 0.267 |
| M_VLDL_FC | Supplement | 128 | -0.26 | (-0.01) | -0.73 | (-0.02) | 0.21 | (0.01) | 0.271 |
| XS_VLDL_L | Dietary advice | 128 | 0.25 | (0.03) | -0.19 | (-0.02) | 0.69 | (0.07) | 0.271 |
| Glol | Dietary advice | 128 | 0.22 | (0.00) | -0.18 | (-0.00) | 0.61 | (0.01) | 0.278 |
| M_HDL_C | Supplement | 128 | 0.25 | (0.02) | -0.21 | (-0.02) | 0.71 | (0.07) | 0.280 |
| M_VLDL_TG | Supplement | 128 | -0.25 | (-0.04) | -0.72 | (-0.10) | 0.21 | (0.03) | 0.281 |
| XXL_VLDL_C | Supplement | 128 | -0.25 | (-0.00) | -0.72 | (-0.00) | 0.21 | (0.00) | 0.283 |
| S_VLDL_TG | Supplement | 128 | -0.25 | (-0.02) | -0.72 | (-0.05) | 0.22 | (0.02) | 0.287 |
| S_HDL_L | Dietary advice | 128 | 0.23 | (0.03) | -0.20 | (-0.02) | 0.66 | (0.08) | 0.290 |
| M_VLDL_P | Supplement | 128 | -0.25 | (-0.00) | -0.72 | (-0.00) | 0.22 | (0.00) | 0.291 |
| Val | Dietary advice | 128 | -0.24 | (-0.01) | -0.68 | (-0.02) | 0.21 | (0.01) | 0.291 |
| M_VLDL_L | Supplement | 128 | -0.25 | (-0.06) | -0.72 | (-0.18) | 0.22 | (0.06) | 0.292 |
| FALen | Supplement | 128 | -0.24 | (-0.11) | -0.69 | (-0.31) | 0.21 | (0.09) | 0.293 |
| TotCho | Dietary advice | 128 | 0.24 | (0.10) | -0.21 | (-0.08) | 0.69 | (0.28) | 0.294 |
| FAw3_FA | Dietary advice | 128 | 0.24 | (0.30) | -0.21 | (-0.26) | 0.69 | (0.86) | 0.295 |
| XS_VLDL_TG | Supplement | 128 | -0.25 | (-0.01) | -0.72 | (-0.02) | 0.22 | (0.01) | 0.298 |
| Pyr | Supplement | 128 | 0.22 | (0.01) | -0.20 | (-0.01) | 0.65 | (0.03) | 0.298 |
| Gp | Supplement | 128 | -0.24 | (-0.05) | -0.70 | (-0.14) | 0.22 | (0.04) | 0.299 |
| XXL_VLDL_PL | Supplement | 128 | -0.24 | (-0.00) | -0.71 | (-0.00) | 0.22 | (0.00) | 0.304 |
| M_VLDL_PL | Supplement | 128 | -0.24 | (-0.01) | -0.71 | (-0.03) | 0.22 | (0.01) | 0.305 |
| M_HDL_TG | Supplement | 128 | -0.22 | (-0.00) | -0.66 | (-0.01) | 0.21 | (0.00) | 0.305 |
| XXL_VLDL_CE | Supplement | 128 | -0.24 | (-0.00) | -0.71 | (-0.00) | 0.22 | (0.00) | 0.308 |
| ApoA1 | Supplement | 128 | 0.24 | (0.05) | -0.22 | (-0.04) | 0.70 | (0.14) | 0.311 |
| DHA_FA | Dietary advice | 128 | 0.24 | (0.11) | -0.22 | (-0.10) | 0.70 | (0.32) | 0.313 |
| M_VLDL_C | Dietary advice | 128 | -0.22 | (-0.02) | -0.67 | (-0.05) | 0.22 | (0.01) | 0.316 |
| M_VLDL_C | Supplement | 128 | -0.24 | (-0.02) | -0.70 | (-0.05) | 0.23 | (0.02) | 0.320 |
| XXL_VLDL_L | Supplement | 128 | -0.23 | (-0.00) | -0.70 | (-0.01) | 0.23 | (0.00) | 0.320 |
| mufa | Supplement | 128 | -0.23 | (-0.17) | -0.68 | (-0.51) | 0.23 | (0.17) | 0.323 |
| XXL_VLDL_P | Supplement | 128 | -0.23 | (-0.00) | -0.70 | (-0.00) | 0.23 | (0.00) | 0.325 |
| XL_HDL_FC | Dietary advice | 128 | 0.22 | (0.00) | -0.22 | (-0.00) | 0.65 | (0.01) | 0.328 |
| mufa | Dietary advice | 128 | -0.22 | (-0.16) | -0.66 | (-0.49) | 0.23 | (0.17) | 0.334 |
| XXL_VLDL_TG | Supplement | 128 | -0.23 | (-0.00) | -0.69 | (-0.01) | 0.24 | (0.00) | 0.335 |
| M_HDL_FC | Supplement | 128 | 0.22 | (0.00) | -0.23 | (-0.00) | 0.68 | (0.01) | 0.336 |
| XS_VLDL_P | Dietary advice | 128 | 0.22 | (0.00) | -0.23 | (-0.00) | 0.66 | (0.00) | 0.337 |
| S_HDL_CE | Supplement | 128 | 0.22 | (0.01) | -0.24 | (-0.01) | 0.67 | (0.04) | 0.347 |
| S_VLDL_P | Supplement | 128 | -0.22 | (-0.00) | -0.69 | (-0.00) | 0.25 | (0.00) | 0.348 |
| S_HDL_P | Dietary advice | 128 | 0.20 | (0.00) | -0.23 | (-0.00) | 0.63 | (0.00) | 0.350 |
| XL_HDL_TG | Dietary advice | 128 | -0.20 | (-0.00) | -0.63 | (-0.00) | 0.23 | (0.00) | 0.351 |
| XL_HDL_C | Dietary advice | 128 | 0.20 | (0.01) | -0.23 | (-0.02) | 0.63 | (0.04) | 0.359 |
| Cit | Supplement | 126 | 0.20 | (0.00) | -0.24 | (-0.00) | 0.65 | (0.01) | 0.362 |
| S_VLDL_FC | Supplement | 128 | -0.22 | (-0.00) | -0.68 | (-0.01) | 0.25 | (0.01) | 0.362 |
| S_VLDL_L | Supplement | 128 | -0.22 | (-0.03) | -0.69 | (-0.11) | 0.25 | (0.04) | 0.363 |
| M_LDL_TG | Dietary advice | 128 | 0.21 | (0.00) | -0.25 | (-0.00) | 0.66 | (0.01) | 0.364 |
| M_HDL_TG | Dietary advice | 128 | -0.20 | (-0.00) | -0.65 | (-0.01) | 0.24 | (0.00) | 0.368 |
| XL_HDL_CE | Dietary advice | 128 | 0.19 | (0.01) | -0.24 | (-0.01) | 0.62 | (0.03) | 0.377 |
| S_VLDL_PL | Supplement | 128 | -0.21 | (-0.01) | -0.67 | (-0.02) | 0.26 | (0.01) | 0.385 |
| M_VLDL_CE | Supplement | 128 | -0.21 | (-0.01) | -0.68 | (-0.03) | 0.26 | (0.01) | 0.387 |
| M_HDL_L | Supplement | 128 | 0.20 | (0.03) | -0.26 | (-0.04) | 0.65 | (0.11) | 0.388 |
| Phe | Supplement | 128 | -0.19 | (-0.00) | -0.64 | (-0.01) | 0.26 | (0.00) | 0.397 |
| XL_HDL_P | Supplement | 128 | 0.19 | (0.00) | -0.25 | (-0.00) | 0.63 | (0.00) | 0.398 |
| ApoB | Dietary advice | 128 | 0.19 | (0.03) | -0.26 | (-0.05) | 0.64 | (0.11) | 0.403 |
| dha | Dietary advice | 128 | 0.20 | (0.01) | -0.27 | (-0.02) | 0.67 | (0.04) | 0.404 |
| VLDL_C | Supplement | 128 | -0.20 | (-0.04) | -0.67 | (-0.14) | 0.27 | (0.06) | 0.404 |
| XL_HDL_L | Supplement | 128 | 0.18 | (0.03) | -0.25 | (-0.04) | 0.62 | (0.10) | 0.408 |
| M_HDL_P | Supplement | 128 | 0.19 | (0.00) | -0.26 | (-0.00) | 0.64 | (0.00) | 0.410 |
| S_HDL_C | Supplement | 128 | 0.19 | (0.01) | -0.27 | (-0.02) | 0.65 | (0.04) | 0.411 |
| Ile | Supplement | 128 | -0.18 | (-0.00) | -0.61 | (-0.01) | 0.26 | (0.00) | 0.418 |
| sm | Supplement | 128 | 0.19 | (0.02) | -0.27 | (-0.02) | 0.64 | (0.05) | 0.422 |
| L_LDL_FC | Supplement | 128 | 0.19 | (0.01) | -0.28 | (-0.02) | 0.66 | (0.04) | 0.430 |
| FAw3 | Dietary advice | 128 | 0.18 | (0.03) | -0.28 | (-0.04) | 0.65 | (0.10) | 0.441 |
| cla | Dietary advice | 127 | -0.17 | (-0.00) | -0.60 | (-0.01) | 0.26 | (0.00) | 0.444 |
| dha | Supplement | 128 | 0.16 | (0.01) | -0.26 | (-0.01) | 0.58 | (0.03) | 0.448 |
| IDL_FC | Supplement | 128 | 0.18 | (0.01) | -0.29 | (-0.02) | 0.65 | (0.03) | 0.459 |
| M_HDL_PL | Supplement | 128 | 0.16 | (0.01) | -0.28 | (-0.02) | 0.61 | (0.05) | 0.468 |
| XS_VLDL_TG | Dietary advice | 128 | -0.17 | (-0.00) | -0.62 | (-0.02) | 0.29 | (0.01) | 0.469 |
| Remnant_C | Dietary advice | 128 | 0.16 | (0.06) | -0.28 | (-0.10) | 0.60 | (0.21) | 0.473 |
| FAw3 | Supplement | 128 | 0.15 | (0.02) | -0.26 | (-0.04) | 0.56 | (0.09) | 0.474 |
| M_VLDL_CE | Dietary advice | 128 | -0.16 | (-0.01) | -0.60 | (-0.02) | 0.28 | (0.01) | 0.475 |
| pc | Dietary advice | 127 | 0.17 | (0.06) | -0.29 | (-0.11) | 0.62 | (0.23) | 0.478 |
| L_LDL_PL | Supplement | 128 | 0.16 | (0.01) | -0.31 | (-0.02) | 0.64 | (0.05) | 0.494 |
| LDL_TG | Supplement | 128 | -0.15 | (-0.01) | -0.60 | (-0.02) | 0.29 | (0.01) | 0.496 |
| EstC | Supplement | 128 | 0.16 | (0.11) | -0.31 | (-0.22) | 0.63 | (0.44) | 0.497 |
| S_VLDL_CE | Dietary advice | 128 | 0.15 | (0.01) | -0.29 | (-0.01) | 0.59 | (0.02) | 0.499 |
| LA_FA | Supplement | 128 | 0.15 | (0.49) | -0.29 | (-0.95) | 0.58 | (1.92) | 0.503 |
| IDL_PL | Supplement | 128 | 0.16 | (0.01) | -0.31 | (-0.02) | 0.64 | (0.04) | 0.504 |
| SFA_FA | Dietary advice | 128 | -0.16 | (-0.30) | -0.64 | (-1.20) | 0.32 | (0.59) | 0.507 |
| Glol | Supplement | 128 | 0.16 | (0.00) | -0.32 | (-0.01) | 0.64 | (0.01) | 0.509 |
| IDL_TG | Supplement | 128 | -0.16 | (-0.00) | -0.63 | (-0.01) | 0.31 | (0.01) | 0.512 |
| S_LDL_CE | Supplement | 128 | 0.15 | (0.01) | -0.31 | (-0.02) | 0.61 | (0.05) | 0.520 |
| LDL_D | Dietary advice | 128 | -0.16 | (-0.02) | -0.64 | (-0.09) | 0.33 | (0.05) | 0.526 |
| Leu | Supplement | 128 | -0.14 | (-0.00) | -0.58 | (-0.01) | 0.30 | (0.01) | 0.528 |
| S_LDL_C | Supplement | 128 | 0.15 | (0.01) | -0.31 | (-0.03) | 0.61 | (0.06) | 0.530 |
| M_LDL_FC | Supplement | 128 | 0.15 | (0.00) | -0.32 | (-0.01) | 0.62 | (0.02) | 0.531 |
| Serum_C | Supplement | 128 | 0.15 | (0.14) | -0.32 | (-0.31) | 0.62 | (0.60) | 0.532 |
| L_LDL_C | Supplement | 128 | 0.15 | (0.04) | -0.32 | (-0.08) | 0.62 | (0.15) | 0.533 |
| LDL_C | Supplement | 128 | 0.15 | (0.07) | -0.32 | (-0.16) | 0.61 | (0.30) | 0.535 |
| XL_HDL_FC | Supplement | 128 | 0.14 | (0.00) | -0.30 | (-0.01) | 0.57 | (0.01) | 0.538 |
| M_LDL_C | Supplement | 128 | 0.14 | (0.02) | -0.32 | (-0.05) | 0.60 | (0.09) | 0.543 |
| Pyr | Dietary advice | 128 | -0.14 | (-0.01) | -0.58 | (-0.02) | 0.31 | (0.01) | 0.544 |
| M_LDL_CE | Supplement | 128 | 0.14 | (0.02) | -0.32 | (-0.04) | 0.60 | (0.07) | 0.547 |
| S_VLDL_P | Dietary advice | 128 | -0.14 | (-0.00) | -0.59 | (-0.00) | 0.31 | (0.00) | 0.550 |
| L_LDL_TG | Dietary advice | 128 | 0.14 | (0.00) | -0.32 | (-0.01) | 0.59 | (0.01) | 0.553 |
| CLA_FA | Supplement | 127 | -0.13 | (-0.01) | -0.57 | (-0.06) | 0.31 | (0.03) | 0.554 |
| L_LDL_L | Supplement | 128 | 0.14 | (0.05) | -0.33 | (-0.11) | 0.61 | (0.20) | 0.557 |
| TotPG | Supplement | 128 | 0.13 | (0.05) | -0.32 | (-0.11) | 0.58 | (0.20) | 0.558 |
| S_VLDL_C | Supplement | 128 | -0.14 | (-0.01) | -0.61 | (-0.03) | 0.33 | (0.02) | 0.560 |
| L_LDL_CE | Supplement | 128 | 0.13 | (0.02) | -0.33 | (-0.06) | 0.60 | (0.11) | 0.571 |
| L_LDL_P | Supplement | 128 | 0.13 | (0.00) | -0.34 | (-0.00) | 0.60 | (0.00) | 0.579 |
| M_LDL_L | Supplement | 128 | 0.13 | (0.03) | -0.34 | (-0.07) | 0.60 | (0.12) | 0.582 |
| S_LDL_FC | Supplement | 128 | 0.13 | (0.00) | -0.34 | (-0.01) | 0.60 | (0.01) | 0.582 |
| IDL_C | Supplement | 128 | 0.13 | (0.02) | -0.34 | (-0.06) | 0.60 | (0.11) | 0.585 |
| S_LDL_L | Supplement | 128 | 0.13 | (0.02) | -0.34 | (-0.04) | 0.59 | (0.08) | 0.586 |
| Cit | Dietary advice | 126 | 0.12 | (0.00) | -0.33 | (-0.00) | 0.58 | (0.01) | 0.587 |
| SFA_FA | Supplement | 128 | -0.13 | (-0.24) | -0.60 | (-1.13) | 0.34 | (0.64) | 0.592 |
| S_LDL_TG | Supplement | 128 | -0.12 | (-0.00) | -0.58 | (-0.00) | 0.34 | (0.00) | 0.593 |
| M_LDL_P | Supplement | 128 | 0.13 | (0.00) | -0.34 | (-0.00) | 0.59 | (0.00) | 0.595 |
| S_LDL_P | Supplement | 128 | 0.12 | (0.00) | -0.34 | (-0.00) | 0.59 | (0.00) | 0.602 |
| XL_HDL_C | Supplement | 128 | 0.11 | (0.01) | -0.33 | (-0.02) | 0.55 | (0.04) | 0.618 |
| S_VLDL_L | Dietary advice | 128 | -0.11 | (-0.02) | -0.57 | (-0.09) | 0.34 | (0.05) | 0.621 |
| Tyr | Dietary advice | 128 | -0.11 | (-0.00) | -0.57 | (-0.01) | 0.34 | (0.00) | 0.622 |
| XL_HDL_TG | Supplement | 128 | -0.11 | (-0.00) | -0.57 | (-0.00) | 0.35 | (0.00) | 0.626 |
| S_LDL_PL | Supplement | 128 | 0.12 | (0.00) | -0.36 | (-0.01) | 0.59 | (0.02) | 0.627 |
| Alb | Supplement | 127 | -0.12 | (-0.00) | -0.59 | (-0.00) | 0.36 | (0.00) | 0.630 |
| bOHBut | Supplement | 128 | -0.09 | (-0.00) | -0.46 | (-0.02) | 0.28 | (0.01) | 0.634 |
| IDL_L | Supplement | 128 | 0.11 | (0.03) | -0.36 | (-0.10) | 0.59 | (0.16) | 0.635 |
| FreeC | Supplement | 128 | 0.11 | (0.03) | -0.36 | (-0.10) | 0.58 | (0.16) | 0.638 |
| M_LDL_PL | Supplement | 128 | 0.11 | (0.00) | -0.36 | (-0.01) | 0.59 | (0.02) | 0.642 |
| IDL_CE | Supplement | 128 | 0.11 | (0.01) | -0.36 | (-0.05) | 0.58 | (0.08) | 0.643 |
| cla | Supplement | 127 | -0.10 | (-0.00) | -0.55 | (-0.01) | 0.35 | (0.00) | 0.652 |
| XL_HDL_CE | Supplement | 128 | 0.10 | (0.01) | -0.34 | (-0.02) | 0.54 | (0.03) | 0.654 |
| Ala | Supplement | 128 | 0.09 | (0.01) | -0.34 | (-0.02) | 0.53 | (0.04) | 0.673 |
| TotCho | Supplement | 128 | 0.10 | (0.04) | -0.36 | (-0.14) | 0.55 | (0.22) | 0.676 |
| IDL_P | Supplement | 128 | 0.10 | (0.00) | -0.38 | (-0.00) | 0.58 | (0.00) | 0.678 |
| VLDL_C | Dietary advice | 128 | -0.09 | (-0.02) | -0.53 | (-0.11) | 0.35 | (0.07) | 0.693 |
| Lac | Dietary advice | 128 | 0.10 | (0.06) | -0.39 | (-0.25) | 0.58 | (0.38) | 0.696 |
| S_VLDL_FC | Dietary advice | 128 | -0.09 | (-0.00) | -0.54 | (-0.01) | 0.36 | (0.01) | 0.698 |
| sfa | Supplement | 128 | -0.09 | (-0.08) | -0.55 | (-0.48) | 0.37 | (0.32) | 0.704 |
| TotFA | Supplement | 128 | -0.09 | (-0.19) | -0.55 | (-1.22) | 0.38 | (0.84) | 0.713 |
| S_VLDL_CE | Supplement | 128 | -0.09 | (-0.00) | -0.56 | (-0.02) | 0.39 | (0.01) | 0.717 |
| XS_VLDL_PL | Supplement | 128 | 0.09 | (0.00) | -0.39 | (-0.01) | 0.56 | (0.02) | 0.718 |
| LDL_TG | Dietary advice | 128 | 0.08 | (0.00) | -0.36 | (-0.01) | 0.52 | (0.02) | 0.719 |
| L_LDL_TG | Supplement | 128 | -0.08 | (-0.00) | -0.55 | (-0.01) | 0.38 | (0.01) | 0.728 |
| L_HDL_TG | Supplement | 128 | 0.08 | (0.00) | -0.37 | (-0.00) | 0.52 | (0.00) | 0.731 |
| S_VLDL_PL | Dietary advice | 128 | -0.07 | (-0.00) | -0.53 | (-0.02) | 0.38 | (0.01) | 0.747 |
| S_VLDL_C | Dietary advice | 128 | 0.07 | (0.00) | -0.38 | (-0.02) | 0.51 | (0.03) | 0.762 |
| pufa | Supplement | 128 | 0.07 | (0.05) | -0.39 | (-0.31) | 0.52 | (0.42) | 0.773 |
| Ace | Dietary advice | 128 | 0.05 | (0.00) | -0.33 | (-0.00) | 0.44 | (0.00) | 0.778 |
| S_HDL_PL | Dietary advice | 128 | -0.06 | (-0.00) | -0.49 | (-0.04) | 0.37 | (0.03) | 0.786 |
| TotFA | Dietary advice | 128 | 0.05 | (0.12) | -0.40 | (-0.91) | 0.51 | (1.14) | 0.820 |
| S_HDL_L | Supplement | 128 | 0.05 | (0.01) | -0.41 | (-0.05) | 0.51 | (0.06) | 0.829 |
| Alb | Dietary advice | 127 | 0.04 | (0.00) | -0.34 | (-0.00) | 0.42 | (0.00) | 0.832 |
| S_LDL_TG | Dietary advice | 128 | 0.05 | (0.00) | -0.41 | (-0.00) | 0.51 | (0.00) | 0.834 |
| Remnant_C | Supplement | 128 | -0.05 | (-0.02) | -0.52 | (-0.18) | 0.42 | (0.15) | 0.835 |
| Gln | Supplement | 128 | -0.04 | (-0.00) | -0.46 | (-0.03) | 0.38 | (0.02) | 0.848 |
| dag | Dietary advice | 127 | 0.04 | (0.00) | -0.41 | (-0.00) | 0.49 | (0.01) | 0.853 |
| XS_VLDL_FC | Supplement | 128 | 0.04 | (0.00) | -0.43 | (-0.01) | 0.52 | (0.01) | 0.854 |
| FAw6 | Supplement | 128 | 0.04 | (0.03) | -0.41 | (-0.29) | 0.49 | (0.36) | 0.855 |
| S_HDL_FC | Supplement | 128 | -0.04 | (-0.00) | -0.50 | (-0.01) | 0.42 | (0.01) | 0.863 |
| XS_VLDL_P | Supplement | 128 | -0.04 | (-0.00) | -0.52 | (-0.00) | 0.44 | (0.00) | 0.865 |
| pc | Supplement | 127 | 0.04 | (0.01) | -0.42 | (-0.15) | 0.50 | (0.18) | 0.865 |
| M_LDL_TG | Supplement | 128 | -0.04 | (-0.00) | -0.50 | (-0.01) | 0.42 | (0.00) | 0.869 |
| Glc | Dietary advice | 128 | 0.02 | (0.04) | -0.29 | (-0.45) | 0.34 | (0.52) | 0.881 |
| S_HDL_P | Supplement | 128 | 0.03 | (0.00) | -0.43 | (-0.00) | 0.49 | (0.00) | 0.886 |
| sfa | Dietary advice | 128 | 0.03 | (0.03) | -0.42 | (-0.36) | 0.49 | (0.42) | 0.889 |
| Val | Supplement | 128 | -0.03 | (-0.00) | -0.48 | (-0.02) | 0.43 | (0.02) | 0.904 |
| S_HDL_FC | Dietary advice | 128 | -0.03 | (-0.00) | -0.45 | (-0.01) | 0.40 | (0.01) | 0.906 |
| ApoB | Supplement | 128 | -0.02 | (-0.00) | -0.50 | (-0.09) | 0.45 | (0.08) | 0.920 |
| Gln | Dietary advice | 128 | 0.02 | (0.00) | -0.39 | (-0.02) | 0.43 | (0.03) | 0.922 |
| XS_VLDL_C | Supplement | 128 | 0.02 | (0.00) | -0.45 | (-0.02) | 0.50 | (0.03) | 0.923 |
| S_HDL_PL | Supplement | 128 | -0.02 | (-0.00) | -0.48 | (-0.04) | 0.44 | (0.03) | 0.923 |
| XS_VLDL_L | Supplement | 128 | -0.02 | (-0.00) | -0.50 | (-0.05) | 0.46 | (0.05) | 0.935 |
| LDL_D | Supplement | 128 | 0.02 | (0.00) | -0.49 | (-0.07) | 0.53 | (0.07) | 0.941 |
| dag | Supplement | 127 | 0.01 | (0.00) | -0.41 | (-0.00) | 0.43 | (0.01) | 0.955 |
| Tyr | Supplement | 128 | -0.01 | (-0.00) | -0.42 | (-0.00) | 0.40 | (0.00) | 0.957 |
| XS_VLDL_CE | Supplement | 128 | 0.01 | (0.00) | -0.46 | (-0.02) | 0.49 | (0.02) | 0.957 |
| la | Supplement | 128 | -0.01 | (-0.01) | -0.46 | (-0.29) | 0.44 | (0.28) | 0.961 |
| IDL_TG | Dietary advice | 128 | 0.01 | (0.00) | -0.45 | (-0.01) | 0.47 | (0.01) | 0.963 |
| His | Supplement | 128 | 0.01 | (0.00) | -0.40 | (-0.00) | 0.42 | (0.00) | 0.965 |
| Lac | Supplement | 128 | -0.01 | (-0.00) | -0.35 | (-0.22) | 0.33 | (0.22) | 0.970 |
| L_HDL_TG | Dietary advice | 128 | 0.00 | (0.00) | -0.43 | (-0.00) | 0.44 | (0.00) | 0.982 |

SD=standard deviation; CI=confidence interval.

† Per1-SD difference in metabolite concentrations.

*Non-standardised mean differences expressed in absolute concentration units (e.g. mmol/l metabolite difference between diet or supplement versus placebo).

For abbreviations of metabolic trait names, see Supplementary table 8.

**Supplementary Table 7:** Lycopene and green tea IV regression.

| **Metabolite** | **N** | **beta** | **SE** | **p-value** |
| --- | --- | --- | --- | --- |
| Lycopene | | | | |
| Ace | 116 | 2.13 | 0.78 | 0.006 |
| Pyr | 116 | -1.90 | 0.73 | 0.009 |
| Ala | 116 | -1.55 | 0.65 | 0.016 |
| Val | 116 | -1.79 | 0.78 | 0.023 |
| cla | 114 | -1.73 | 0.78 | 0.025 |
| dag | 108 | -1.81 | 0.81 | 0.026 |
| S_VLDL_C | 116 | -1.41 | 0.69 | 0.041 |
| S_VLDL_CE | 116 | -1.45 | 0.72 | 0.044 |
| VLDL_C | 116 | -1.32 | 0.66 | 0.047 |
| bOHBut | 116 | 1.12 | 0.57 | 0.048 |
| ApoB | 116 | -1.41 | 0.72 | 0.049 |
| Remnant_C | 116 | -1.39 | 0.72 | 0.053 |
| XS_VLDL_P | 116 | -1.40 | 0.72 | 0.054 |
| XS_VLDL_C | 116 | -1.45 | 0.75 | 0.054 |
| XS_VLDL_L | 116 | -1.41 | 0.73 | 0.054 |
| XS_VLDL_CE | 116 | -1.44 | 0.75 | 0.054 |
| XS_VLDL_FC | 116 | -1.41 | 0.75 | 0.059 |
| M_VLDL_CE | 116 | -1.19 | 0.64 | 0.063 |
| S_VLDL_FC | 116 | -1.10 | 0.61 | 0.069 |
| TotFA | 116 | -1.19 | 0.66 | 0.072 |
| S_VLDL_L | 116 | -1.06 | 0.59 | 0.074 |
| Lac | 116 | -1.43 | 0.81 | 0.077 |
| pufa | 116 | -1.17 | 0.67 | 0.083 |
| sfa | 116 | -1.11 | 0.64 | 0.083 |
| TotCho | 116 | -1.19 | 0.68 | 0.083 |
| XS_VLDL_PL | 116 | -1.26 | 0.73 | 0.085 |
| S_VLDL_P | 116 | -0.99 | 0.58 | 0.087 |
| IDL_CE | 116 | -1.27 | 0.74 | 0.087 |
| pc | 115 | -1.12 | 0.66 | 0.088 |
| FAw6 | 116 | -1.18 | 0.70 | 0.091 |
| TotPG | 116 | -1.14 | 0.68 | 0.091 |
| M_VLDL_C | 116 | -1.03 | 0.61 | 0.092 |
| CLA_FA | 114 | -1.19 | 0.71 | 0.095 |
| IDL_C | 116 | -1.23 | 0.74 | 0.097 |
| mufa | 116 | -1.10 | 0.66 | 0.097 |
| dha | 116 | -0.73 | 0.44 | 0.097 |
| IDL_P | 116 | -1.22 | 0.74 | 0.098 |
| M_LDL_PL | 116 | -1.18 | 0.72 | 0.099 |
| IDL_L | 116 | -1.21 | 0.74 | 0.099 |
| la | 116 | -1.15 | 0.70 | 0.100 |
| S_LDL_PL | 116 | -1.15 | 0.71 | 0.104 |
| S_LDL_FC | 116 | -1.16 | 0.72 | 0.107 |
| sm | 116 | -1.15 | 0.71 | 0.107 |
| L_LDL_PL | 116 | -1.16 | 0.73 | 0.109 |
| L_LDL_CE | 116 | -1.17 | 0.73 | 0.109 |
| M_LDL_FC | 116 | -1.16 | 0.72 | 0.110 |
| S_VLDL_PL | 116 | -0.90 | 0.57 | 0.110 |
| L_LDL_P | 116 | -1.16 | 0.73 | 0.111 |
| IDL_PL | 116 | -1.15 | 0.73 | 0.113 |
| L_LDL_L | 116 | -1.15 | 0.73 | 0.113 |
| L_LDL_C | 116 | -1.15 | 0.73 | 0.114 |
| M_LDL_L | 116 | -1.14 | 0.72 | 0.116 |
| M_LDL_P | 116 | -1.13 | 0.72 | 0.117 |
| S_LDL_P | 116 | -1.13 | 0.72 | 0.117 |
| LDL_C | 116 | -1.14 | 0.73 | 0.117 |
| S_LDL_L | 116 | -1.13 | 0.72 | 0.118 |
| M_LDL_C | 116 | -1.13 | 0.72 | 0.119 |
| EstC | 116 | -1.11 | 0.72 | 0.121 |
| M_LDL_CE | 116 | -1.11 | 0.72 | 0.123 |
| Serum_C | 116 | -1.11 | 0.72 | 0.123 |
| IDL_FC | 116 | -1.12 | 0.73 | 0.124 |
| Leu | 116 | -1.03 | 0.67 | 0.125 |
| S_LDL_C | 116 | -1.10 | 0.72 | 0.126 |
| L_LDL_FC | 116 | -1.10 | 0.72 | 0.129 |
| S_LDL_CE | 116 | -1.08 | 0.72 | 0.133 |
| FreeC | 116 | -1.08 | 0.73 | 0.135 |
| IDL_TG | 116 | -1.00 | 0.67 | 0.137 |
| M_VLDL_PL | 116 | -0.83 | 0.57 | 0.150 |
| XXL_VLDL_CE | 116 | -0.89 | 0.63 | 0.153 |
| Glc | 116 | -0.86 | 0.61 | 0.158 |
| XS_VLDL_TG | 116 | -0.82 | 0.58 | 0.162 |
| M_VLDL_L | 116 | -0.78 | 0.57 | 0.171 |
| S_LDL_TG | 116 | -0.87 | 0.65 | 0.181 |
| M_VLDL_P | 116 | -0.76 | 0.57 | 0.183 |
| L_LDL_TG | 116 | -0.91 | 0.69 | 0.184 |
| XXL_VLDL_C | 116 | -0.81 | 0.62 | 0.190 |
| M_VLDL_FC | 116 | -0.75 | 0.58 | 0.195 |
| Gp | 116 | -0.71 | 0.56 | 0.205 |
| XXL_VLDL_P | 116 | -0.75 | 0.60 | 0.212 |
| XXL_VLDL_L | 116 | -0.75 | 0.60 | 0.213 |
| XXL_VLDL_PL | 116 | -0.75 | 0.61 | 0.214 |
| HDL3_C | 116 | -0.83 | 0.67 | 0.215 |
| XXL_VLDL_TG | 116 | -0.73 | 0.60 | 0.222 |
| Glol | 116 | 0.82 | 0.70 | 0.243 |
| FAw3 | 116 | -0.54 | 0.47 | 0.246 |
| ApoA1 | 116 | -0.75 | 0.65 | 0.247 |
| LDL_TG | 116 | -0.76 | 0.68 | 0.258 |
| Tyr | 116 | -0.75 | 0.67 | 0.262 |
| L_VLDL_CE | 116 | -0.64 | 0.57 | 0.267 |
| M_VLDL_TG | 116 | -0.61 | 0.55 | 0.269 |
| Ile | 116 | -0.76 | 0.70 | 0.275 |
| Alb | 115 | -0.64 | 0.59 | 0.276 |
| S_VLDL_TG | 116 | -0.57 | 0.52 | 0.280 |
| XL_VLDL_FC | 116 | -0.65 | 0.61 | 0.286 |
| M_LDL_TG | 116 | -0.70 | 0.67 | 0.292 |
| XL_VLDL_C | 116 | -0.63 | 0.60 | 0.293 |
| XL_VLDL_PL | 116 | -0.63 | 0.60 | 0.295 |
| XXL_VLDL_FC | 116 | -0.64 | 0.61 | 0.296 |
| XL_VLDL_CE | 116 | -0.62 | 0.60 | 0.301 |
| XL_HDL_TG | 116 | -0.71 | 0.69 | 0.301 |
| L_VLDL_C | 116 | -0.60 | 0.58 | 0.306 |
| Serum_TG | 116 | -0.58 | 0.57 | 0.314 |
| L_VLDL_PL | 116 | -0.55 | 0.57 | 0.339 |
| S_HDL_CE | 116 | -0.65 | 0.68 | 0.341 |
| L_VLDL_FC | 116 | -0.55 | 0.59 | 0.354 |
| S_HDL_C | 116 | -0.59 | 0.66 | 0.370 |
| L_HDL_TG | 116 | -0.54 | 0.61 | 0.372 |
| L_VLDL_L | 116 | -0.49 | 0.57 | 0.388 |
| Gln | 116 | -0.50 | 0.59 | 0.394 |
| XL_VLDL_L | 116 | -0.50 | 0.59 | 0.395 |
| His | 116 | -0.55 | 0.64 | 0.396 |
| L_VLDL_P | 116 | -0.48 | 0.57 | 0.399 |
| VLDL_TG | 116 | -0.46 | 0.55 | 0.409 |
| XL_VLDL_P | 116 | -0.48 | 0.59 | 0.412 |
| HDL_TG | 116 | -0.52 | 0.66 | 0.430 |
| M_HDL_TG | 116 | -0.46 | 0.59 | 0.434 |
| L_VLDL_TG | 116 | -0.43 | 0.57 | 0.446 |
| Gly | 116 | -0.40 | 0.53 | 0.455 |
| M_HDL_CE | 116 | -0.45 | 0.61 | 0.459 |
| HDL_C | 116 | -0.41 | 0.56 | 0.465 |
| XL_VLDL_TG | 116 | -0.42 | 0.59 | 0.475 |
| M_HDL_C | 116 | -0.43 | 0.61 | 0.482 |
| Phe | 116 | -0.46 | 0.66 | 0.488 |
| S_HDL_L | 116 | -0.41 | 0.64 | 0.517 |
| S_HDL_P | 116 | -0.40 | 0.63 | 0.527 |
| HDL2_C | 116 | -0.33 | 0.54 | 0.538 |
| L_HDL_PL | 116 | -0.29 | 0.50 | 0.559 |
| M_HDL_L | 116 | -0.35 | 0.61 | 0.562 |
| M_HDL_P | 116 | -0.35 | 0.61 | 0.565 |
| M_HDL_FC | 116 | -0.34 | 0.61 | 0.580 |
| VLDL_D | 116 | 0.29 | 0.53 | 0.587 |
| S_HDL_TG | 116 | 0.29 | 0.54 | 0.587 |
| LA_FA | 116 | -0.29 | 0.54 | 0.598 |
| SFA_FA | 116 | 0.33 | 0.64 | 0.608 |
| L_HDL_P | 116 | -0.24 | 0.48 | 0.620 |
| L_HDL_L | 116 | -0.22 | 0.47 | 0.644 |
| FALen | 116 | 0.28 | 0.61 | 0.650 |
| Cit | 108 | -0.24 | 0.56 | 0.668 |
| UnSat | 116 | 0.23 | 0.54 | 0.670 |
| DHA_FA | 116 | -0.19 | 0.46 | 0.679 |
| M_HDL_PL | 116 | -0.23 | 0.61 | 0.699 |
| XL_HDL_FC | 116 | 0.18 | 0.50 | 0.712 |
| Crea | 116 | -0.19 | 0.54 | 0.722 |
| FAw3_FA | 116 | 0.18 | 0.50 | 0.724 |
| S_HDL_PL | 116 | -0.18 | 0.62 | 0.769 |
| L_HDL_CE | 116 | -0.13 | 0.45 | 0.770 |
| S_HDL_FC | 116 | -0.18 | 0.63 | 0.770 |
| L_HDL_C | 116 | -0.12 | 0.45 | 0.795 |
| MUFA_FA | 116 | -0.13 | 0.58 | 0.817 |
| FAw6_FA | 116 | -0.13 | 0.60 | 0.824 |
| PUFA_FA | 116 | -0.11 | 0.60 | 0.856 |
| L_HDL_FC | 116 | -0.06 | 0.45 | 0.886 |
| XL_HDL_C | 116 | 0.06 | 0.53 | 0.914 |
| HDL_D | 116 | -0.03 | 0.47 | 0.957 |
| LDL_D | 116 | -0.02 | 0.61 | 0.977 |
| XL_HDL_P | 116 | -0.01 | 0.49 | 0.978 |
| XL_HDL_PL | 116 | -0.01 | 0.45 | 0.990 |
| XL_HDL_CE | 116 | 0.01 | 0.54 | 0.992 |
| XL_HDL_L | 116 | 0.00 | 0.49 | 0.997 |
| Green tea | | | | |
| Gly | 116 | -2.83 | 3.13 | 0.366 |
| S_HDL_CE | 116 | 4.39 | 5.48 | 0.423 |
| M_LDL_PL | 116 | 3.54 | 4.47 | 0.428 |
| M_LDL_FC | 116 | 3.59 | 4.54 | 0.430 |
| S_LDL_L | 116 | 3.56 | 4.51 | 0.430 |
| S_LDL_P | 116 | 3.59 | 4.55 | 0.430 |
| L_LDL_PL | 116 | 3.06 | 3.88 | 0.431 |
| M_LDL_P | 116 | 3.31 | 4.20 | 0.431 |
| M_LDL_L | 116 | 3.29 | 4.18 | 0.432 |
| S_LDL_C | 116 | 3.21 | 4.09 | 0.432 |
| S_LDL_PL | 116 | 4.06 | 5.17 | 0.432 |
| S_LDL_CE | 116 | 3.01 | 3.85 | 0.434 |
| M_LDL_C | 116 | 3.07 | 3.94 | 0.436 |
| S_LDL_FC | 116 | 3.95 | 5.07 | 0.436 |
| LDL_C | 116 | 2.99 | 3.84 | 0.436 |
| L_LDL_L | 116 | 2.99 | 3.84 | 0.436 |
| L_LDL_P | 116 | 3.03 | 3.90 | 0.437 |
| L_LDL_FC | 116 | 2.73 | 3.52 | 0.437 |
| FALen | 116 | -3.34 | 4.31 | 0.439 |
| M_LDL_CE | 116 | 2.94 | 3.79 | 0.439 |
| Serum_C | 116 | 3.29 | 4.25 | 0.439 |
| EstC | 116 | 3.33 | 4.32 | 0.440 |
| L_LDL_C | 116 | 2.83 | 3.67 | 0.440 |
| L_LDL_CE | 116 | 2.85 | 3.71 | 0.442 |
| IDL_PL | 116 | 2.79 | 3.64 | 0.443 |
| pufa | 116 | 4.17 | 5.44 | 0.443 |
| S_HDL_C | 116 | 4.93 | 6.46 | 0.445 |
| FreeC | 116 | 3.11 | 4.07 | 0.445 |
| FAw6 | 116 | 4.41 | 5.79 | 0.446 |
| IDL_FC | 116 | 2.59 | 3.41 | 0.447 |
| XS_VLDL_PL | 116 | 3.03 | 4.00 | 0.450 |
| IDL_L | 116 | 2.76 | 3.66 | 0.451 |
| IDL_P | 116 | 2.80 | 3.72 | 0.452 |
| IDL_C | 116 | 2.63 | 3.50 | 0.453 |
| la | 116 | 4.06 | 5.44 | 0.456 |
| IDL_CE | 116 | 2.61 | 3.51 | 0.458 |
| sm | 116 | 5.23 | 7.15 | 0.464 |
| XS_VLDL_FC | 116 | 3.27 | 4.52 | 0.470 |
| HDL3_C | 116 | 5.12 | 7.21 | 0.478 |
| His | 116 | 6.35 | 9.00 | 0.481 |
| ApoA1 | 116 | 3.71 | 5.36 | 0.489 |
| XS_VLDL_L | 116 | 2.79 | 4.04 | 0.490 |
| S_HDL_FC | 116 | 3.22 | 4.67 | 0.491 |
| M_LDL_TG | 116 | 4.07 | 5.93 | 0.493 |
| XS_VLDL_C | 116 | 2.32 | 3.42 | 0.497 |
| M_HDL_FC | 116 | 3.98 | 5.86 | 0.497 |
| XS_VLDL_P | 116 | 2.78 | 4.09 | 0.497 |
| S_HDL_L | 116 | 4.61 | 6.83 | 0.499 |
| S_HDL_P | 116 | 4.50 | 6.68 | 0.501 |
| M_HDL_PL | 116 | 3.81 | 5.67 | 0.501 |
| HDL_C | 116 | 3.11 | 4.63 | 0.502 |
| M_HDL_L | 116 | 3.84 | 5.74 | 0.503 |
| M_HDL_C | 116 | 3.82 | 5.73 | 0.504 |
| M_HDL_P | 116 | 3.83 | 5.73 | 0.504 |
| TotPG | 116 | 5.44 | 8.16 | 0.505 |
| M_HDL_CE | 116 | 3.77 | 5.68 | 0.506 |
| ApoB | 116 | 2.65 | 4.02 | 0.509 |
| TotCho | 116 | 5.10 | 7.72 | 0.509 |
| HDL2_C | 116 | 2.76 | 4.20 | 0.510 |
| L_LDL_TG | 116 | 3.52 | 5.35 | 0.511 |
| UnSat | 116 | -2.78 | 4.25 | 0.514 |
| S_HDL_PL | 116 | 2.65 | 4.06 | 0.514 |
| dha | 116 | 1.89 | 2.91 | 0.516 |
| LDL_TG | 116 | 3.29 | 5.10 | 0.518 |
| Glc | 116 | 2.05 | 3.21 | 0.523 |
| Remnant_C | 116 | 2.28 | 3.59 | 0.524 |
| XS_VLDL_CE | 116 | 1.86 | 2.95 | 0.527 |
| bOHBut | 116 | 3.74 | 5.97 | 0.531 |
| S_LDL_TG | 116 | 3.57 | 5.69 | 0.531 |
| dag | 108 | 4.60 | 7.37 | 0.533 |
| pc | 115 | 3.80 | 6.10 | 0.533 |
| TotFA | 116 | 4.51 | 7.27 | 0.536 |
| L_HDL_PL | 116 | 2.31 | 3.74 | 0.536 |
| Ile | 116 | -1.29 | 2.10 | 0.538 |
| Crea | 116 | -2.42 | 4.02 | 0.546 |
| sfa | 116 | 4.85 | 8.05 | 0.547 |
| S_VLDL_CE | 116 | 2.27 | 3.79 | 0.550 |
| IDL_TG | 116 | 2.89 | 4.85 | 0.551 |
| L_HDL_P | 116 | 1.95 | 3.32 | 0.557 |
| Gln | 116 | 1.91 | 3.26 | 0.558 |
| L_HDL_L | 116 | 1.92 | 3.28 | 0.558 |
| S_VLDL_C | 116 | 2.33 | 3.99 | 0.559 |
| Leu | 116 | -1.33 | 2.29 | 0.560 |
| LDL_D | 116 | -2.32 | 4.00 | 0.562 |
| S_VLDL_PL | 116 | 2.46 | 4.27 | 0.565 |
| FAw3 | 116 | 1.54 | 2.69 | 0.568 |
| Ace | 116 | 1.85 | 3.25 | 0.569 |
| MUFA_FA | 116 | -1.98 | 3.47 | 0.569 |
| L_HDL_FC | 116 | 1.66 | 2.93 | 0.572 |
| Glol | 116 | 1.98 | 3.53 | 0.575 |
| S_VLDL_FC | 116 | 2.28 | 4.12 | 0.579 |
| L_HDL_C | 116 | 1.59 | 2.87 | 0.580 |
| L_HDL_CE | 116 | 1.57 | 2.86 | 0.583 |
| XS_VLDL_TG | 116 | 2.20 | 4.07 | 0.589 |
| CLA_FA | 114 | -4.20 | 7.93 | 0.596 |
| S_VLDL_L | 116 | 1.79 | 3.51 | 0.609 |
| S_VLDL_P | 116 | 1.69 | 3.39 | 0.617 |
| mufa | 116 | 2.56 | 5.34 | 0.631 |
| S_VLDL_TG | 116 | 1.38 | 2.89 | 0.633 |
| Val | 116 | -1.16 | 2.44 | 0.634 |
| FAw6_FA | 116 | 1.05 | 2.28 | 0.643 |
| Cit | 108 | 0.76 | 1.70 | 0.656 |
| VLDL_C | 116 | 1.27 | 2.91 | 0.662 |
| Alb | 115 | 1.07 | 2.45 | 0.663 |
| XL_HDL_CE | 116 | 0.88 | 2.02 | 0.663 |
| Lac | 116 | 1.81 | 4.39 | 0.681 |
| M_VLDL_TG | 116 | 1.00 | 2.50 | 0.689 |
| XL_HDL_C | 116 | 0.74 | 1.89 | 0.697 |
| M_VLDL_FC | 116 | 1.01 | 2.61 | 0.698 |
| M_VLDL_PL | 116 | 1.00 | 2.62 | 0.702 |
| HDL_D | 116 | 0.71 | 1.85 | 0.702 |
| M_HDL_TG | 116 | 1.09 | 2.89 | 0.707 |
| XXL_VLDL_CE | 116 | -0.79 | 2.16 | 0.714 |
| M_VLDL_P | 116 | 0.89 | 2.47 | 0.720 |
| Ala | 116 | -1.08 | 3.03 | 0.722 |
| M_VLDL_L | 116 | 0.87 | 2.47 | 0.725 |
| L_HDL_TG | 116 | 0.94 | 2.73 | 0.731 |
| Tyr | 116 | 0.99 | 2.94 | 0.736 |
| VLDL_D | 116 | -0.59 | 1.80 | 0.745 |
| XL_HDL_P | 116 | 0.53 | 1.65 | 0.750 |
| XL_HDL_L | 116 | 0.52 | 1.65 | 0.752 |
| FAw3_FA | 116 | -0.59 | 1.89 | 0.753 |
| LA_FA | 116 | 0.60 | 2.03 | 0.768 |
| XXL_VLDL_FC | 116 | 0.70 | 2.41 | 0.771 |
| XXL_VLDL_PL | 116 | 0.70 | 2.44 | 0.773 |
| PUFA_FA | 116 | 0.54 | 1.90 | 0.775 |
| M_VLDL_C | 116 | 0.69 | 2.42 | 0.777 |
| Phe | 116 | -1.13 | 4.07 | 0.782 |
| Gp | 116 | 1.41 | 5.15 | 0.784 |
| M_VLDL_CE | 116 | 0.60 | 2.36 | 0.800 |
| Serum_TG | 116 | 0.52 | 2.19 | 0.812 |
| XL_HDL_FC | 116 | 0.39 | 1.66 | 0.816 |
| XXL_VLDL_TG | 116 | 0.52 | 2.23 | 0.817 |
| L_VLDL_FC | 116 | 0.50 | 2.20 | 0.820 |
| XL_HDL_PL | 116 | 0.32 | 1.45 | 0.826 |
| cla | 114 | -0.77 | 3.62 | 0.831 |
| HDL_TG | 116 | 0.49 | 2.52 | 0.847 |
| XXL_VLDL_P | 116 | 0.42 | 2.19 | 0.848 |
| XXL_VLDL_L | 116 | 0.40 | 2.19 | 0.856 |
| XL_VLDL_PL | 116 | 0.40 | 2.22 | 0.858 |
| XL_VLDL_TG | 116 | 0.37 | 2.06 | 0.859 |
| Pyr | 116 | -0.54 | 3.26 | 0.868 |
| SFA_FA | 116 | 0.39 | 2.32 | 0.868 |
| XL_VLDL_P | 116 | 0.33 | 2.07 | 0.874 |
| XL_VLDL_FC | 116 | 0.34 | 2.19 | 0.877 |
| XL_VLDL_L | 116 | 0.32 | 2.08 | 0.878 |
| L_VLDL_PL | 116 | 0.30 | 2.04 | 0.881 |
| L_VLDL_C | 116 | 0.28 | 2.02 | 0.890 |
| VLDL_TG | 116 | 0.27 | 1.95 | 0.890 |
| L_VLDL_L | 116 | 0.25 | 1.97 | 0.897 |
| L_VLDL_P | 116 | 0.24 | 1.96 | 0.903 |
| L_VLDL_TG | 116 | 0.23 | 1.94 | 0.906 |
| XXL_VLDL_C | 116 | -0.24 | 2.05 | 0.908 |
| XL_HDL_TG | 116 | 0.21 | 2.30 | 0.929 |
| XL_VLDL_C | 116 | 0.11 | 2.04 | 0.957 |
| XL_VLDL_CE | 116 | -0.09 | 1.95 | 0.964 |
| L_VLDL_CE | 116 | 0.08 | 1.90 | 0.968 |
| S_HDL_TG | 116 | -0.05 | 1.88 | 0.977 |
| DHA_FA | 116 | 0.02 | 1.65 | 0.989 |

Results show the SD increase in metabolite traits for each unit increase in green tea. Results are adjusted for baseline metabolic traits concentration.

Lycopene: F=2.21; R²=0.100

Green tea: F=0.31; R²=0.00

For abbreviations of metabolic trait names, see Supplementary table 8.

**Supplementary Table 8:** Metabolic trait abbreviations.

| **Metabolite, lipid, or lipoprotein lipid** | **Abbreviation** | **Unit** | **Class** |
| --- | --- | --- | --- |
| Alanine | Ala | mmol/l | Amino Acids |
| Glutamine | Gln | mmol/l | Amino Acids |
| Glycine | Gly | mmol/l | Amino Acids |
| Histidine | His | mmol/l | Amino Acids |
| Isoleucine | Ile | mmol/l | Amino Acids |
| Leucine | Leu | mmol/l | Amino Acids |
| Phenylalanine | Phe | mmol/l | Amino Acids |
| Tyrosine | Tyr | mmol/l | Amino Acids |
| Valine | Val | mmol/l | Amino Acids |
| Docosahexaenoic acid | DHA | mmol/l | Fatty Acids & Saturation |
| Ratio of docosahexaenoic acid to total fatty acids | DHA/FA | % | Fatty Acids & Saturation |
| Omega-3 fatty acids | FAw3 | mmol/l | Fatty Acids & Saturation |
| Ratio of omega-3 fatty acids to total fatty acids | FAw3/FA | % | Fatty Acids & Saturation |
| Omega-6 fatty acids | FAw6 | mmol/l | Fatty Acids & Saturation |
| Ratio of omega-6 fatty acids to total fatty acids | FAw6/FA | % | Fatty Acids & Saturation |
| Linoleic acid | LA | mmol/l | Fatty Acids & Saturation |
| Ratio of linoleic acid to total fatty acids | LA/FA | % | Fatty Acids & Saturation |
| Monounsaturated fatty acids; 16:1, 18:1 | MUFA | mmol/l | Fatty Acids & Saturation |
| Ratio of monounsaturated fatty acids to total fatty acids | MUFA/FA | % | Fatty Acids & Saturation |
| Polyunsaturated fatty acids | PUFA | mmol/l | Fatty Acids & Saturation |
| Ratio of polyunsaturated fatty acids to total fatty acids | PUFA/FA | % | Fatty Acids & Saturation |
| Saturated fatty acids | SAFA | mmol/l | Fatty Acids & Saturation |
| Ratio of saturated fatty acids to total fatty acids | SAFA/FA | % | Fatty Acids & Saturation |
| Total fatty acids | TotFA | mmol/l | Fatty Acids & Saturation |
| Fatty acids degree of unsaturation | UnSat |  | Fatty Acids & Saturation |
| Albumin | Alb | signal area | Fluid Balance |
| Creatinine | Crea | mmol/l | Fluid Balance |
| Citrate | Cit | mmol/l | Glycolysis Related Metabolites |
| Glucose | Glc | mmol/l | Glycolysis Related Metabolites |
| Glycerol | Glol | mmol/l | Glycolysis Related Metabolites |
| Lactate | Lac | mmol/l | Glycolysis Related Metabolites |
| Pyruvate | Pyr | mmol/l | Glycolysis Related Metabolites |
| Acetoacetate | AcAce | mmol/l | Ketone Bodies |
| Acetate | Ace | mmol/l | Ketone Bodies |
| 3-hydroxybutyrate | bOHBut | mmol/l | Ketone Bodies |
| Glycoprotein acetyls, mainly a1-acid glycoprotein | Gp | mmol/l | Inflammation |
| Triglycerides in HDL | HDL-TG | mmol/l | Glycerides & Phospholipids |
| Triglycerides in LDL | LDL-TG | mmol/l | Glycerides & Phospholipids |
| Phosphatidylcholine and other cholines | PC | mmol/l | Glycerides & Phospholipids |
| Serum total triglycerides | Serum-TG | mmol/l | Glycerides & Phospholipids |
| Sphingomyelins | SM | mmol/l | Glycerides & Phospholipids |
| Ratio of triglycerides to phosphoglycerides | TG/PG |  | Glycerides & Phospholipids |
| Total cholines | TotCho | mmol/l | Glycerides & Phospholipids |
| Total phosphoglycerides | TotPG | mmol/l | Glycerides & Phospholipids |
| Triglycerides in VLDL | VLDL-TG | mmol/l | Glycerides & Phospholipids |
| Apolipoprotein A-I | ApoA1 | g/l | Apolipoproteins |
| Apolipoprotein B | ApoB | g/l | Apolipoproteins |
| Ratio of apolipoprotein B to apolipoprotein A-I | ApoB/ApoA1 |  | Apolipoproteins |
| Esterified cholesterol | EstC | mmol/l | Cholesterol |
| Free cholesterol | FreeC | mmol/l | Cholesterol |
| Total cholesterol in HDL2 | HDL2-C | mmol/l | Cholesterol |
| Total cholesterol in HDL3 | HDL3-C | mmol/l | Cholesterol |
| Total cholesterol in HDL | HDL-C | mmol/l | Cholesterol |
| Total cholesterol in LDL | LDL-C | mmol/l | Cholesterol |
| Remnant cholesterol (non-HDL, non-LDL -cholesterol) | Remnant-C | mmol/l | Cholesterol |
| Serum total cholesterol | Serum-C | mmol/l | Cholesterol |
| Total cholesterol in VLDL | VLDL-C | mmol/l | Cholesterol |
| Mean diameter for HDL particles | HDL-D | nm | Lipoprotein Particle Sizes |
| Mean diameter for LDL particles | LDL-D | nm | Lipoprotein Particle Sizes |
| Mean diameter for VLDL particles | VLDL-D | nm | Lipoprotein Particle Sizes |
| Total cholesterol in IDL | IDL-C | mmol/l | Lipoprotein Subclasses |
| Cholesterol esters in IDL | IDL-CE | mmol/l | Lipoprotein Subclasses |
| Free cholesterol in IDL | IDL-FC | mmol/l | Lipoprotein Subclasses |
| Free cholesterol to total lipids ratio in IDL | IDL-FC-% | % | Lipoprotein Subclasses |
| Total lipids in IDL | IDL-L | mmol/l | Lipoprotein Subclasses |
| Concentration of IDL particles | IDL-P | umol/l | Lipoprotein Subclasses |
| Phospholipids in IDL | IDL-PL | mmol/l | Lipoprotein Subclasses |
| Triglycerides in IDL | IDL-TG | mmol/l | Lipoprotein Subclasses |
| Total cholesterol in large HDL | L-HDL-C | mmol/l | Lipoprotein Subclasses |
| Cholesterol esters in large HDL | L-HDL-CE | mmol/l | Lipoprotein Subclasses |
| Free cholesterol in large HDL | L-HDL-FC | mmol/l | Lipoprotein Subclasses |
| Total lipids in large HDL | L-HDL-L | mmol/l | Lipoprotein Subclasses |
| Concentration of large HDL particles | L-HDL-P | umol/l | Lipoprotein Subclasses |
| Phospholipids in large HDL | L-HDL-PL | mmol/l | Lipoprotein Subclasses |
| Triglycerides in large HDL | L-HDL-TG | mmol/l | Lipoprotein Subclasses |
| Total cholesterol in medium HDL | M-HDL-C | mmol/l | Lipoprotein Subclasses |
| Cholesterol esters in medium HDL | M-HDL-CE | mmol/l | Lipoprotein Subclasses |
| Free cholesterol in medium HDL | M-HDL-FC | mmol/l | Lipoprotein Subclasses |
| Total lipids in medium HDL | M-HDL-L | mmol/l | Lipoprotein Subclasses |
| Concentration of medium HDL particles | M-HDL-P | umol/l | Lipoprotein Subclasses |
| Phospholipids in medium HDL | M-HDL-PL | mmol/l | Lipoprotein Subclasses |
| Triglycerides in medium HDL | M-HDL-TG | mmol/l | Lipoprotein Subclasses |
| Total cholesterol in small HDL | S-HDL-C | mmol/l | Lipoprotein Subclasses |
| Cholesterol esters in small HDL | S-HDL-CE | mmol/l | Lipoprotein Subclasses |
| Free cholesterol in small HDL | S-HDL-FC | mmol/l | Lipoprotein Subclasses |
| Total lipids in small HDL | S-HDL-L | mmol/l | Lipoprotein Subclasses |
| Concentration of small HDL particles | S-HDL-P | umol/l | Lipoprotein Subclasses |
| Phospholipids in small HDL | S-HDL-PL | mmol/l | Lipoprotein Subclasses |
| Triglycerides in small HDL | S-HDL-TG | mmol/l | Lipoprotein Subclasses |
| Total cholesterol in very large HDL | XL-HDL-C | mmol/l | Lipoprotein Subclasses |
| Cholesterol esters in very large HDL | XL-HDL-CE | mmol/l | Lipoprotein Subclasses |
| Free cholesterol in very large HDL | XL-HDL-FC | mmol/l | Lipoprotein Subclasses |
| Total lipids in very large HDL | XL-HDL-L | mmol/l | Lipoprotein Subclasses |
| Concentration of very large HDL particles | XL-HDL-P | umol/l | Lipoprotein Subclasses |
| Phospholipids in very large HDL | XL-HDL-PL | mmol/l | Lipoprotein Subclasses |
| Triglycerides in very large HDL | XL-HDL-TG | mmol/l | Lipoprotein Subclasses |
| Total cholesterol in large LDL | L-LDL-C | mmol/l | Lipoprotein Subclasses |
| Cholesterol esters in large LDL | L-LDL-CE | mmol/l | Lipoprotein Subclasses |
| Free cholesterol in large LDL | L-LDL-FC | mmol/l | Lipoprotein Subclasses |
| Total lipids in large LDL | L-LDL-L | mmol/l | Lipoprotein Subclasses |
| Concentration of large LDL particles | L-LDL-P | umol/l | Lipoprotein Subclasses |
| Phospholipids in large LDL | L-LDL-PL | mmol/l | Lipoprotein Subclasses |
| Triglycerides in large LDL | L-LDL-TG | mmol/l | Lipoprotein Subclasses |
| Total cholesterol in medium LDL | M-LDL-C | mmol/l | Lipoprotein Subclasses |
| Cholesterol esters in medium LDL | M-LDL-CE | mmol/l | Lipoprotein Subclasses |
| Free cholesterol in medium LDL | M-LDL-FC | mmol/l | Lipoprotein Subclasses |
| Total lipids in medium LDL | M-LDL-L | mmol/l | Lipoprotein Subclasses |
| Concentration of medium LDL particles | M-LDL-P | umol/l | Lipoprotein Subclasses |
| Phospholipids in medium LDL | M-LDL-PL | mmol/l | Lipoprotein Subclasses |
| Triglycerides in medium LDL | M-LDL-TG | mmol/l | Lipoprotein Subclasses |
| Total cholesterol in small LDL | S-LDL-C | mmol/l | Lipoprotein Subclasses |
| Cholesterol esters in small LDL | S-LDL-CE | mmol/l | Lipoprotein Subclasses |
| Free cholesterol in small LDL | S-LDL-FC | mmol/l | Lipoprotein Subclasses |
| Total lipids in small LDL | S-LDL-L | mmol/l | Lipoprotein Subclasses |
| Concentration of small LDL particles | S-LDL-P | umol/l | Lipoprotein Subclasses |
| Phospholipids in small LDL | S-LDL-PL | mmol/l | Lipoprotein Subclasses |
| Triglycerides in small LDL | S-LDL-TG | mmol/l | Lipoprotein Subclasses |
| Total cholesterol in large VLDL | L-VLDL-C | mmol/l | Lipoprotein Subclasses |
| Cholesterol esters in large VLDL | L-VLDL-CE | mmol/l | Lipoprotein Subclasses |
| Free cholesterol in large VLDL | L-VLDL-FC | mmol/l | Lipoprotein Subclasses |
| Total lipids in large VLDL | L-VLDL-L | mmol/l | Lipoprotein Subclasses |
| Concentration of large VLDL particles | L-VLDL-P | umol/l | Lipoprotein Subclasses |
| Phospholipids in large VLDL | L-VLDL-PL | mmol/l | Lipoprotein Subclasses |
| Triglycerides in large VLDL | L-VLDL-TG | mmol/l | Lipoprotein Subclasses |
| Total cholesterol in medium VLDL | M-VLDL-C | mmol/l | Lipoprotein Subclasses |
| Cholesterol esters in medium VLDL | M-VLDL-CE | mmol/l | Lipoprotein Subclasses |
| Free cholesterol in medium VLDL | M-VLDL-FC | mmol/l | Lipoprotein Subclasses |
| Total lipids in medium VLDL | M-VLDL-L | mmol/l | Lipoprotein Subclasses |
| Concentration of medium VLDL particles | M-VLDL-P | umol/l | Lipoprotein Subclasses |
| Phospholipids in medium VLDL | M-VLDL-PL | mmol/l | Lipoprotein Subclasses |
| Triglycerides in medium VLDL | M-VLDL-TG | mmol/l | Lipoprotein Subclasses |
| Total cholesterol in small VLDL | S-VLDL-C | mmol/l | Lipoprotein Subclasses |
| Cholesterol esters in small VLDL | S-VLDL-CE | mmol/l | Lipoprotein Subclasses |
| Free cholesterol in small VLDL | S-VLDL-FC | mmol/l | Lipoprotein Subclasses |
| Total lipids in small VLDL | S-VLDL-L | mmol/l | Lipoprotein Subclasses |
| Concentration of small VLDL particles | S-VLDL-P | umol/l | Lipoprotein Subclasses |
| Phospholipids in small VLDL | S-VLDL-PL | mmol/l | Lipoprotein Subclasses |
| Triglycerides in small VLDL | S-VLDL-TG | mmol/l | Lipoprotein Subclasses |
| Total cholesterol in very large VLDL | XL-VLDL-C | mmol/l | Lipoprotein Subclasses |
| Cholesterol esters in very large VLDL | XL-VLDL-CE | mmol/l | Lipoprotein Subclasses |
| Free cholesterol in very large VLDL | XL-VLDL-FC | mmol/l | Lipoprotein Subclasses |
| Total lipids in very large VLDL | XL-VLDL-L | mmol/l | Lipoprotein Subclasses |
| Concentration of very large VLDL particles | XL-VLDL-P | umol/l | Lipoprotein Subclasses |
| Phospholipids in very large VLDL | XL-VLDL-PL | mmol/l | Lipoprotein Subclasses |
| Triglycerides in very large VLDL | XL-VLDL-TG | mmol/l | Lipoprotein Subclasses |
| Total cholesterol in very small VLDL | XS-VLDL-C | mmol/l | Lipoprotein Subclasses |
| Cholesterol esters in very small VLDL | XS-VLDL-CE | mmol/l | Lipoprotein Subclasses |
| Free cholesterol in very small VLDL | XS-VLDL-FC | mmol/l | Lipoprotein Subclasses |
| Total lipids in very small VLDL | XS-VLDL-L | mmol/l | Lipoprotein Subclasses |
| Concentration of very small VLDL particles | XS-VLDL-P | umol/l | Lipoprotein Subclasses |
| Phospholipids in very small VLDL | XS-VLDL-PL | mmol/l | Lipoprotein Subclasses |
| Triglycerides in very small VLDL | XS-VLDL-TG | mmol/l | Lipoprotein Subclasses |
| Total cholesterol in chylomicrons and extremely large VLDL | XXL-VLDL-C | mmol/l | Lipoprotein Subclasses |
| Cholesterol esters in chylomicrons and extremely large VLDL | XXL-VLDL-CE | mmol/l | Lipoprotein Subclasses |
| Free cholesterol in chylomicrons and extremely large VLDL | XXL-VLDL-FC | mmol/l | Lipoprotein Subclasses |
| Total lipids in chylomicrons and extremely large VLDL | XXL-VLDL-L | mmol/l | Lipoprotein Subclasses |
| Concentration of chylomicrons and extremely large VLDL particles | XXL-VLDL-P | umol/l | Lipoprotein Subclasses |
| Phospholipids in chylomicrons and extremely large VLDL | XXL-VLDL-PL | mmol/l | Lipoprotein Subclasses |
| Triglycerides in chylomicrons and extremely large VLDL | XXL-VLDL-TG | mmol/l | Lipoprotein Subclasses |

**Supplementary Table 9:** Representative coefficients of variation for selected metabolic traits in Kettunen et al (2016).

| **Measure** | **NMR signal location (ppm)** | CV% |
| --- | --- | --- |
| XXL-VLDL-P | 0.912 | 16.2 % |
| XXL-VLDL-L | 0.912 | 16.3 % |
| XXL-VLDL-PL | 0.912 | 14.8 % |
| XXL-VLDL-TG | 0.912 | 15.8 % |
| XL-VLDL-P | 0.897 | 11.9 % |
| XL-VLDL-L | 0.897 | 12.5 % |
| XL-VLDL-PL | 0.897 | 14.5 % |
| XL-VLDL-TG | 0.897 | 10.2 % |
| L-VLDL-P | 0.895 | 1.8 % |
| L-VLDL-L | 0.895 | 2.1 % |
| L-VLDL-PL | 0.895 | 2.1 % |
| L-VLDL-C | 0.895 | 6.2 % |
| L-VLDL-CE | 0.895 | 7.9 % |
| L-VLDL-FC | 0.895 | 4.7 % |
| L-VLDL-TG | 0.895 | 1.6 % |
| M-VLDL-P | 0.893 | 1.0 % |
| M-VLDL-L | 0.893 | 1.2 % |
| M-VLDL-PL | 0.893 | 1.4 % |
| M-VLDL-C | 0.893 | 3.9 % |
| M-VLDL-CE | 0.893 | 6.0 % |
| M-VLDL-FC | 0.893 | 1.2 % |
| M-VLDL-TG | 0.893 | 1.4 % |
| S-VLDL-P | 0.89 | 1.3 % |
| S-VLDL-L | 0.89 | 1.4 % |
| S-VLDL-PL | 0.89 | 2.2 % |
| S-VLDL-C | 0.89 | 4.0 % |
| S-VLDL-FC | 0.89 | 1.9 % |
| S-VLDL-TG | 0.89 | 2.7 % |
| XS-VLDL-P | 0.884 | 4.3 % |
| XS-VLDL-L | 0.884 | 4.7 % |
| XS-VLDL-PL | 0.884 | 2.6 % |
| XS-VLDL-TG | 0.884 | 2.2 % |
| IDL-P | 0.882 | 3.4 % |
| IDL-L | 0.882 | 3.6 % |
| IDL-PL | 0.882 | 3.4 % |
| IDL-C | 0.882 | 4.4 % |
| IDL-FC | 0.882 | 3.2 % |
| IDL-TG | 0.882 | 2.1 % |
| L-LDL-P | 0.881 | 2.4 % |
| L-LDL-L | 0.881 | 2.5 % |
| L-LDL-PL | 0.881 | 2.5 % |
| L-LDL-C | 0.881 | 2.9 % |
| L-LDL-CE | 0.881 | 2.9 % |
| L-LDL-FC | 0.881 | 2.8 % |
| M-LDL-P | 0.871 | 1.8 % |
| M-LDL-L | 0.871 | 1.9 % |
| M-LDL-PL | 0.871 | 1.9 % |
| M-LDL-C | 0.871 | 2.2 % |
| M-LDL-CE | 0.871 | 2.4 % |
| S-LDL-P | 0.869 | 1.8 % |
| S-LDL-L | 0.869 | 1.8 % |
| S-LDL-C | 0.869 | 2.2 % |
| XL-HDL-P | 0.851 | 10.2 % |
| XL-HDL-L | 0.851 | 10.6 % |
| XL-HDL-PL | 0.851 | 7.7 % |
| XL-HDL-C | 0.851 | 15.0 % |
| XL-HDL-CE | 0.851 | 15.8 % |
| XL-HDL-FC | 0.851 | 13.9 % |
| XL-HDL-TG | 0.851 | 12.5 % |
| L-HDL-P | 0.848 | 3.5 % |
| L-HDL-L | 0.848 | 3.6 % |
| L-HDL-PL | 0.848 | 3.5 % |
| L-HDL-C | 0.848 | 4.0 % |
| L-HDL-CE | 0.848 | 3.8 % |
| L-HDL-FC | 0.848 | 5.2 % |
| M-HDL-P | 0.838 | 3.6 % |
| M-HDL-L | 0.838 | 3.7 % |
| M-HDL-PL | 0.838 | 3.1 % |
| M-HDL-C | 0.838 | 4.7 % |
| M-HDL-CE | 0.838 | 4.7 % |
| M-HDL-FC | 0.838 | 4.8 % |
| S-HDL-P | 0.834 | 3.1 % |
| S-HDL-L | 0.834 | 3.2 % |
| S-HDL-TG | 0.834 | 3.1 % |
| VLDL-D | Derivative | 0.3 % |
| LDL-D | Derivative | 0.3 % |
| HDL-D | Derivative | 0.4 % |
| Serum-C | 0.87 | 2.1 % |
| Est-C | 4.61 | 2.4 % |
| Free-C | 3.53 | 2.6 % |
| LDL-C | 0.87 | 2.3 % |
| HDL-C | 0.84 | 2.3 % |
| Serum-TG | 0.89 | 1.2 % |
| TotPG | 4.42 | 3.4 % |
| PC | 4.38 | 4.1 % |
| SM | 5.67 | 4.6 % |
| ApoA1 | 0.85 | 1.6 % |
| ApoB | 0.88 | 2.2 % |
| TotFA | 2.31 | 3.8 % |
| FALen | Derivative | 1.1 % |
| Bis-DB-ratio | Derivative | 2.5 % |
| Bis-FA-ratio | Derivative | 1.9 % |
| CH2-in-FA | Derivative | 1.4 % |
| CH2-DB-ratio | Derivative | 1.2 % |
| DB-in-FA | Derivative | 2.2 % |
| DHA | 2.38 | 2.7 % |
| LA | 2.77 | 3.3 % |
| FAw3 | 0.98 | 2.7 % |
| FAw6 | 0.89 | 4.5 % |
| FAw79S | 0.88 | 2.3 % |
| MUFA | 2.01 | 3.0 % |
| otPUFA | 2.83 | 6.4 % |
| Glc | 5.25 | 2.6 % |
| Lac | 1.35 | 3.2 % |
| Pyr | 2.38 | 4.7 % |
| Cit | 2.68 | 10.1 % |
| Glol | 3.67 | 6.3 % |
| Ala | 1.5 | 2.3 % |
| Gln | 2.47 | 2.7 % |
| Gly | 3.58 | 7.7 % |
| His | 7.07 | 8.9 % |
| Ile | 1.03 | 3.4 % |
| Leu | 0.97 | 1.9 % |
| Val | 1.06 | 2.3 % |
| Phe | 7.36 | 3.9 % |
| Tyr | 6.92 | 7.7 % |
| Ace | 1.93 | 5.5 % |
| AcAce | 2.24 | 3.6 % |
| bOHBut | 1.22 | 3.2 % |
| Crea | 4.07 | 3.9 % |
| Alb | 6.8 | 1.5 % |
| Urea | 5.75 | 19.5 % |
| Gp | 2.06 | 1.1 % |

Abbreviations: CV, coefficient of variation.

"NMR signal location" refers to the most representative chemical shift values for the metabolic measures in the NMR spectra (many metabolites have a complicated signal with components at multiple chemical shift locations). The representative coefficients of variation (in percent) are calculated for each metabolic measure based on 100 quality control serum samples in relation to the population mean of each metabolic measure. The control samples are processed identically with the actual study samples; one sample being placed in every 96-sample well plate. For further details, please see Kettunen J, Tukiainen T, Sarin AP, et al. Genome-wide association study identifies multiple loci influencing human serum metabolite levels. Nat Genet 2012;44(3):269-76.

**Supplementary Figure 1:** Linear fit between intervention arms vs placebo models, adjusted for baseline metabolite levels.


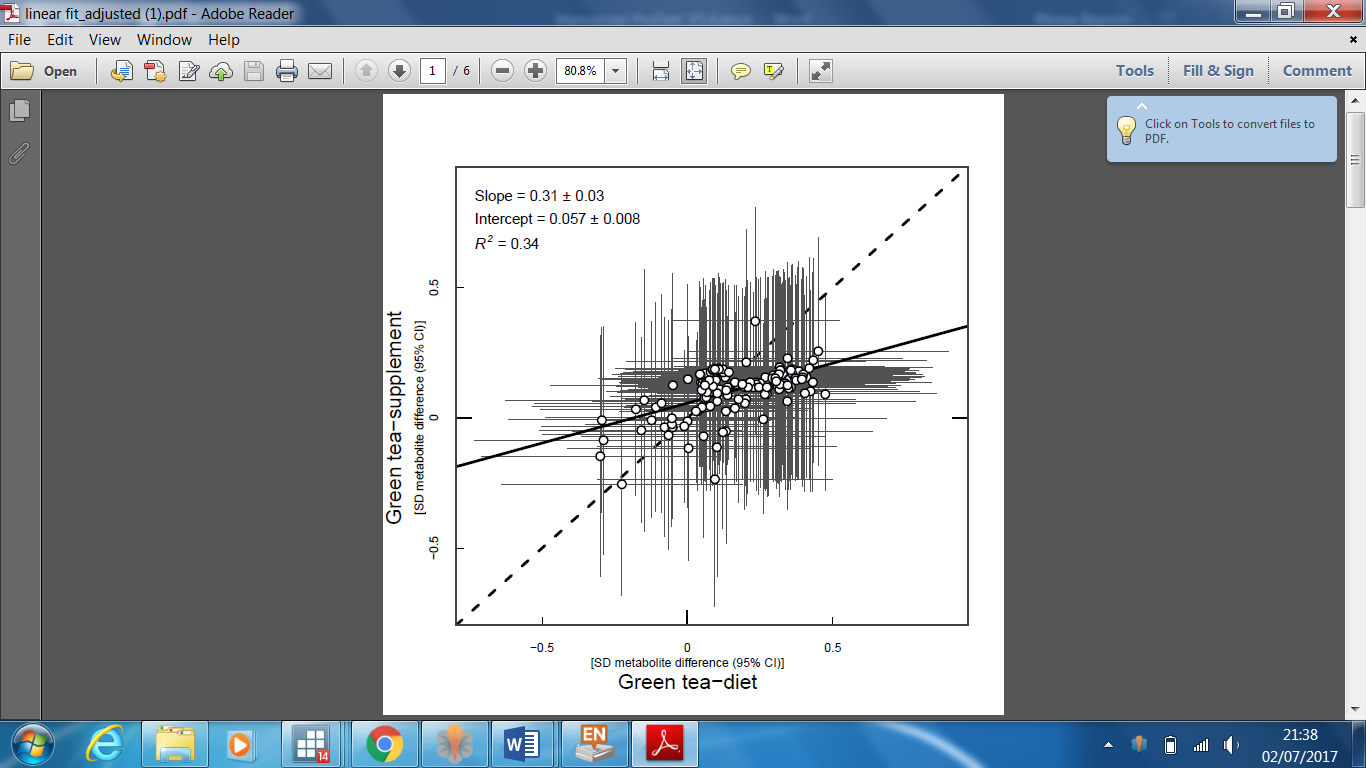

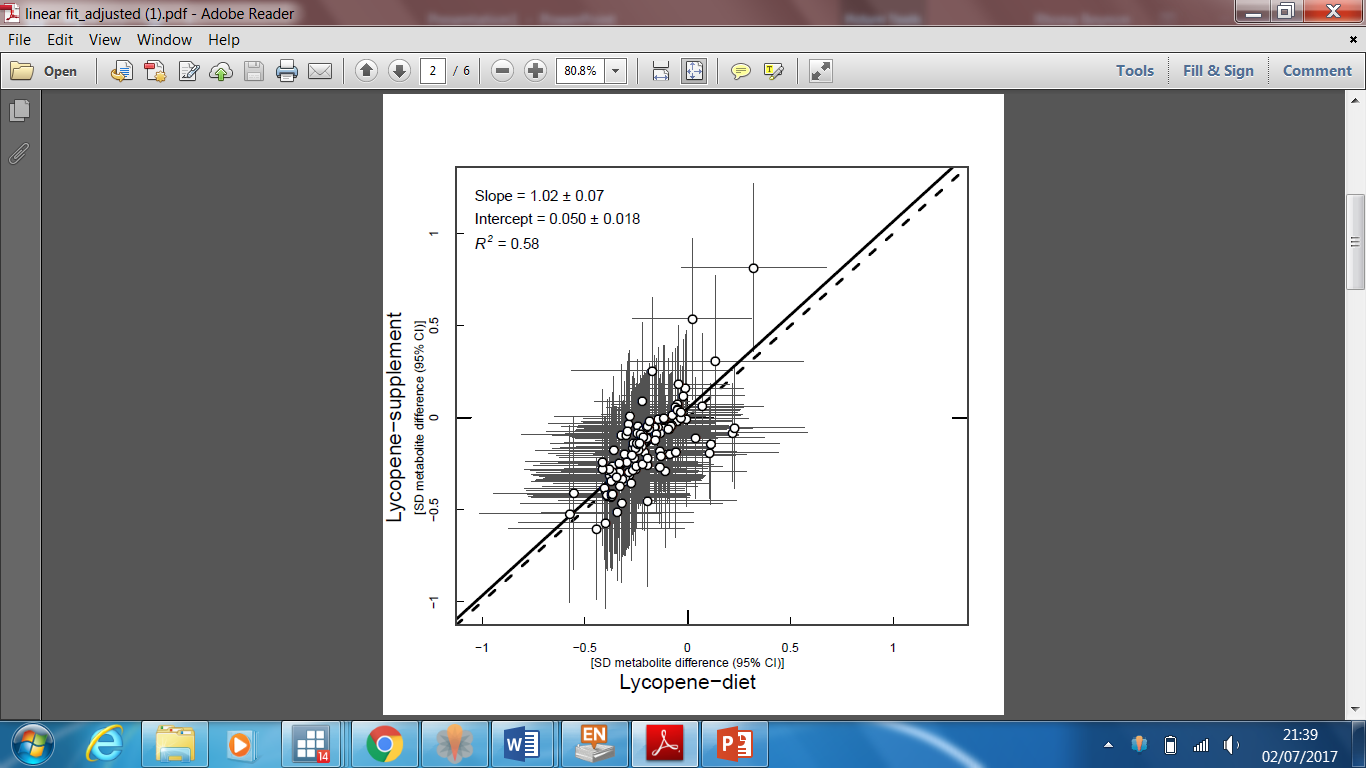


(Left) estimates of the standard deviation (SD) difference in metabolic trait concentration between lycopene dietary advice and placebo arms at follow-up (*x*-axis), adjusted for baseline metabolite levels, plotted against the SD difference in metabolic trait concentration in the lycopene supplement arm vs placebo (*y*-axis), adjusted for baseline metabolites. (Right) corresponding results for green tea. Each dot on the plot represents an individual metabolic trait. The horizontal grey lines on each dot denote the confidence intervals (CI) for diet associations and the vertical grey lines indicate the CI for supplement estimates. A linear fit of the overall correspondence summarizes the similarity in magnitude between diet and supplement associations (black solid lines). A slope of 1 with an intercept of 0 (dashed black line), with all dots sitting on that line (R^2^=1), would indicate that diet and supplement estimates had the same magnitude and direction.


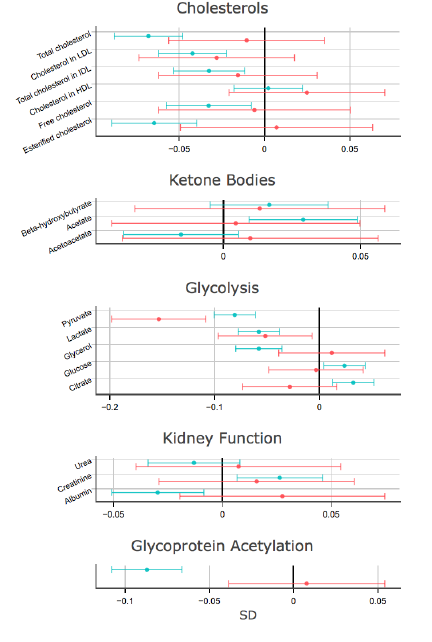

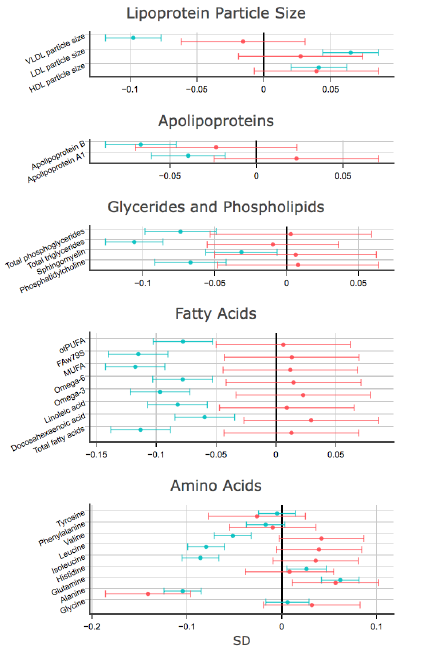
**
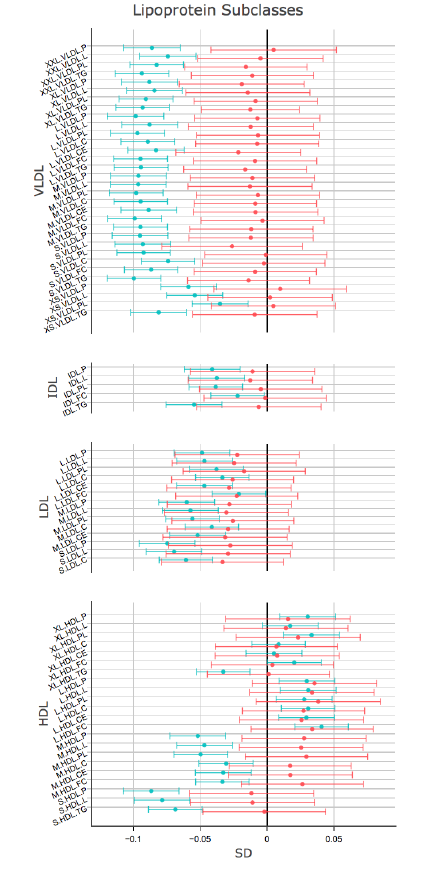
Supplementary Figure 2**: Forest plots showing the associations of rs74249229 and rs1260326 with metabolic trait measures.

Key: rs74249229 (chr 16: PDRP)

rs1260326 (chr 2: GCKR)

For each of the two single-nucleotide polymorphisms (SNPs) considered for use in the pyruvate Mendelian randomization, forest plots show the standard deviation (SD) difference in metabolites per effect allele of SNP. Abbreviations: GCKR, glucokinase regulator; PDRP, pyruvate dehydrogenase phosphatase regulatory subunit. For metabolic trait names, see Supplementary Table 13. Figures provided by Sebastian Soidinsalo.

**Supplementary Methods**

**The ProDiet randomised controlled trial**

The ProDiet RCT (ISRCTN 95931417) was nested within the Prostate Cancer testing and Treatment study (ProtecT). It was a randomised feasibility trial, which aimed to establish the feasibility and acceptance of specific dietary interventions in men with elevated prostate specific antigen (PSA) levels (29).

Between December 2009 and May 2010, 113 men between the ages of 50 and 69 years, with PSA results between 2.0 and 2.95ng/ml or at least 3.0ng/ml with a negative biopsy, were recruited into the trial from nine primary care practices in the South West of England. The selected PSA thresholds were based on previous findings that men with modestly raised PSA levels had an increased risk of prostate cancer (32).

Trial participants were randomised (in a 2 x 3 factorial design) to daily lycopene (active capsule, lycopene-rich diet or placebo capsule) and green tea (active capsule, tea drink or placebo capsule) for six months, as described previously (1). The men were blinded to which capsule they received (active or placebo). Blinding of participants to the dietary intervention was not possible in this study for practical reasons.

Men were found to adhere successfully to both lycopene and green tea dietary interventions, as evidenced by important increases in circulating levels of lycopene and EGCG (a bioactive component of green tea) in the diet and supplement groups compared to the placebo groups.

**Dietary assessment**

Men reported frequency of intake for each of the food item (n=114) across nine categories, ranging from “never/less than once per month” to “six or more times per day”. Information on portion sizes and nutrient intake were derived for each food item using McCance and Widdowson’s Composition of Foods (2), as described previously (3). Food intake was computed as the product of frequency of intake and nutrient content per portion of food. Green tea consumption was assessed in terms of frequency of intake (daily versus not). Lycopene-rich food items included tomatoes, tomato ketchup, tomato juice, pizza and baked beans. The lycopene content of foods was obtained from the European carotenoid database compiled by O’Neill and colleagues (4).

**Measurements of serum lycopene and plasma EGCG**

Serum lycopene levels were measured using reversed-phase high-performance liquid chromatography (HPLC) (5). Plasma EGCG levels were analysed and quantified using HPLC-mass spectroscopy (MS), as described by Stalmach et al (6).

**Measurement of metabolic traits**

Metabolic profiling was performed using a high-throughput serum nuclear magnetic resonance (NMR) metabolomics platform. Full details have been described elsewhere (7-9). Briefly, 260 μL of plasma and 260 μL of sodium phosphate buffer (75 mM Na2HPO4, 0.08% sodium 3-(trimethylsilyl) propionate-2,2,3,3-d4, 0.04% sodium azide in 80%/20% H20/D2O, pH 7.4) were mixed and transferred to NMR tubes using an eight-channel, Varispan Janus liquid handling robot (PerkinElmer). NMR spectra were acquired using a Bruker Avance III HD 600MHz spectrometer equipped with a nitrogen-cooled triple resonance probe (CryoProbe Prodigy TCI). Both spectrometers were equipped with SampleJet auto-samplers with cooled (6°C) sample storage. The methodology combines three “molecular windows”, two of which (LIPO and low-molecular-weight molecule (LMWM) are applied to the native serum and one to the serum lipid extracts (LIPID). Lipid spectra were obtained after a standardised lipid extraction procedure performed on each sample using a VIAFLO 96 channel electronic pipette (Integra Biosciences). Data pre-processing and quantification were as previously described (7-9). The combination of these molecular windows provides simultaneous quantification in absolute concentration units of 14 lipoprotein subclasses, their lipid concentrations and composition, apolipoprotein A-I and B, multiple cholesterol and triglyceride measures, albumin, various fatty acids as well as on numerous low-molecular-weight metabolites, including amino acids, glycolysis related measures and ketone bodies.

This platform has been applied in various large-scale epidemiological and genetic studies (10-12). A detailed protocol, including information on quality control, has been published elsewhere (8, 9). The 14 lipoprotein subclass sizes were defined as follows: very low density lipoprotein (VLDL) is subdivided into six subclasses, the largest being extremely large VLDL with particle diameters from 75 nm upwards and a possible contribution of chylomicrons, and five remaining VLDL subclasses (average particle diameters of 64.0 nm, 53.6 nm, 44.5 nm, 36.8 nm, and 31.3 nm); Intermediate density lipoprotein (IDL) (28.6 nm), three low density lipoprotein (LDL) subclasses (25.5 nm, 23.0 nm, and 18.7 nm), and four high density lipoprotein (HDL) subclasses (14.3 nm, 12.1 nm, 10.9 nm, and 8.7 nm). The mean sizes for VLDL, LDL and HDL particles were calculated by weighting the corresponding subclass diameters with their particle concentrations (12).

There exists high analytical consistency between metabolic measures quantified by the NMR metabolomics platform and concentrations obtained from routine clinical chemistry (13) or other analytical methods, e.g. gas chromatography (10, 13) and enzymatic methods (13), with correlations >0.9. Previous work has also demonstrated consistency of biomarker associations with disease incidence for metabolic traits quantified by NMR and two widely used mass spectroscopy platforms (10, 13).

**IV analyses**

IV analysis was implemented using 2-stage least squares (2SLS) regression. F-statistics and R2 values from the first-stage regression between intervention arm and serum lycopene/EGCG levels were examined to check the instrumental variable assumption that the instrument is sufficiently associated with the exposure. Causal estimates for the instrumented effect of serum lycopene/EGCG levels on each follow-up metabolite were obtained from the second-stage regression. Regressions were adjusted for the same metabolite at baseline.

**Mendelian randomization**

Mendelian randomization is an application of the technique of instrumental variable (IV) analysis, whereby genetic variants are used as instrumental variables (IVs) (i.e. proxies for the exposure of interest) to examine the causal effect of a modifiable exposure on an outcome of interest (14, 15). Mendelian randomization is based on a number of assumptions: firstly, that the IV is associated with an exposure of interest; secondly, that the IV is independent of confounding between the exposure and the outcome, and thirdly that the IV is not associated with the outcome of interest – except through its association with the exposure of interest (15). Utilization of this approach depends on the existence of genetic variants that are robustly associated with metabolic trait levels.

We first identified genetic variants that are robustly associated with our metabolic traits of interest, using data from a recently published genome-wide association study (GWAS) of 123 circulating metabolic traits (16) levels. Independent single nucleotide polymorphism (SNP) effects that met genome-wide significance (p < 5x10-8) were used as genetic instruments. To leverage power for the Mendelian randomization analysis, we obtained summary data from a GWAS for N= 44,825 cases and N= 27,904 controls of European ancestry in the PRACTICAL (Prostate Cancer Association Group to Investigate Cancer Associated Alterations in the Genome) consortium (17), to establish the gene-outcome associations. We performed a look-up of all SNPs associated with the metabolic traits of interest in the prostate cancer GWAS summary data. We extracted the following summary data for each SNP: the log odds ratio (OR) per copy of the effect allele, its standard error, the effect allele and the non-effect allele.

We combined information from the genome-outcome associations from PRACTICAL with information on the gene-exposure associations from the GWAS of metabolite levels in a two-sample Mendelian randomization approach (18). We calculated the log OR for prostate cancer per standard deviation (SD) unit increase in metabolic trait levels using the Wald ratio method (SEs calculated using delta method), and validated findings using two further tests: likelihood based methods and MR Egger (19, 20).

**References**

1. Horwood JP, Avery KN, Metcalfe C, Donovan JL, Hamdy FC, Neal DE, et al. Men's knowledge and attitudes towards dietary prevention of a prostate cancer diagnosis: a qualitative study. BMC Cancer. 2014;14:812.

2. Lane JA, Donovan JL, Davis M, Walsh E, Dedman D, Down L, et al. Active monitoring, radical prostatectomy, or radiotherapy for localised prostate cancer: study design and diagnostic and baseline results of the ProtecT randomised phase 3 trial. Lancet Oncol. 2014;15(10):1109-18.

3. Er V, Lane JA, Martin RM, Emmett P, Gilbert R, Avery KN, et al. Adherence to dietary and lifestyle recommendations and prostate cancer risk in the prostate testing for cancer and treatment (ProtecT) trial. Cancer Epidemiol Biomarkers Prev. 2014;23(10):2066-77.

4. O'Neill ME, Carroll Y, Corridan B, Olmedilla B, Granado F, Blanco I, et al. A European carotenoid database to assess carotenoid intakes and its use in a five-country comparative study. Br J Nutr. 2001;85(4):499-507.

5. Craft N. Carotenoid reversedd-phase high-performance liquid chromatography methods: reference compendium. Methods Enzymology. 1992;213:185-205.

6. Stalmach A, Troufflard S, Serafini M, Crozier A. Absorption, metabolism and excretion of Choladi green tea flavan-3-ols by humans. Mol Nutr Food Res. 2009;53 Suppl 1:S44-53.

7. Soininen P, Kangas AJ, Wurtz P, Suna T, Ala-Korpela M. Quantitative serum nuclear magnetic resonance metabolomics in cardiovascular epidemiology and genetics. Circ Cardiovasc Genet. 2015;8(1):192-206.

8. Soininen P, Kangas AJ, Wurtz P, Tukiainen T, Tynkkynen T, Laatikainen R, et al. High-throughput serum NMR metabonomics for cost-effective holistic studies on systemic metabolism. Analyst. 2009;134(9):1781-5.

9. Inouye M, Kettunen J, Soininen P, Silander K, Ripatti S, Kumpula LS, et al. Metabonomic, transcriptomic, and genomic variation of a population cohort. Mol Syst Biol. 2010;6:441.

10. Wurtz P, Havulinna AS, Soininen P, Tynkkynen T, Prieto-Merino D, Tillin T, et al. Metabolite profiling and cardiovascular event risk: a prospective study of 3 population-based cohorts. Circulation. 2015;131(9):774-85.

11. Kettunen J, Tukiainen T, Sarin AP, Ortega-Alonso A, Tikkanen E, Lyytikainen LP, et al. Genome-wide association study identifies multiple loci influencing human serum metabolite levels. Nature genetics. 2012;44(3):269-76.

12. Kujala UM, Makinen VP, Heinonen I, Soininen P, Kangas AJ, Leskinen TH, et al. Long-term leisure-time physical activity and serum metabolome. Circulation. 2013;127(3):340-8.

13. Würtz P, Kangas, A. J., Soininen, P., Lawlor, D. A., Davey Smith, G., & Ala-Korpela, M. . Quantitative Serum NMR Metabolomics in Large-Scale Epidemiology: A Primer on-Omic Technology. American Journal of Epidemiology. 2017;kwx016.

14. Lawlor DA, Harbord RM, Sterne JA, Timpson N, Davey Smith G. Mendelian randomization: using genes as instruments for making causal inferences in epidemiology. Stat Med. 2008;27(8):1133-63.

15. Didelez V, Sheehan N. Mendelian randomization as an instrumental variable approach to causal inference. Stat Methods Med Res. 2007;16(4):309-30.

16. Kettunen J, Demirkan A, Wurtz P, Draisma HH, Haller T, Rawal R, et al. Genome-wide study for circulating metabolites identifies 62 loci and reveals novel systemic effects of LPA. Nat Commun. 2016;7:11122.

17. Kote-Jarai Z, Easton DF, Stanford JL, Ostrander EA, Schleutker J, Ingles SA, et al. Multiple novel prostate cancer predisposition loci confirmed by an international study: the PRACTICAL Consortium. Cancer Epidemiol Biomarkers Prev. 2008;17(8):2052-61.

18. Pierce BL, Burgess S. Efficient design for Mendelian randomization studies: subsample and 2-sample instrumental variable estimators. Am J Epidemiol. 2013;178(7):1177-84.

19. Bowden J, Davey Smith G, Burgess S. Mendelian randomization with invalid instruments: effect estimation and bias detection through Egger regression. Int J Epidemiol. 2015;44(2):512-25.

20. Burgess S, Dudbridge F, Thompson SG. Combining information on multiple instrumental variables in Mendelian randomization: comparison of allele score and summarized data methods. Stat Med. 2016;35(11):1880-906.

**The PRACTICAL Consortium** (http://practical.icr.ac.uk/):

**OncoArray European and Asian samples**

Rosalind A. Eeles1,2, Brian E. Henderson3*, Christopher A. Haiman3, ZSofia Kote-Jarai1, Fredrick R. Schumacher4,5, Ali Amin Al Olama6,7, Sara Benlloch6,1, Kenneth Muir8,9, Sonja I. Berndt10, David V. Conti3, Fredrik Wiklund11, Stephen Chanock10, Susan Gapster12, Victoria L. Stevens12, Catherine M. Tangen13, Jyotsna Batra14,15, Judith Clements14,15, Australian Prostate Cancer Research Centre BioResource (APCB)14, Henrik Gronberg11, Nora Pashayan16,17, Johanna Schleutker18,19, Demetrius Albanes10, Alicja Wolk20, Catharine West21, Lorelei Mucci22, Géraldine Cancel-Tassin23,24, Stella Koutros10, Karina Dalsgaard Sorensen25,26, Lovise Maehle27, David E. Neal28,29,30, Freddie C. Hamdy30, Jenny L. Donovan31, Ruth C. Travis32, Robert J. Hamilton33, Sue Ann Ingles3, Barry S. Rosenstein34,35, Yong-Jie Lu36, Graham G. Giles37,38, Adam S. Kibel39, Ana Vega40, Manolis Kogevinas41,42,43,44, Kathryn L. Penney45, Jong Y. Park46, Janet L. Stanford47,48, Cezary Cybulski49, Børge G. Nordestgaard50,51, Hermann Brenner52,53,54, Christiane Maier55, Jeri Kim56, Esther M. John57,58, Manuel R. Teixeira59,60, Susan L. Neuhausen61, Kim De Ruyck62, Azad Razack63, Lisa F. Newcomb47,64, Davor Lessel65, Radka Kaneva66, Nawaid Usmani67,68, Frank Claessens69, Paul A. Townsend70, Manuela Gago Dominguez71,72, Monique J. Roobol73, Florence Menegaux74, Kay-Tee Khaw75, Lisa Cannon-Albright76,77, Hardev Pandha78, Stephen N. Thibodeau79

*In memorium

1 The Institute of Cancer Research, London, UK.

2 Royal Marsden NHS Foundation Trust, London, UK.

3 Department of Preventive Medicine, Keck School of Medicine, University of Southern California/Norris Comprehensive Cancer Center, Los Angeles, CA, USA.

4 Department of Epidemiology and Biostatistics, Case Western Reserve University, Cleveland, OH, USA.

5 Seidman Cancer Center, University Hospitals, Cleveland, OH, USA.

6 Centre for Cancer Genetic Epidemiology, Department of Public Health and Primary Care, University of Cambridge, Strangeways Research Laboratory, Cambridge, UK.

7 University of Cambridge, Department of Clinical Neurosciences, Cambridge, UK.

8 Division of Population Health, Health Services Research and Primary Care, University of Manchester, Manchester, UK.

9 Warwick Medical School, University of Warwick, Coventry, UK.

10 Division of Cancer Epidemiology and Genetics, National Cancer Institute, NIH, Bethesda, MD, USA.

11 Department of Medical Epidemiology and Biostatistics, Karolinska Institute, Stockholm, Sweden.

12 Epidemiology Research Program, American Cancer Society, 250 Williams Street, Atlanta, GA, USA.

13 SWOG Statistical Center, Fred Hutchinson Cancer Research Center, Seattle, WA, USA.

14 Australian Prostate Cancer Research Centre-Qld, Institute of Health and Biomedical Innovation and School of Biomedical Science, Queensland University of Technology, Brisbane, Queensland, Australia.

15 Translational Research Institute, Brisbane, Queensland, Australia.

16 University College London, Department of Applied Health Research, London, UK.

17 Centre for Cancer Genetic Epidemiology, Department of Oncology, University of Cambridge, Strangeways Laboratory, Cambridge, UK.

18 Department of Medical Biochemistry and Genetics, Institute of Biomedicine, University of Turku, Finland.

19 Tyks Microbiology and Genetics, Department of Medical Genetics, Turku University Hospital, Finland.

20 Division of Nutritional Epidemiology, Institute of Environmental Medicine, Karolinska Institutet, Sweden.

21 Division of Cancer Sciences, University of Manchester, Manchester Academic Health Science Centre, Radiotherapy Related Research, Manchester NIHR Biomedical Research Centre, The Christie Hospital NHS Foundation Trust, Manchester, UK.

22 Department of Epidemiology, Harvard T.H Chan School of Public Health, Boston, MA, USA.

23 CeRePP, Tenon Hospital, Paris, France.

24 UPMC Sorbonne Universites, GRC N°5 ONCOTYPE-URO, Tenon Hospital, Paris, France.

25 Department of Molecular Medicine, Aarhus University Hospital, Denmark.

26 Department of Clinical Medicine, Aarhus University, Denmark.

27 Department of Medical Genetics, Oslo University Hospital, Norway.

28 University of Cambridge, Department of Oncology, Addenbrooke's Hospital, Cambridge, UK.

29 Cancer Research UK Cambridge Research Institute, Li Ka Shing Centre, Cambridge, UK.

30 Nuffield Department of Surgical Sciences, University of Oxford, Oxford, UK, Faculty of Medical Science, University of Oxford, John Radcliffe Hospital, Oxford, UK.

31 School of Social and Community Medicine, University of Bristol, Bristol, UK.

32 Cancer Epidemiology Unit, Nuffield Department of Population Health University of Oxford, Oxford, UK.

33 Dept. of Surgical Oncology, Princess Margaret Cancer Centre, Toronto, Canada.

34 Department of Radiation Oncology, Icahn School of Medicine at Mount Sinai, New York, NY, USA.

35 Department of Genetics and Genomic Sciences, Icahn School of Medicine at Mount Sinai, New York, NY, USA.

36 Centre for Molecular Oncology, Barts Cancer Institute, Queen Mary University of London, John Vane Science Centre, London, UK.

37 Cancer Epidemiology & Intelligence Division, The Cancer Council Victoria, Melbourne, Victoria, Australia.

38 Centre for Epidemiology and Biostatistics, Melbourne School of Population and Global Health, The University of Melbourne, Melbourne, Australia.

39 Division of Urologic Surgery, Brigham and Womens Hospital, Boston, MA, USA.

40 Fundación Pública Galega de Medicina Xenómica-SERGAS, Grupo de Medicina Xenómica, CIBERER, IDIS, Santiago de Compostela, Spain.

41 Centre for Research in Environmental Epidemiology (CREAL), Barcelona Institute for Global Health (ISGlobal), Barcelona, Spain.

42 CIBER Epidemiología y Salud Pública (CIBERESP), Madrid, Spain.

43 IMIM (Hospital del Mar Research Institute), Barcelona, Spain.

44 Universitat Pompeu Fabra (UPF), Barcelona, Spain.

45 Channing Division of Network Medicine, Department of Medicine, Brigham and Women's Hospital/Harvard Medical School, Boston, MA, USA.

46 Department of Cancer Epidemiology, Moffitt Cancer Center, Tampa, USA.

47 Division of Public Health Sciences, Fred Hutchinson Cancer Research Center, Seattle, Washington, USA.

48 Department of Epidemiology, School of Public Health, University of Washington, Seattle, Washington, USA.

49 International Hereditary Cancer Center, Department of Genetics and Pathology, Pomeranian Medical University, Szczecin, Poland.

50 Faculty of Health and Medical Sciences, University of Copenhagen, Denmark.

51 Department of Clinical Biochemistry, Herlev and Gentofte Hospital, Copenhagen University Hospital, Herlev, Denmark.

52 Division of Clinical Epidemiology and Aging Research, German Cancer Research Center (DKFZ), Heidelberg, Germany.

53 German Cancer Consortium (DKTK), German Cancer Research Center (DKFZ), Heidelberg, Germany.

54 Division of Preventive Oncology, German Cancer Research Center (DKFZ) and National Center for Tumor Diseases (NCT), Heidelberg, Germany.

55 Institute for Human Genetics, University Hospital Ulm, Ulm, Germany.

56 The University of Texas MD Anderson Cancer Center, Department of Genitourinary Medical Oncology, Houston, TX, USA.

57 Cancer Prevention Institute of California, Fremont, CA, USA.

58 Department of Health Research & Policy (Epidemiology) and Stanford Cancer Institute, Stanford University School of Medicine, Stanford, CA , USA.

59 Department of Genetics, Portuguese Oncology Institute of Porto, Porto, Portugal.

60 Biomedical Sciences Institute (ICBAS), University of Porto, Porto, Portugal.

61 Department of Population Sciences, Beckman Research Institute of the City of Hope, Duarte, CA, USA.

62 Ghent University, Faculty of Medicine and Health Sciences, Basic Medical Sciences, Gent, Belgium.

63 Department of Surgery, Faculty of Medicine, University of Malaya, Kuala Lumpur, Malaysia.

64 Department of Urology, University of Washington, Seattle, WA, USA.

65 Institute of Human Genetics, University Medical Center Hamburg-Eppendorf, Hamburg, Germany.

66 Molecular Medicine Center, Department of Medical Chemistry and Biochemistry, Medical University, Sofia, Bulgaria.

67 Department of Oncology, Cross Cancer Institute, University of Alberta, Edmonton, Alberta, Canada.

68 Division of Radiation Oncology, Cross Cancer Institute, Edmonton, Alberta, Canada.

69 Molecular Endocrinology Laboratory, Department of Cellular and Molecular Medicine, KU Leuven, Leuven, Belgium.

70 Institute of Cancer Sciences, Manchester Cancer Research Centre, University of Manchester, Manchester Academic Health Science Centre, St Mary's Hospital, Manchester, UK.

71 Genomic Medicine Group, Galician Foundation of Genomic Medicine, Instituto de Investigacion Sanitaria de Santiago de Compostela (IDIS), Complejo Hospitalario Universitario de Santiago, Servicio Galego de Saúde, SERGAS, Santiago De Compostela, Spain.

72 University of California San Diego, Moores Cancer Center, La Jolla, CA, USA.

73 Department of Urology, Erasmus University Medical Center, Rotterdam, the Netherlands.

74 Cancer & Environment Group, Center for Research in Epidemiology and Population Health (CESP), INSERM, University Paris-Sud, University Paris-Saclay, Villejuif, France.

75 Clinical Gerontology Unit, University of Cambridge, Cambridge, UK.

76 Division of Genetic Epidemiology, Department of Medicine, University of Utah School of Medicine, Salt Lake City, Utah, USA.

77 George E. Wahlen Department of Veterans Affairs Medical Center, Salt Lake City, UT, USA.

78 The University of Surrey, Guildford, Surrey, UK.

79 Department of Laboratory Medicine and Pathology, Mayo Clinic, Rochester, MN, USA.

**Funding Acknowledgements**

Genotyping of the OncoArray was funded by the US National Institutes of Health (NIH) [U19 CA 148537 for ELucidating Loci Involved in Prostate cancer SuscEptibility (ELLIPSE) project and X01HG007492 to the Center for Inherited Disease Research (CIDR) under contract number HHSN268201200008I]. Additional analytic support was provided by NIH NCI U01 CA188392 (PI: Schumacher).

The PRACTICAL consortium was supported by Cancer Research UK Grants C5047/A7357, C1287/A10118, C1287/A16563, C5047/A3354, C5047/A10692, C16913/A6135, European Commission's Seventh Framework Programme grant agreement n° 223175 (HEALTH-F2-2009-223175), and The National Institute of Health (NIH) Cancer Post-Cancer GWAS initiative grant: No. 1 U19 CA 148537-01 (the GAME-ON initiative).

We would also like to thank the following for funding support: The Institute of Cancer Research and The Everyman Campaign, The Prostate Cancer Research Foundation, Prostate Research Campaign UK (now Prostate Action), The Orchid Cancer Appeal, The National Cancer Research Network UK, The National Cancer Research Institute (NCRI) UK. We are grateful for support of NIHR funding to the NIHR Biomedical Research Centre at The Institute of Cancer Research and The Royal Marsden NHS Foundation Trust.
