# Supplementary material for: Investigating the effects of lycopene and green tea on the metabolome of men at risk of prostate cancer: The ProDiet randomised controlled trial
Source: Int J Cancer. 2018 Dec 7;144(8):1918–28. doi: 10.1002/ijc.31929 (PMC6491994; doi:10.1002/ijc.31929)
Supplement: Supplementary file 2 — Appendix S2: Supporting Information [file IJC-144-1918-s002.docx]

**Supplementary Methods**

**The ProDiet randomised controlled trial**

The ProDiet RCT (ISRCTN 95931417) was nested within the Prostate Cancer testing and Treatment study (ProtecT). It was a randomised feasibility trial, which aimed to establish the feasibility and acceptance of specific dietary interventions in men with elevated prostate specific antigen (PSA) levels (29).

Between December 2009 and May 2010, 113 men between the ages of 50 and 69 years, with PSA results between 2.0 and 2.95ng/ml or at least 3.0ng/ml with a negative biopsy, were recruited into the trial from nine primary care practices in the South West of England. The selected PSA thresholds were based on previous findings that men with modestly raised PSA levels had an increased risk of prostate cancer (32).

Trial participants were randomised (in a 2 x 3 factorial design) to daily lycopene (active capsule, lycopene-rich diet or placebo capsule) and green tea (active capsule, tea drink or placebo capsule) for six months, as described previously (1). The men were blinded to which capsule they received (active or placebo). Blinding of participants to the dietary intervention was not possible in this study for practical reasons.

Men were found to adhere successfully to both lycopene and green tea dietary interventions, as evidenced by important increases in circulating levels of lycopene and EGCG (a bioactive component of green tea) in the diet and supplement groups compared to the placebo groups.

**Dietary assessment**

Men reported frequency of intake for each of the food item (n=114) across nine categories, ranging from “never/less than once per month” to “six or more times per day”. Information on portion sizes and nutrient intake were derived for each food item using *McCance* *and Widdowson’s Composition of Foods* (2), as described previously (3)*.* Food intake was computed as the product of frequency of intake and nutrient content per portion of food. Green tea consumption was assessed in terms of frequency of intake (daily versus not). Lycopene-rich food items included tomatoes, tomato ketchup, tomato juice, pizza and baked beans. The lycopene content of foods was obtained from the European carotenoid database compiled by O’Neill and colleagues (4).

**Measurement of metabolic traits**

The metabolic profiles of all samples included in this analysis were assessed using a high-throughput serum nuclear magnetic resonance (NMR) metabolomics platform, originally described in (5, 6) and reviewed in more detail in (7, 8). The platform detects over 220 metabolic biomarkers, including: amino acids, glycolysis measures, ketone bodies and fatty acids, as well as the lipid concentrations and compositions of 14 lipoprotein subclasses. The 14 lipoprotein subclass sizes are defined as follows: very low density lipoprotein (VLDL) is subdivided into six subclasses, the largest being extremely large VLDL with particle diameters from 75 nm upwards and a possible contribution of chylomicrons, and five remaining VLDL subclasses (average particle diameters of 64.0 nm, 53.6 nm, 44.5 nm, 36.8 nm, and 31.3 nm); Intermediate density lipoprotein (IDL) (28.6 nm), three low density lipoprotein (LDL) subclasses (25.5 nm, 23.0 nm, and 18.7 nm), and four high density lipoprotein (HDL) subclasses (14.3 nm, 12.1 nm, 10.9 nm, and 8.7 nm). The mean sizes for VLDL, LDL and HDL particles were calculated by weighting the corresponding subclass diameters with their particle concentrations (9).

This platform has been applied in various large-scale epidemiological and genetic studies (6, 9, 10).

There exists high analytical consistency between metabolic measures quantified by the NMR metabolomics platform and concentrations obtained from routine clinical chemistry (8) or other analytical methods, e.g. gas chromatography (8, 10) and enzymatic methods (8), with correlations >0.9. Previous work has also demonstrated consistency of biomarker associations with disease incidence for metabolic traits quantified by NMR and two widely used mass spectroscopy platforms (8, 10)**.**

**Mendelian randomization**

Mendelian randomization is an application of the technique of instrumental variable (IV) analysis, whereby genetic variants are used as instrumental variables (IVs) (i.e. proxies for the exposure of interest) to examine the causal effect of a modifiable exposure on an outcome of interest (11, 12). Mendelian randomization is based on a number of assumptions: firstly, that the IV is associated with an exposure of interest; secondly, that the IV is independent of confounding between the exposure and the outcome, and thirdly that the IV is not associated with the outcome of interest – except through its association with the exposure of interest (12). Utilization of this approach depends on the existence of genetic variants that are robustly associated with metabolic trait levels.

We first identified genetic variants that are robustly associated with our metabolic traits of interest, using data from a recently published genome-wide association study (GWAS) of 123 circulating metabolic traits (13) levels. Independent single nucleotide polymorphism (SNP) effects that met genome-wide significance (p < 5x10^-^8) were used as genetic instruments. To leverage power for the Mendelian randomization analysis, we obtained summary data from a GWAS for N= 44,825 cases and N= 27,904 controls of European ancestry in the PRACTICAL (Prostate Cancer Association Group to Investigate Cancer Associated Alterations in the Genome) consortium (14), to establish the gene-outcome associations. We performed a look-up of all SNPs associated with the metabolic traits of interest in the prostate cancer GWAS summary data. We extracted the following summary data for each SNP: the log odds ratio (OR) per copy of the effect allele, its standard error, the effect allele and the non-effect allele.

We combined information from the genome-outcome associations from PRACTICAL with information on the gene-exposure associations from the GWAS of metabolite levels in a two-sample Mendelian randomization approach (15). We calculated the log OR for prostate cancer per standard deviation (SD) unit increase in metabolic trait levels using the Wald ratio method (SEs calculated using delta method), and validated findings using two further tests: likelihood based methods and MR Egger (16, 17).

**References**

1. Horwood JP, Avery KN, Metcalfe C, Donovan JL, Hamdy FC, Neal DE, et al. Men's knowledge and attitudes towards dietary prevention of a prostate cancer diagnosis: a qualitative study. BMC Cancer. 2014;14:812.

2. Lane JA, Donovan JL, Davis M, Walsh E, Dedman D, Down L, et al. Active monitoring, radical prostatectomy, or radiotherapy for localised prostate cancer: study design and diagnostic and baseline results of the ProtecT randomised phase 3 trial. Lancet Oncol. 2014;15(10):1109-18.

3. Er V, Lane JA, Martin RM, Emmett P, Gilbert R, Avery KN, et al. Adherence to dietary and lifestyle recommendations and prostate cancer risk in the prostate testing for cancer and treatment (ProtecT) trial. Cancer Epidemiol Biomarkers Prev. 2014;23(10):2066-77.

4. O'Neill ME, Carroll Y, Corridan B, Olmedilla B, Granado F, Blanco I, et al. A European carotenoid database to assess carotenoid intakes and its use in a five-country comparative study. Br J Nutr. 2001;85(4):499-507.

5. Soininen P, Kangas AJ, Wurtz P, Tukiainen T, Tynkkynen T, Laatikainen R, et al. High-throughput serum NMR metabonomics for cost-effective holistic studies on systemic metabolism. Analyst. 2009;134(9):1781-5.

6. Kettunen J, Tukiainen T, Sarin AP, Ortega-Alonso A, Tikkanen E, Lyytikainen LP, et al. Genome-wide association study identifies multiple loci influencing human serum metabolite levels. Nature genetics. 2012;44(3):269-76.

7. Soininen P, Kangas AJ, Wurtz P, Suna T, Ala-Korpela M. Quantitative serum nuclear magnetic resonance metabolomics in cardiovascular epidemiology and genetics. Circ Cardiovasc Genet. 2015;8(1):192-206.

8. Würtz P, Kangas, A. J., Soininen, P., Lawlor, D. A., Davey Smith, G., & Ala-Korpela, M. . Quantitative Serum NMR Metabolomics in Large-Scale Epidemiology: A Primer on-Omic Technology. American Journal of Epidemiology. 2017;kwx016.

9. Kujala UM, Makinen VP, Heinonen I, Soininen P, Kangas AJ, Leskinen TH, et al. Long-term leisure-time physical activity and serum metabolome. Circulation. 2013;127(3):340-8.

10. Wurtz P, Havulinna AS, Soininen P, Tynkkynen T, Prieto-Merino D, Tillin T, et al. Metabolite profiling and cardiovascular event risk: a prospective study of 3 population-based cohorts. Circulation. 2015;131(9):774-85.

11. Lawlor DA, Harbord RM, Sterne JA, Timpson N, Davey Smith G. Mendelian randomization: using genes as instruments for making causal inferences in epidemiology. Stat Med. 2008;27(8):1133-63.

12. Didelez V, Sheehan N. Mendelian randomization as an instrumental variable approach to causal inference. Stat Methods Med Res. 2007;16(4):309-30.

13. Kettunen J, Demirkan A, Wurtz P, Draisma HH, Haller T, Rawal R, et al. Genome-wide study for circulating metabolites identifies 62 loci and reveals novel systemic effects of LPA. Nat Commun. 2016;7:11122.

14. Kote-Jarai Z, Easton DF, Stanford JL, Ostrander EA, Schleutker J, Ingles SA, et al. Multiple novel prostate cancer predisposition loci confirmed by an international study: the PRACTICAL Consortium. Cancer Epidemiol Biomarkers Prev. 2008;17(8):2052-61.

15. Pierce BL, Burgess S. Efficient design for Mendelian randomization studies: subsample and 2-sample instrumental variable estimators. Am J Epidemiol. 2013;178(7):1177-84.

16. Bowden J, Davey Smith G, Burgess S. Mendelian randomization with invalid instruments: effect estimation and bias detection through Egger regression. Int J Epidemiol. 2015;44(2):512-25.

17. Burgess S, Dudbridge F, Thompson SG. Combining information on multiple instrumental variables in Mendelian randomization: comparison of allele score and summarized data methods. Stat Med. 2016;35(11):1880-906.
